# HIV-Resistant and HIV-Specific CAR-Modified CD4^+^ T Cells Mitigate HIV Disease Progression and Confer CD4^+^ T Cell Help *In Vivo*

**DOI:** 10.1016/j.ymthe.2020.05.012

**Published:** 2020-05-15

**Authors:** Colby R. Maldini, Kevin Gayout, Rachel S. Leibman, Derrick L. Dopkin, Joshua P. Mills, Xiaochuan Shan, Joshua A. Glover, James L. Riley

**Affiliations:** 1 Department of Microbiology, Center for Cellular Immunotherapies, Perelman School of Medicine, University of Pennsylvania, Philadelphia, PA 19104, USA; 2 Deparment of Pathology & Laboratory Medicine, Perelman School of Medicine, University of Pennsylvania, Philadelphia, PA 19104, USA

**Keywords:** chimeric antigen receptor, T cell help, IL-21, IL-2, in vivo model of HIVPD-1, TIGIT, costimulation, C34-CXCR4

## Abstract

HIV infection preferentially depletes HIV-specific CD4^+^ T cells, thereby impairing antiviral immunity. In this study, we explored the therapeutic utility of adoptively transferred CD4^+^ T cells expressing an HIV-specific chimeric antigen receptor (CAR_4_) to restore CD4^+^ T cell function to the global HIV-specific immune response. We demonstrated that CAR_4_ T cells directly suppressed *in vitro* HIV replication and eliminated virus-infected cells. Notably, CAR_4_ T cells containing intracellular domains (ICDs) derived from the CD28 receptor family (ICOS and CD28) exhibited superior effector functions compared to the tumor necrosis factor receptor (TNFR) family ICDs (CD27, OX40, and 4-1BB). However, despite demonstrating limited *in vitro* efficacy, only HIV-resistant CAR_4_ T cells expressing the 4-1BBζ ICD exhibited profound expansion, concomitant with reduced rebound viremia after antiretroviral therapy (ART) cessation and protection of CD4^+^ T cells (CAR^−^) from HIV-induced depletion in humanized mice. Moreover, CAR_4_ T cells enhanced the *in vivo* persistence and efficacy of HIV-specific CAR-modified CD8^+^ T cells expressing the CD28ζ ICD, which alone exhibited poor survival. Collectively, these studies demonstrate that HIV-resistant CAR_4_ T cells can directly control HIV replication and augment the virus-specific CD8^+^ T cell response, highlighting the therapeutic potential of engineered CD4^+^ T cells to engender a functional HIV cure.

## Graphical Abstract

**Figure:**
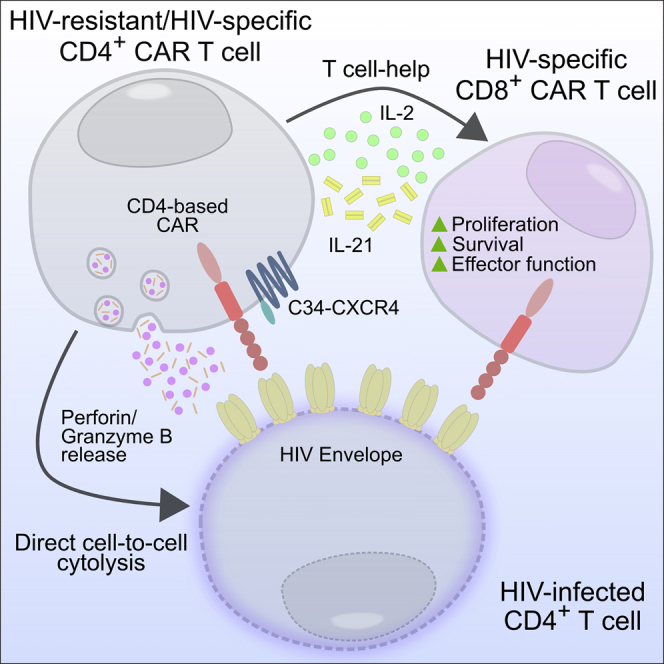

## Teaser

HIV infects CD4^+^ T cells, resulting in the impairment of antiviral immunity and susceptibility to opportunistic diseases. Maldini and colleagues investigated the therapeutic utility of gene-engineered, HIV-resistant/specific CD4^+^ T cells to directly combat infection and restore T cell help that is lost due to HIV-mediated destruction of endogenous CD4^+^ T cells.

## Introduction

HIV infection induces profound CD4^+^ T cell loss, resulting in impaired antiviral immunity and the onset of overt immunodeficiency.^1^ In particular, HIV-specific CD4^+^ T cells are preferentially infected and exhibit defective immune responses characterized by poor proliferative capacity and interleukin (IL)-2 secretion.^2^, ^3^, ^4^, ^5^ The collapse of CD4^+^ T cell help during chronic infections compromises the generation of cytotoxic and memory CD8^+^ T cells,^6^, ^7^, ^8^ leads to pronounced CD8^+^ T cell exhaustion,^6^^,^^9^ and diminishes effective antibody production.^10^^,^^11^ Moreover, CD4^+^ T cells exhibit direct cell-to-cell-mediated effector functions that contribute to disease resolution.^12^, ^13^, ^14^, ^15^ Indeed, subjects who spontaneously control HIV replication demonstrate a significant expansion of cytolytic CD4^+^ T cells that are associated with slower disease progression and improved clinical outcomes.^15^^,^^16^ Given the functional heterogeneity of HIV-specific CD4^+^ T cells and their ability to coordinate global antiviral immunity, therapeutic interventions that restore or augment CD4^+^ T cell function will likely be critical for the development of effective HIV cure strategies.

Emerging data from cancer models demonstrate that CD4^+^ T cells redirected with a chimeric antigen receptor (CAR_4_) can eradicate tumors in the absence of other immune cells,^17^, ^18^, ^19^ indicating that CAR_4_ T cells can act as primary effectors in addition to providing help to other leukocytes. CARs confer novel T cell specificity through expression of an extracellular antigen-binding domain fused to the intracellular CD3ζ chain and one or more costimulatory domains.^20^^,^^21^ The choice of costimulatory domain alters the metabolic, phenotypic, and functional CAR T cell profile.^22^^,^^23^ For instance, the CD28 costimulatory domain promotes glycolytic metabolism and the acquisition of an effector memory T cell phenotype capable of exhibiting rapid antitumor activity, whereas the 4-1BB domain supports the long-term *in vivo* persistence and development of central memory T cells reliant on oxidative phosphorylation for energy.^24^, ^25^, ^26^ As such, the ability of CARs to engender unique T cell traits suggests that adoptively transferred HIV-specific CAR_4_ T cells can restore many of the functions lost by HIV-induced destruction of CD4^+^ T cells.

However, since CD4^+^ T cells are the primary targets of HIV, efforts to make CAR_4_ T cells resistant to infection must be employed to ensure durable responses.^27^ Several approaches, including HIV coreceptor disruption,^28^, ^29^, ^30^ as well as the overexpression of restriction factors^31^^,^^32^ and fusion inhibitors,^33^^,^^34^ have been developed to confer HIV resistance. Of these, the fusion inhibitor C34 linked to CXCR4 is potent and interferes with the entry of diverse HIV strains regardless of their tropism.^35^ Because of the need to protect and redirect CD4^+^ T cells, progress to engineer the best CD4^+^ T cells to control HIV replication has lagged behind studies examining CD8^+^ T cells. Nonetheless, HIV-specific CAR_8_ T cells have shown promise to inhibit virus replication, thereby providing the tools necessary to study CAR_4_ T cells. For instance, we recently reported on an HIV-specific CAR that expresses CD4 as the HIV_ENV_ binding motif and contains the 4-1BB costimulatory domain.^36^ This CAR was re-engineered from the original construct used during the first in-human clinical trials,^37^, ^38^, ^39^ and it was selected for its improved antiviral potency in CD8^+^ T cells^36^ and potential to minimize virus escape.^40^ Other approaches have incorporated a similar CD4-based CAR into CD34^+^ hematopoietic stem cells to provide long-term *in vivo* production of HIV-specific immune cells,^41^^,^^42^ or they have targeted infected cells using alterative antigen-binding moieties.^43^, ^44^, ^45^, ^46^ Collectively, these strategies highlight the promise of CAR T cell-based therapies against HIV; however, critical knowledge gaps remain, especially in our understanding of whether CAR_4_ T cells need to be engineered separately from CAR_8_ T cells to achieve the optimal HIV-specific response.

In this study, we explored the therapeutic potential of CAR_4_ T cell therapy to mitigate HIV pathogenesis. Data from cancer studies indicate that CAR_4_ T cells require different costimulatory signals than CAR_8_ T cells to engender superior immune responses.^47^^,^^48^ However, using a panel of HIV-specific (CD4-based) CARs expressing distinct intracellular domains (ICDs), we show that only HIV-resistant, 4-1BB-costimulated CAR_4_ T cells limit *in vivo* HIV infection, congruent with our previous work identifying 4-1BB as the optimal ICD for HIV-specific CAR_8_ T cells.^36^ Notably, ICDs derived from the CD28 receptor family (ICOS and CD28), which conferred the greatest CAR_4_ T cell effector function *in vitro*, did not induce protective responses in humanized mice, suggesting that favorable disease outcomes are associated with factors beyond *in vitro* efficacy. These results, together with the observation that HIV-specific CAR_4_ T cells augment the CD8^+^ T cell response to infection, highlight the importance of exploiting engineered CD4^+^ T cells in immunotherapies intended to treat chronic infections.

## Results

### Costimulatory Domains Differentially Modulate HIV-Specific CAR_4_ T Cell Cytokine Production

**Figure 1:**
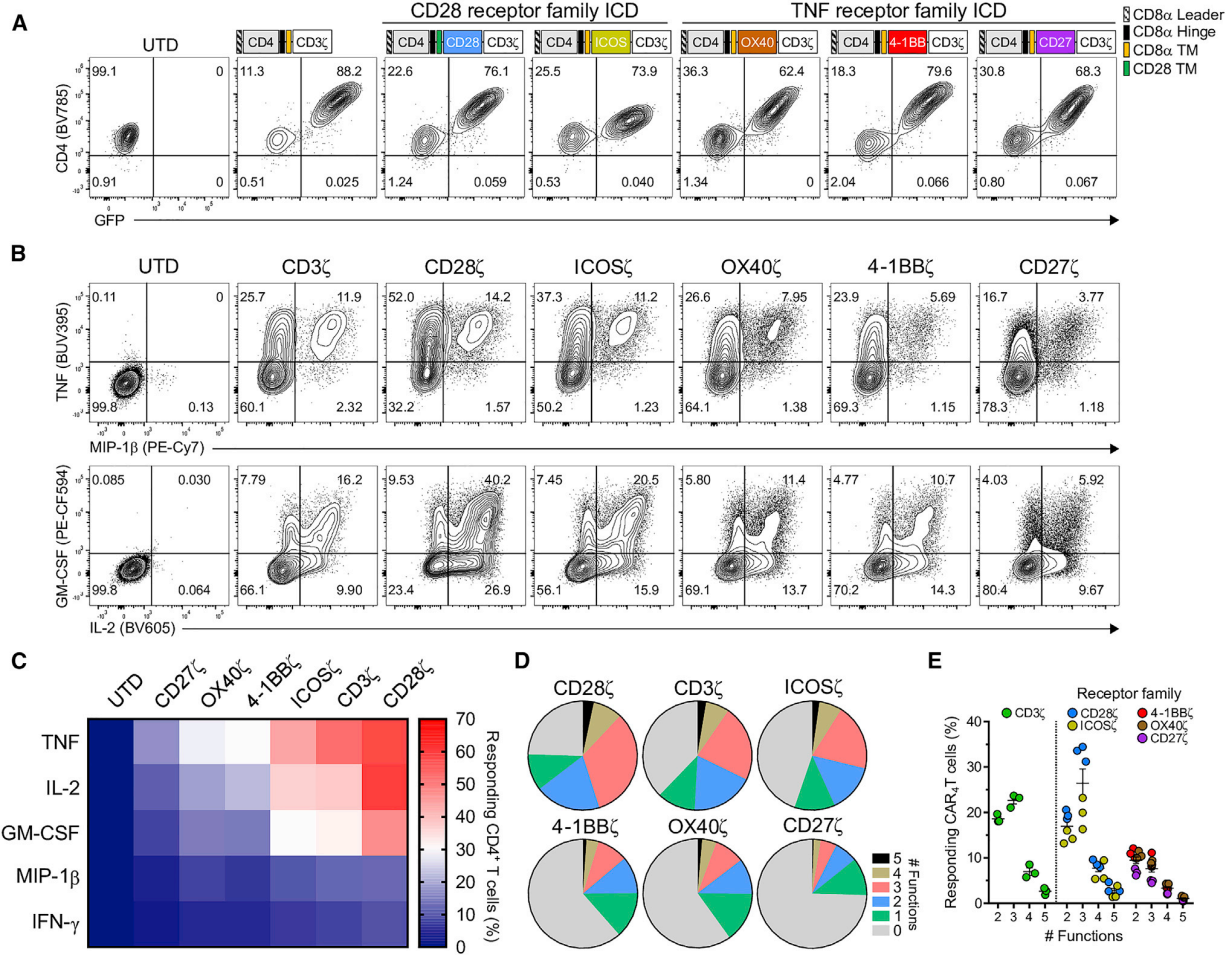
Intracellular Costimulatory Domains Differentially Modulate HIV-Specific CAR_4_ T Cell Cytokine Expression (A) Schematic representation of each CAR construct and fluorescence-activated cell sorting (FACS) plots identifying HIV-specific CAR-modified CD4^+^ T cells (CAR_4_) as GFP^+^ and CD4^+^ relative to untransduced CD4^+^ T cells (UTD_4_). Purified human CD4^+^ T cells from a healthy human donor were activated with anti-CD3/CD28 Dynabeads and transduced with a lentiviral vector encoding one of six HIV-specific (CD4-based) CARs that express unique intracellular domains (ICDs), either CD3ζ (ζ), 4-1BBζ, CD28ζ, CD27ζ, OX40ζ, or ICOSζ. Each CAR was linked to GFP by an intervening T2A sequence to facilitate *in vitro* detection. (B) After 10 days of expansion, CAR_4_ T cells were *in vitro* stimulated with HIV_YU2_ GFP160^+^ K562 cells (K.Env), and intracellular cytokine analysis was performed. Data are representative of three donors. (C) Heatmap showing the percentage of responding CAR_4_ T cells for each of the indicated cytokines. (D) Polyfunctionality profiles of combinatorial subsets for CAR_4_ T cells producing 0–5 human cytokines: TNF, IL-2, IFN-γ, GM-CSF, and MIP-1β. (C and D) Data are the average of three donors. (E) Summary data of three donors per CAR_4_ T cell population producing two or more cytokine functions after antigen stimulation. Lines indicate mean, and error bars show ±SEM.

To engineer optimal HIV-specific CD4^+^ T cells for use in HIV cure strategies, we first generated an array of CAR_4_ T cells that expressed CD4, the natural ligand of HIV_ENV_, as the extracellular antigen-binding moiety fused to an ICD comprising the TCR CD3ζ (ζ) chain and one costimulatory domain derived from CD27, OX40, 4-1BB, ICOS, or CD28. The CAR containing the CD28ζ ICD was linked to the CD28 transmembrane (TM) domain, while the remaining CARs contained the CD8α TM domain. All of the HIV-specific CARs were efficiently expressed on the cell surface and could be identified by both the overexpression of CD4 relative to untransduced CD4^+^ T cells (UTD_4_) and co-expression of GFP, which was linked to each CAR by an intervening T2A sequence (Figure 1A).

We then assessed the effects of individual ICDs on the *in vitro* effector function of CAR_4_ T cells after antigen-specific stimulation with target cells expressing HIV_ENV_. All of the CAR_4_ T cell types upregulated tumor necrosis factor (TNF), macrophage-inflammatory protein (MIP)-1β, granulocyte-macrophage colony-stimulating factor (GM-CSF), IL-2 and interferon (IFN)-γ following antigen exposure; however, CAR_4_ T cells containing the CD28 receptor family ICDs exhibited the greatest production of these cytokines (Figures 1B and 1C; Figure S1). In particular, more than 75% of CD28-costimulated CAR_4_ T cells elicited a cytokine response to stimulation, exemplified by IL-2 and TNF expression. Interestingly, we observed low expression levels of other cytokines associated with T helper phenotypes, including IL-17A, IL-21, IL-22, IL-4, and IL-13 (Figure S2). Moreover, combinatorial cytokine analysis indicated that ICDs from the CD28 receptor family increased the frequency of polyfunctional responses (Figure 1D), where exhibiting three or more cytokine functions contributed more than 20% of the total response and was mainly driven by the GM-CSF^+^IL-2^+^TNF^+^ subset, compared to less than 10% by the TNF receptor (TNFR) family ICDs (CD27, OX40, and 4-1BB) (Figures 1D and 1E). Somewhat paradoxically, in the absence of costimulation, the CD3ζ ICD induced robust cytokine production (Figures 1C–1E), which may result from the high expression of this CAR on the CD4^+^ T cell surface (Figure S3). Taken together, these data demonstrate that HIV-specific CARs redirect CD4^+^ T cell specificity, and distinct costimulatory signals within the CAR differentially modulate the magnitude and breadth of cytokine expression.

### HIV-Specific CAR_4_ T Cells Durably Suppress *In Vitro* HIV Replication

**Figure 2:**
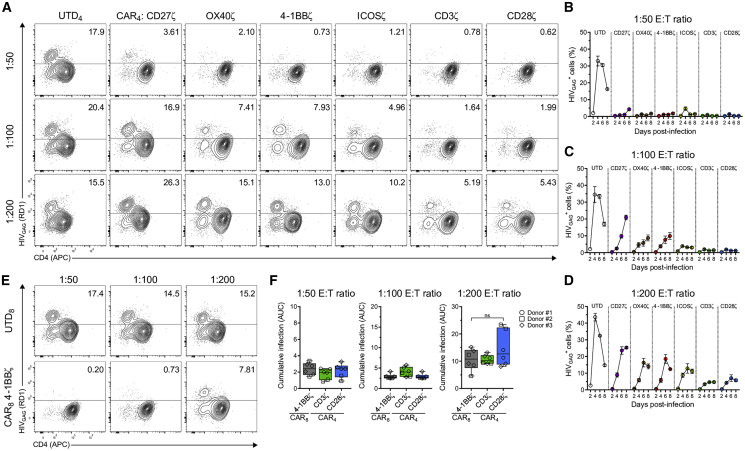
HIV-Specific CAR_4_ T Cells Durably Suppress *In Vitro* HIV Replication HIV-specific CAR T cells and UTD T cells were mixed separately with HIV_BAL_-challenged CD4^+^ T cells at the indicated effector-to-target (E:T) ratios, and the level of virus spread was monitored by intracellular staining and flow cytometry for HIV_GAG_ antigen on days 2, 4, 6, and 8 after co-culture. (A) FACS plots indicate the frequency of HIV_GAG_^+^ cells (CAR^−^) 8 days after co-culture with each CAR_4_ T cell type or UTD_4_ T cells. (B–D) Summary of the frequency of HIV_GAG_^+^ cells at days 2, 4, 6, and 8 after co-culture at (B) 1:50, (C) 1:100, and (D) 1:200 E:T ratios. For (B)–(D), symbols represent the average of three distinct donors in duplicate, and error bars show ±SEM. (E) FACS plots show the frequency of HIV_GAG_^+^ cells (CAR^−^CD8^−^) 8 days after co-culture with UTD_8_ or HIV-specific CAR_8_ T cells expressing the 4-1BBζ intracellular domain. (F) Cumulative infection calculated by area under the curve from the frequency of HIV_GAG_^+^ cells at days 2, 4, 6, and 8 after co-culture. Data are represented as box-and-whisker plots and bars show minimum and maximum values. Symbols indicate unique donors performed in duplicate. A Kruskal-Wallis test and Dunn’s multiple comparison test were used to determine significance. ns, not significant (p > 0.05).

To determine whether HIV-specific CAR_4_ T cells could limit the spread of HIV infection, we performed an *in vitro* suppression assay comparing the ability CAR_4_ T cells containing different ICDs to inhibit virus outgrowth. In this study, activated CD4^+^ T cells were infected with HIV_BAL_ for 24 h before co-culture with CAR_4_ or UTD_4_ T cells at different effector-to-target (E:T) ratios, and virus replication was measured during 8 days by determining the frequency of intracellular HIV_GAG_^+^ (CAR^−^) cells. We observed widespread virus replication when infected cells were cultured with UTD_4_ T cells, whereas each CAR_4_ T cell population durably suppressed HIV at a 1:25 and 1:50 E:T ratio (Figures S4A and S4B; Figures 2A and 2B). However, further dilution of CAR_4_ T cells expressing the TNFR family ICDs resulted in rapid loss of virus control (Figures 2A and 2C; Figure S4C). In contrast, CAR_4_ T cells containing the CD28 receptor family and CD3ζ ICDs potently suppressed virus spread at lower E:T ratios (Figures 2C and 2D; Figure S4D).

At the same time, we compared the protective role of CAR_4_ T cells to that of HIV-specific CAR_8_ T cells expressing the 4-1BBζ ICD. We previously demonstrated that 4-1BB costimulation conferred optimal CAR_8_ T cell antiviral activity *in vivo*,^36^ and now this construct is currently used in a phase I clinical trial (ClinicalTrials.gov: NCT03617198). CAR_8_ T cells exhibited potent HIV suppression below a 1:100 E:T ratio (Figure 2E; Figure S5A), and, notably, CAR_4_ T cells expressing the CD28ζ and CD3ζ ICDs controlled virus spread to the same extent (Figure 2F). For a direct comparison, we noted that CAR_4_ T cells expressing the 4-1BBζ ICD were approximately 3-fold less suppressive than CAR_8_ T cells harboring the same ICD (Figure S5B). These results indicated that HIV-specific CAR_4_ T cells can solely control *in vitro* HIV replication, and that the ICD integrated into the CAR modulates the ability of CAR_4_ T cells to directly inhibit virus outgrowth.

### HIV-Specific CAR_4_ T Cells Directly Eliminate HIV-Infected Cells *In Vitro*

**Figure 3:**
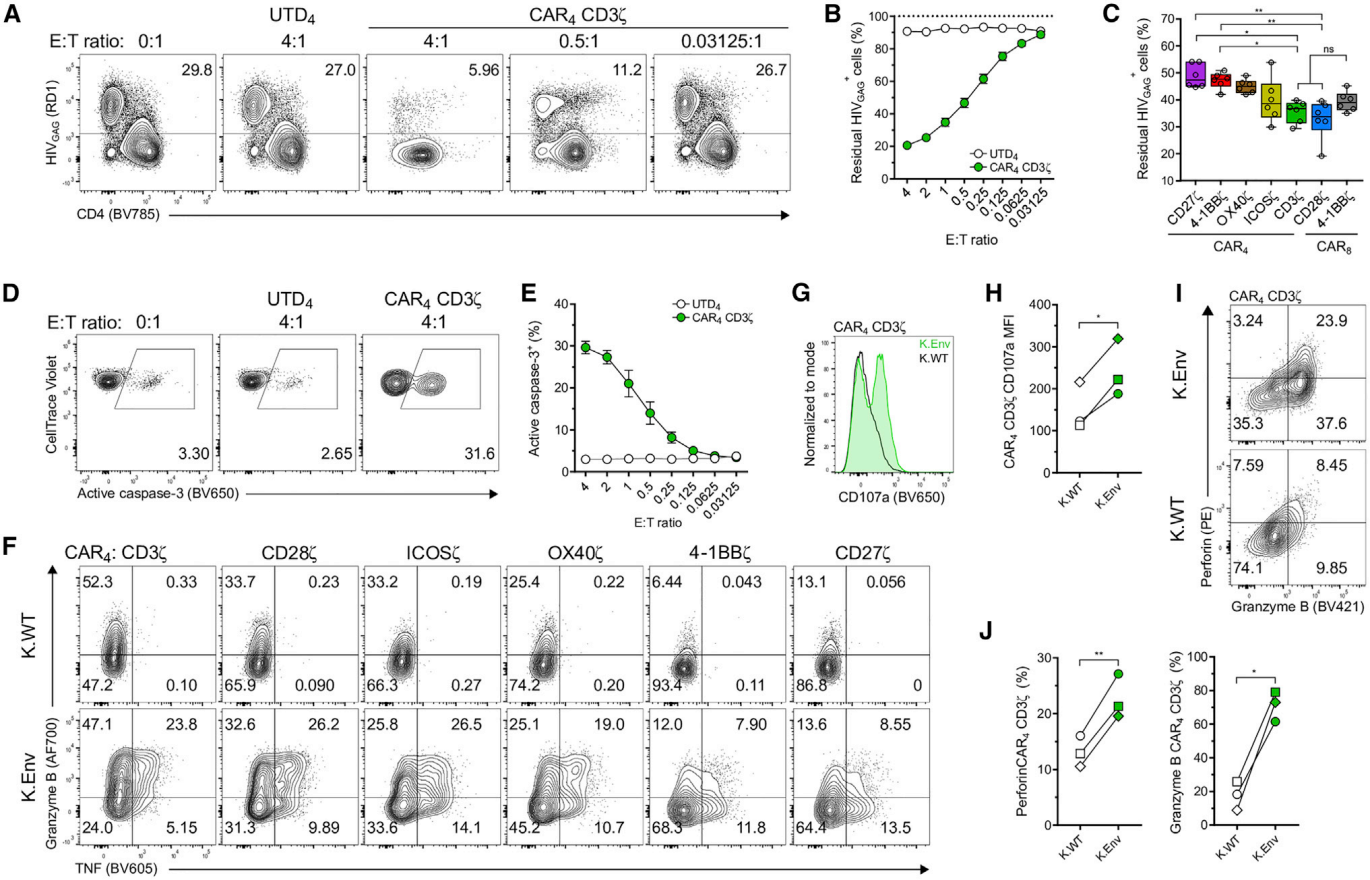
HIV-Specific CAR_4_ T Cells Exhibit Cytolytic Function against Virus-Infected CD4^+^ T Cells (A–E) CellTrace Violet-labeled, HIV_BAL_-infected CD4^+^ T cells (30% HIV_GAG_^+^) were cultured with HIV-specific CAR or UTD T cells at the indicated E:T ratios. 24 h later the frequency of HIV_GAG_^+^ cells (live CTV^+^CAR^−^) was assessed by intracellular staining and flow cytometry for HIV_GAG_ antigen. (A and B) FACS plots show the frequency of HIV_GAG_^+^ cells (A), and summary data indicate the frequency of residual HIV_GAG_^+^ cells after co-culture with UTD_4_ or CAR_4_ T cells expressing the CD3ζ ICD (B). (C) Summary data of elimination assay at the 0.125:1 E:T ratio. (D and E) FACS plots (D) and summary data (E) show frequency of active caspase-3 within HIV_GAG_^+^ cells after co-culture with UTD_4_ or CAR_4_ T cells expressing the CD3ζ ICD. (F) After 10 days of expansion, CAR_4_ T cells were stimulated with K.Env or wild-type K562 cells (K.WT), and FACS plots show upregulation of granzyme B and TNF. Data are representative of three donors. (G and H) Histogram (G) and mean fluorescence intensity (MFI) (H) indicate CD107a mobilization in CAR_4_ T cells after *in vitro* stimulation. (I and J) FACS plots (I) and summary data (J) show the coordinated upregulation of perforin and granzyme B in CAR_4_ T cells expressing the CD3ζ ICD after *in vitro* stimulation. (B and E) Symbols indicate average of three donors performed in triplicate. Error bars show ±SEM. (C) Data are represented as box-and-whisker plots, and bars show minimum and maximum values. Symbols indicate three donors performed in duplicate. A Kruskal-Wallis test and Dunn’s multiple comparison test were used to determine significance. (H and J) Data show three donors, and significance was calculated using a paired Student’s t test. ∗p < 0.05, ∗∗p < 0.01 (for all data).

The effector mechanisms employed by CD4^+^ T cells largely fall into two categories: first, the production of broad-acting soluble factors, such as IFN-γ and TNF-α, that promote an antiviral state in surrounding tissue,^49^ and second, direct cytotoxic activity.^50^, ^51^, ^52^ In the context of CD19-targeted CAR T cell therapy, CAR_4_ T cells directly engage and kill tumor cells *in vitro* to the same extent as CAR_8_ T cells, but they exhibit slower kinetics of cytotoxicity.^53^ Similarly, we examined whether HIV-specific CAR_4_ T cells elicit cytotoxic function by performing a short-term killing assay using primary HIV-infected cells as targets.^54^ CAR_4_ T cells expressing the CD3ζ ICD eliminated target cells in a dose-dependent manner, achieving an 80% reduction in the frequency of HIV_GAG_^+^ cells (from 30% to 6% HIV_GAG_^+^ cells) after 24 h (4:1 E:T ratio) (Figures 3A and 3B). The killing exhibited by CAR_4_ T cells was HIV-specific, as indicated by the lack of infected cell lysis when cultured with UTD_4_ T cells (Figure 3B). Furthermore, when we expanded the killing assay to include CAR_4_ T cells encoding distinct costimulatory domains, we observed marked reductions in the number of HIV_GAG_^+^ cells mediated by CAR_4_ T cells expressing the CD28 receptor family ICDs or CD3ζ. Notably, the level of infected cell killing was equivalent between these CAR_4_ T cell types and CAR_8_ T cells (Figure 3C; Figure S6). In addition, inclusion of the TNFR family ICDs conferred cytolytic activity to CAR_4_ T cells, but not to the same magnitude (Figure 3C; Figure S6).

We next examined whether CAR_4_ T cells could trigger activation of caspase-3 in HIV-infected cells, which acts as the primary executioner in the cell-death apoptosis pathway^55^ and is a direct substrate of granzyme (Gzm) B.^56^ Indeed, CAR_4_ T cells induced cleavage of caspase-3 in HIV_GAG_^+^ cells (Figures 3D and 3E), suggesting that virus-infected cell death is mediated in part by caspase-dependent mechanisms. Furthermore, we assessed the expression of cytolytic effector molecules, including GzmB, GzmA, and GzmM, in all CAR_4_ T cell populations. CAR_4_ T cells containing the CD28 receptor family ICDs or CD3ζ harbored the highest level of GzmB and GzmA (Figure 3F; Figure S7A), while GzmM expression was negligible (Figure S7B), suggesting that signaling from ICDs influences the cytotoxic potential of CAR_4_ T cells independent of antigen exposure. However, these molecules were differentially regulated following *in vitro* antigen-specific stimulation, where after we observed a substantial increase in GzmB expression by all CAR_4_ T cell types, exemplified by the CD28 receptor family ICDs or CD3ζ (Figure 3F; Figure S7C), while GzmA expression waned (Figures S7A and S7D). Moreover, stimulation of CAR_4_ T cells resulted in the detection of CD107a, which occurs as cytotoxic granules mobilize from the cytosol to the cell surface (Figures 3G and 3H),^57^ and was coupled with the coordinated upregulation of perforin and GzmB (Figures 3I and 3J). Taken together, these data demonstrate that CAR_4_ T cells exhibit *in vitro* cytotoxic function that can at least be partially attributed to granule-mediated cytolysis.

### 4-1BB-Costimulated CAR_4_ T Cells Mitigate HIV Pathogenesis *In Vivo*

**Figure 4:**
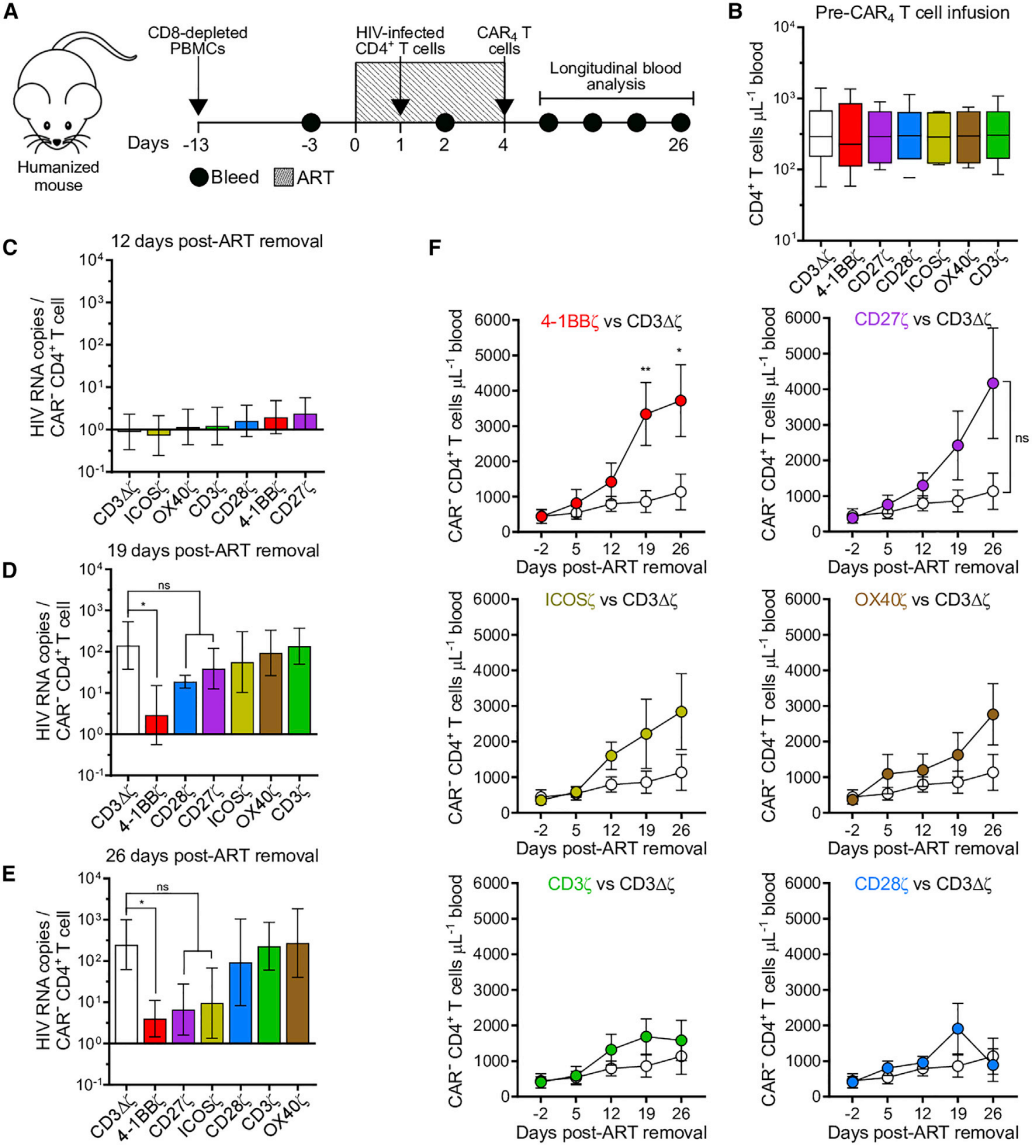
HIV-Specific 4-1BB-Costimulated CAR_4_ T Cells Mitigate HIV Disease Progression after ART Removal (A) Experimental design. NSG mice were infused with CD8-depleted PBMCs from a healthy human donor. Two weeks later, mice initiated daily ART for 1 week and were infused with autologous, *in vitro* HIV_BAL_-infected CD4^+^ T cells. Mice were allocated into seven groups (n = 6–7) based on CD4^+^ T cell engraftment, and then each mouse received 2.5 × 10^6^ HIV-resistant (C34-CXCR4^+^) CAR_4_ T cells expressing one of the six intracellular domains (ICDs), or inactive control CAR_4_ T cells expressing a truncated CD3ζ ICD followed by ART cessation. (B) Concentration of peripheral blood CD4^+^ T cells prior to CAR T cell infusion. Data are represented as box-and-whisker plots, and bars show minimum and maximum values. (C–E) HIV RNA copies/mL plasma normalized to contemporaneous peripheral blood CD4^+^ T cell concentration (CAR^−^) (C) 12 days, (D) 19 days, and (E) 26 days after ART removal. (F) Longitudinal concentration of CD4^+^ T cells (CAR^−^) in peripheral blood. (C–F) Bars and symbols indicate mean, and error bars show ±SEM. Significance was calculated using a Wilcoxon rank sum test. ∗p < 0.05, ∗∗p < 0.01. ns, not significant (p > 0.05).

We next sought to determine the *in vivo* therapeutic potential of distinct HIV-specific CAR_4_ T cell populations using a humanized mouse model of HIV infection.^36^ To do so, we humanized NSG mice by adoptively transferring normal donor, CD8-depleted peripheral blood mononuclear cells (PBMCs). Two weeks later, the mice were infused with autologous, HIV_BAL_-infected CD4^+^ T cells that were treated with antiretroviral therapy (ART) *in vitro* prior to infusion and the mice received daily ART (Figure 4A). After 3 days, mice were allocated into groups based on the level of CD4^+^ T cells in peripheral blood (Figure 4B) and infused with either one of the six CAR_4_ T cell types or control CAR_4_ T cells expressing a truncated CD3ζ ICD (CD3Δζ) (Figure 4A). We rendered each CAR_4_ T cell product HIV-resistant by co-transduction with the HIV fusion inhibitor C34-CXCR4,^35^ which was linked to NGFR (nerve growth factor receptor) by an intervening T2A sequence (Figure S8A). Prior to CAR_4_ T cell infusion, the cells were positively selected for NGFR expression, achieving ≥90% NGFR^+^ (C34-CXCR4^+^) T cell products (Figure S8B). Of note, expression of C34-CXCR4 did not augment the functional potency of CAR T cells, as unprotected CAR T cells suppressed *in vitro* HIV replication to the same extent as CAR_4_ T cells expressing C34-CXCR4 (Figure S8C).

We interrupted ART immediately after CAR_4_ T cell infusion and measured the kinetics of HIV rebound. In this model, the magnitude of virus replication correlates with the number of CD4^+^ T cells,^58^^,^^59^ and thus to ensure fair comparison among treatment groups, we normalized plasma HIV RNA at each time point to the contemporaneous level of peripheral blood CD4^+^ T cells (CAR^−^). We observed that only CAR_4_ T cells expressing the 4-1BBζ ICD reduced the magnitude of rebound viremia for 26 days after ART removal compared to control CAR_4_ T cell-treated mice (Figures 4C–4E; Table S1). This difference remained significant without normalizing viral load, particularly when assessing cumulative viral burden in plasma (Figure S9). Furthermore, mice treated with 4-1BB-costimulated CAR_4_ T cells exhibited durable protection against HIV-induced CD4^+^ T cell (CAR^−^) depletion (Figure 4F). These observations were striking given that the 4-1BBζ ICD underperformed in every *in vitro* measure of CAR_4_ T cell function, especially compared to the CD28 receptor family ICDs.

### 4-1BB-Costimulated CAR_4_ T Cells Exhibit Profound *In Vivo* Expansion

**Figure 5:**
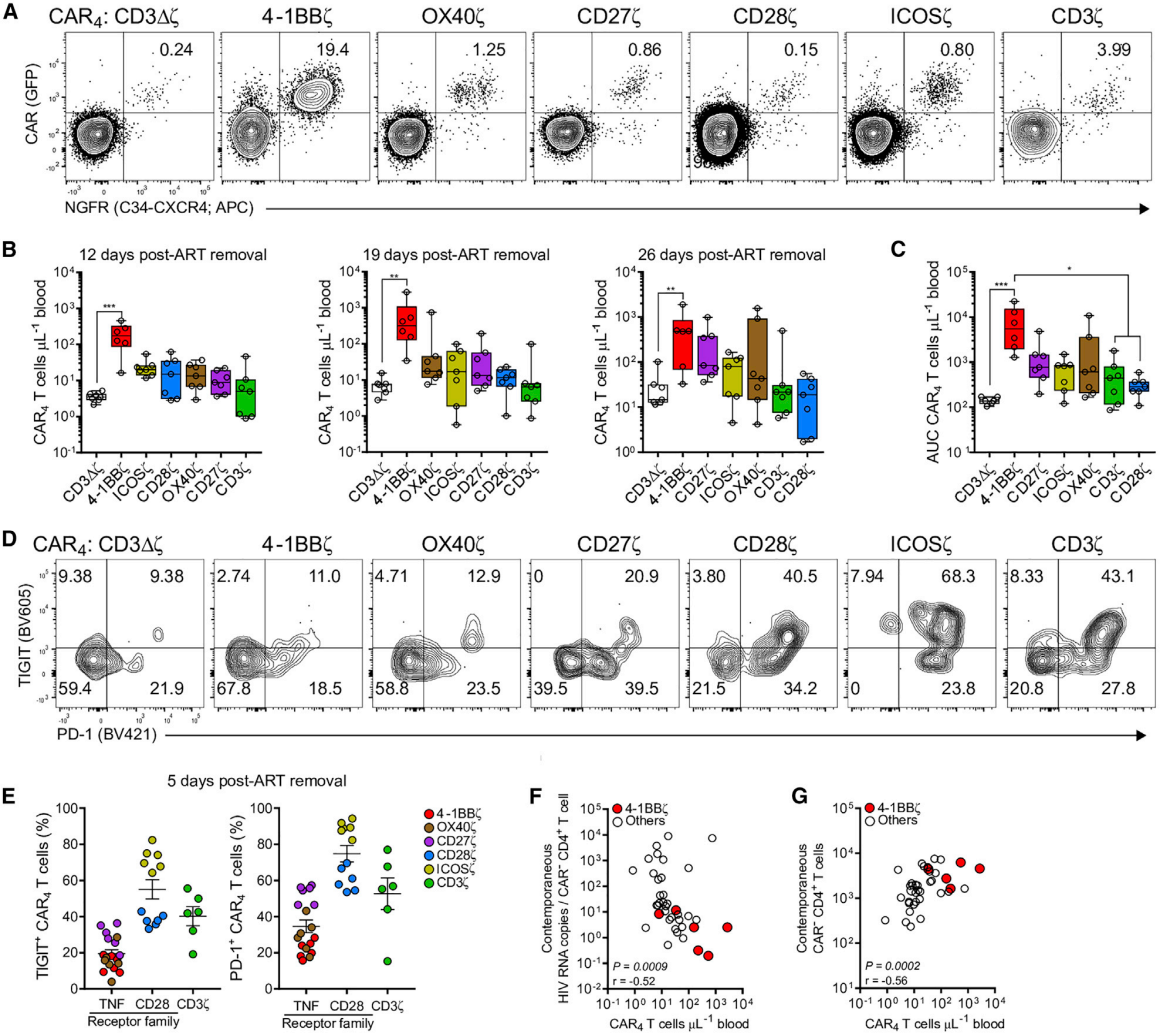
4-1BB Costimulation Potentiates *In Vivo* HIV-Specific CAR_4_ T Cell Expansion and Persistence (A) FACS plots show the detection of each CAR_4_ T cell type in peripheral blood 26 days after ART removal in HIV-infected humanized mice. CAR_4_ T cells are identified by the co-expression of GFP and NGFR, which are linked by intervening T2A sequences to the indicated CD4-based CAR and C34-CXCR4, respectively. (B) Peripheral blood concentration of each CAR_4_ T cell type at days 12, 19, and 26 after ART removal. (C) Cumulative peripheral CAR_4_ T cell persistence measured by area under the curve from days 5, 12, 19, and 26 after ART removal. (D and E) FACS plots (D) and summary data (E) show the frequency of TIGIT and PD-1 expression on each CAR_4_ T cell type 5 days after ART removal. (F and G) Correlation between CAR_4_ T cell concentration 19 days after ART removal and contemporaneous viral load (F), and CD4^+^ T cell (CAR^−^) concentration (G). Red symbols indicate 4-1BB-costimulated CAR_4_ T cells, and white symbols indicate the remaining CAR_4_ T cell types. (B and C) Data are represented as box-and-whisker plots, and bars show minimum and maximum values. Each symbol denotes one mouse. A Wilcoxon rank sum test and (F and G) Spearman correlation were used to test for significance. ∗p < 0.05, ∗∗p < 0.01, ∗∗∗p < 0.001 (for all data).

Next, we characterized the immunologic response inherent to each CAR_4_ T cell type to viral recrudescence after ART cessation. CAR_4_ T cells were readily detected in peripheral blood by the co-expression of GFP and NGFR (Figure 5A). 4-1BB-costimulated CAR_4_ T cells exhibited rapid and profound expansion, reaching a median peak concentration of 511 cells/μL blood (34–2,732 cells/μL) (Figure 5B), and they maintained greater long-term survival compared to the other CAR_4_ T cell populations (Figure 5C). Surprisingly, CAR_4_ T cells expressing CD28 receptor family ICDs, which demonstrated superior *in vitro* potency, exhibited poor *in vivo* expansion and persistence (Figures 5B and 5C). Moreover, CAR_4_ T cells containing the TNFR family ICDs expressed lower levels of inhibitory receptors, including TIGIT and PD-1, early after ART cessation, which may have, in addition to the ICD, modulated early *in vivo* proliferation kinetics (Figures 5D and 5E; Figure S10). Notably, the magnitude of CAR_4_ T cell expansion across all types, but exemplified by the 4-1BBζ ICD, correlated with reductions in plasma viremia (Figure 5F) and CD4^+^ T cell (CAR^−^) preservation (Figure 5G). Taken together, these data demonstrate that CAR_4_ T cells expressing the 4-1BB costimulatory domain possess superior antiviral activity, and they highlight that factors such as expansion potential are critical to mitigate HIV pathogenesis.

### HIV-Specific CAR_4_ T Cells Improve the Proliferation and Survival of Co-injected CAR_8_ T Cells *In Vivo*

**Figure 6:**
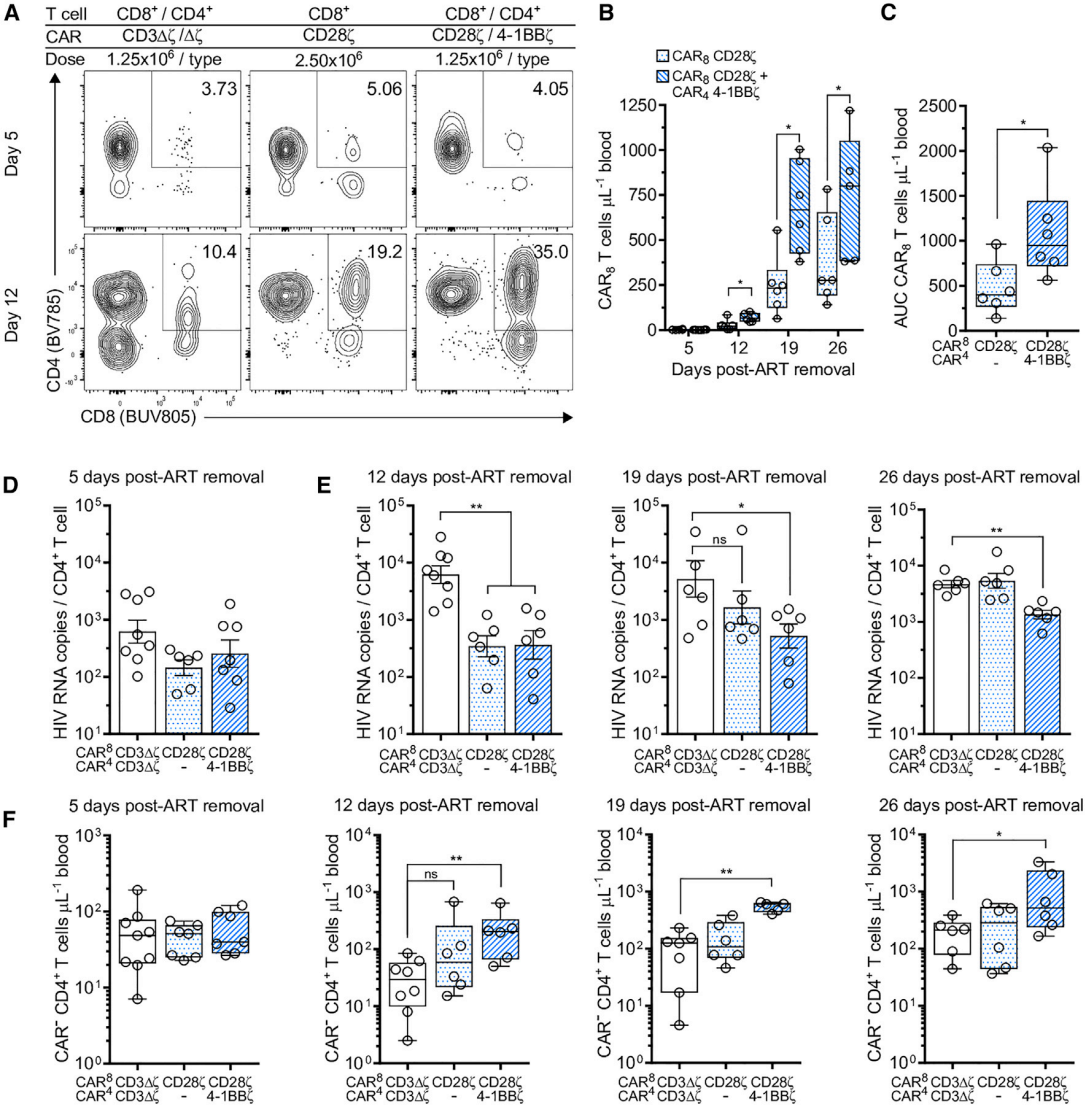
Co-injection of HIV-Specific CAR_4_ T Cells Improves CAR_8_ T Cell Expansion and Post-ART Control of HIV Infection NSG mice were infused with CD8-depleted PBMCs from a healthy human donor. Two weeks later, mice were given ART daily for 1 week and were infused with autologous, *in vitro* HIV_BAL_-infected CD4^+^ T cells. Mice were allocated into three groups (n = 7–8) based on CD4^+^ T cell engraftment, and then each group received either HIV-resistant (C34-CXCR4^+^) CAR_8_ (CD28ζ) T cells (2.5 × 10^6^ CAR^+^ cells/mouse), a 1:1 ratio of CAR_4_ (4-1BBζ) and CAR_8_ (CD28ζ) T cells (1.25 × 10^6^ CAR^+^ cells/cell type/mouse), or a 1:1 ratio of inactive control CAR_4_ and CAR_8_ T cells (1.25 × 10^6^ CAR^+^ cells/cell type/mouse) expressing the CD3Δζ ICD followed by ART interruption. (A) FACS plots show the frequency of peripheral CAR_8_ T cells identified by the overexpression of CD4 on the CD8^+^ T cell surface. (B) Longitudinal concentration of peripheral CAR_8_ T cells after ART interruption. (C) Cumulative peripheral CAR_8_ T cells persistence measured by area under the curve from days 5, 12, 19, and 26 after ART removal. (D and E) HIV RNA copies/mL plasma normalized to contemporaneous peripheral CD4^+^ T cell (CAR^-^) concentration at (D) 5 days and (E) 12, 19, and 26 days after ART removal. (F) Longitudinal concentration of CD4^+^ T cells (CAR^−^) in peripheral blood. (B, C, and F) Data are represented as box-and-whisker plots, and bars show minimum and maximum values. (D and E) Bars indicate mean, and errors show ±SEM. For all data, each symbol denotes one mouse, and a Wilcoxon rank sum test was used to calculate significance. ∗p < 0.05, ∗∗p < 0.01. ns, not significant (p > 0.05).

We hypothesized that HIV-specific CAR_4_ T cells will exhibit T cell help to other lymphocytes *in vivo*. To test this, we examined whether CAR_4_ T cells expressing the 4-1BBζ ICD could augment the antiviral function of HIV-specific CAR_8_ T cells after ART removal in HIV-infected humanized mice. We recapitulated the study as described in Figure 4A, but here, mice were allocated into groups that received an HIV-resistant (C34-CXCR4^+^) CAR T cell product consisting of either CAR_8_ T cells alone (2.5 × 10^6^ CAR^+^ cells), a 1:1 mixture of CAR_4_ and CAR_8_ T cells (1.25 × 10^6^ CAR^+^ cells/cell type), or a 1:1 mixture of inactive control CAR_4_ and CAR_8_ T cells (1.25 × 10^6^ CAR^+^ cells/cell type) expressing the CD3Δζ ICD. Of note, CAR_8_ T cells expressed the CD28ζ ICD, which previously demonstrated marginal *in vivo* expansion and protection against HIV infection.^36^ We observed that co-injection of CAR_4_ T cells enhanced the expansion (Figures 6A and 6B) and persistence (Figure 6C) of CD28-costimulated CAR_8_ T cells despite infusing half the dose, whereas alone these cells exhibited limited proliferation relative to control CAR_8_ T cells (Figure S11). In a separate study, CAR_4_ T cells also accelerated the early expansion kinetics of CAR_8_ T cells expressing the 4-1BBζ ICD (Figure S12); this effect was surprising given the remarkable proliferation these cells exhibit on their own after ART cessation.^36^

We next determined whether the co-infusion of CAR_4_ T cells improved virologic outcomes following ART withdrawal. Notably, in this study we observed faster HIV rebound kinetics as viremia was detectable in mice 5 days after ART interruption (Figure 6D) compared to 12 days in our prior study (Figure 4C). Nevertheless, combination therapy with CAR_4_ T cells and CD28-costimulated CAR_8_ T cells reduced rebound viremia (Figure 6E) and effectively limited HIV-induced depletion of CD4^+^ T cells (CAR^−^) (Figure 6F). In contrast, treatment with CAR_8_ T cells alone exhibited a transient reduction in viral load (Figure 6E) but was unable to mitigate CD4^+^ T cell loss relative to control CAR T cell-treated mice (Figure 6F). Taken together, these findings highlight that CAR_4_ T cells provide T cell help by improving the *in vivo* expansion and survival of CAR_8_ T cells, and that combination therapy improves control over HIV pathogenesis.

## Discussion

HIV preferentially infects HIV-specific CD4^+^ T cells, leading to the collapse of CD4^+^ T cell help and impaired antiviral immunity.^1^^,^^2^ Thus, therapeutic approaches that restore CD4^+^ T cell function, such as adoptive T cell therapy, will likely be a critical component of any HIV functional cure or eradication strategy.^60^ In this study, we interrogated the therapeutic potential of HIV-specific CAR_4_ T cells to limit HIV infection *in vitro* and after ART withdrawal in humanized mice. Given how costimulation tunes the functional heterogeneity of CD4^+^ T cells,^61^^,^^62^ we initially characterized how distinct ICDs modulate *in vitro* CAR_4_ T cell functions. We demonstrated that the CD28 receptor family ICDs (ICOS and CD28) induced potent functional profiles compared to the TNFR family ICDs (CD27, OX40, and 4-1BB), exemplified by polyfunctional cytokine responses and direct suppression of *in vitro* virus replication, which rivaled CAR_8_ T cell-mediated control of HIV. However, we did not observe an association between ICDs that conferred optimal *in vitro* function and the ability to mitigate HIV pathogenesis in humanized mice, suggesting that factors driving *in vivo* CAR_4_ T cell-mediated efficacy are not solely predicted *in vitro*.

We identified CAR_4_ T cell expansion as a correlate of *in vivo* antiviral efficacy, supporting observations that CAR T cell persistence is necessary to engender long-term remission of certain B cell malignancies.^63^, ^64^, ^65^ Despite demonstrating limited *in vitro* efficacy, only HIV-resistant (C34-CXCR4^+^) CAR_4_ T cells expressing the 4-1BBζ ICD exhibited profound expansion and survival, and inhibited disease progression after ART removal. The remaining ICDs induced marginal proliferation notwithstanding abundant HIV antigen, in contrast to other studies demonstrating that ICOS,^17^^,^^47^ CD27,^66^ and CD28^67^^,^^68^ costimulatory signals mediate *in vivo* CAR_4_ T cell expansion and tumor eradication. Taken together, these data show that the 4-1BBζ ICD is necessary to potentiate rapid *in vivo* proliferation and long-term survival of CAR_4_ T cells during HIV infection. This finding is critical given that the stability of the latent reservoir in humans^69^^,^^70^ likely necessitates the persistence of CAR T cells for months, years, or decades after infusion to respond to HIV reactivation.^71^

Although the humanized mouse model described herein is well suited to evaluate therapeutic interventions that mitigate active HIV replication and CD4^+^ T cell depletion, these animals eventually develop graft- versus-host disease (GVHD).^72^ This xenoreactivity induces substantial cellular activation and proliferation, resulting in the clinical manifestation of disease within 3–4 weeks after infusion,^73^ which diminishes the time to study the durability of CAR T cell therapy, and limits our ability to recapitulate all aspects of treatment performed in HIV-infected individuals. For instance, the constant immune activation drives supraphysiologic levels of virus replication and precludes the establishment of a latent HIV reservoir, which in humans is comprised of a heterogeneous population of HIV-infected, transcriptionally quiescent memory CD4^+^ T cells.^69^^,^^74^ In contrast, treatments specifically targeting the latent reservoir may instead be accurately reflected in humanized BLT mice, a more complex small-animal model of HIV infection, which supports the generation of a latent reservoir under ART.^75^^,^^76^ Nevertheless, our *in vivo* model enabled us to directly compare the ability of multiple CAR T cell products side by side to mitigate hallmarks of HIV disease progression following ART cessation, which is congruent with the study objectives being investigated by in-human clinical trials (ClinicalTrials.gov: NCT03617198).

Moreover, these findings along with our previous studies indicate that 4-1BB costimulation is essential for both CAR_4_ and CAR_8_ T cells to mitigate *in vivo* HIV pathogenesis after ART cessation,^36^ which contrasts with recent data demonstrating that mesothelin-specific CAR_4_ and CAR_8_ T cells rely on distinct costimulatory signals for optimal function.^47^ However, within this study, we did observe the CD28ζ ICD differentially impacted the efficacy of CAR_4_ and CAR_8_ T cell therapies *in vivo*. For instance, CD28-costimulated CAR_4_ T cells failed to proliferate and mount effective immunity against viral recrudescence compared to mice treated with 4-1BB-costimulated CAR_4_ T cells (Figures 4D–4F). In contrast, the data described herein support our previous work,^36^ showing that CAR_8_ T cells expressing the CD28ζ ICD were capable of reducing viremia and delaying CD4^+^ T cell (CAR^−^) depletion to the same extent as 4-1BB-costimulated CAR_8_ T cells early after ART cessation. These findings reinforce the notion that costimulatory signals that engender favorable CAR T cell responses are likely both disease- and cell type-specific.^22^

In addition to CAR_4_ T cells exhibiting direct control of *in vitro* HIV replication, we reasoned that they could also restore CD4^+^ T cell help that is lost as a consequence of viral infection. To this end, we demonstrated that co-injection of CAR_4_ T cells enhanced the *in vivo* proliferation kinetics and survival of CD28-costimulated CAR_8_ T cells, concomitant with reduced HIV replication. Moreover, we have shown that *in vitro* activation of 4-1BB-costimulated CAR_4_ T cells upregulates IL-2 and IL-21, which are cytokines that augment the function of CD8^+^ T cells during viral infection,^77^, ^78^, ^79^ and thus may have contributed to the sustained CAR_8_ T cell response that we observed *in vivo*. Furthermore, it is reasonable to think that CAR_4_ T cell help will benefit endogenous, HIV-specific immunity. For example, previous studies have demonstrated that HIV-specific cytolytic CD4^+^ and CD8^+^ T cells exhibit strong cooperativity to suppress infection *in vitro*,^80^ and the addition of vaccine-primed HIV-specific CD4^+^ T cells reinvigorate the proliferative capacity of CD8^+^ T cells isolated from chronic infection.^81^ Finally, since our CAR_4_ T cells still express a functional TCR, it is likely that a portion of the infused CAR_4_ T cells maintain specificity to other pathogens and are able to confer help to immune responses toward other infections, including Epstein-Barr virus (EBV), flu, and cytomegalovirus (CMV). Interestingly, it was reported that repeated TCR stimulation of CAR T cells results in exhaustion of CAR_8_, but not CAR_4_, T cells,^82^ suggesting that CAR_4_ T cell-mediated help could be durable.

The ability of HIV-specific CAR_4_ T cells to control HIV replication, as well as enable the function of other immune cells, highlights the therapeutic potential of this T cell population. However, we expect that next-generation CAR_4_ T cells will need to be infused into HIV-infected individuals as part of a defined formulation with CAR_8_ T cells to achieve a synergistic antiviral effect, similar to the cooperativity between these T cells during cancer treatment.^65^^,^^83^ Moreover, the potential of CAR T cell therapy alone to engender positive clinical outcomes may only occur after ART cessation when sufficient viral antigen is present to induce CAR T cell activation, as opposed to eliminating the latent reservoir during ART when antigen is restricted. In this way, CAR T cell therapy may resolve post-peak rebound viremia and contribute to an ART-free remission of HIV. Collectively, the findings described herein provide insight regarding the potential of engineered CD4^+^ T cells to both augment and restore the function of HIV-specific CD4^+^ T cells that is typically lost by virus-associated depletion, which could serve to reinvigorate broad and enduring immune responses that enable immune control over HIV.

## Materials and Methods

### Ethics Statement

Humanized mouse experiments performed at the University of Pennsylvania were approved by the University of Pennsylvania Institutional Animal Care and Use Committee (IACUC) under approval protocol 805606. All animal studies were carried out in accordance with recommendations in the *Guide for the Care and Use of Laboratory Animals* of the National Institutes of Health. Peripheral blood mononuclear cells and purified adult human CD8^+^ and CD4^+^ T cells were obtained by the University of Pennsylvania Human Immunology Core/Center for AIDS Research (CFAR) Immunology Core from de-identified healthy donors.

### Flow Cytometry

Surface staining was performed in 1× PBS containing 2% fetal calf serum and 2 mM EDTA using anti-human antibodies from BioLegend: CD45 (2D1), CD3 (OKT3), CD4 (OKT4), CD8 (RPA-T8), TIGIT (VSTM3), PD-1 (EH12.2H7), CD107a (H4A3), and CD271/NGFR (ME20.4). Live cells were discriminated by staining with fixable viability dye eFluor 780 (eBioscience). Intracellular proteins were stained for with a cell fixation and cell permeabilization kit (Invitrogen) according to the manufacturer’s instructions using antibodies from the following sources: BD Biosciences: TNF (Mab11), IFN-γ (4S.B3), GM-CSF (BVD2-21C11), MIP-1β (D21-1351), IL-21 (3A3-N2.1), GzmB (GB11), and active caspase-3 (C92605); BioLegend: IL-2 (MQH-17H12), IL-17A (BL168), IL-4 (8D4-8), IL-13 (JES10), IL-22 (2412A41), perforin (B-D48), and GzmA (CB9); eBioscience: GzmM (4B2G4); and Beckman Coulter: HIV-1 core antigen (KC57). Flow cytometry data were acquired on a BD LSRFortessa and analyzed using FlowJo software version 10.5.3 (Tree Star).

### HIV Viral Load Quantitation

Viral RNA was isolated from plasma (40 μL) using the QiaAmp viral RNA mini kit (QIAGEN). Viral loads were determined using quantitative RT-PCR using the QuantiFast SYBR Green RT-PCR kit (QIAGEN) as previously described.^84^ The limit of quantification for this assay is 1.81 log RNA copies/mL plasma.

### Plasmid Construction

The amino acid sequences for the HIV-specific CD4-based CAR constructs contained the following intracellular domains: CD3-ζ, 4-1BB/CD3-ζ, CD28/CD3-ζ, CD27/CD3-ζ, OX40/CD3-ζ, and ICOS/CD3-ζ, as described elsewhere.^36^ In this study, each CAR was amplified from its original plasmid with the primer 5′-CACGTCCTAGGATGGCCTTACCAGTG-3′ and 5′-GTGGTCGACTTATGCGCTCCTGCTGAAC-3′ and cloned into pTRPE plasmid using the AvrII and SalI restriction enzyme sites. In this orientation, the CAR sequence is downstream of GFP, and a T2A sequence intervenes to permit expression of both proteins. The amino acid sequence for the C34-CXCR4 construct is described elsewhere.^35^ We introduced a single Asp mutation (D97N), which has been shown to impair SDF-1 binding^85^ and limit CXCR4 internalization (G.J. Leslie, M. Richardson, J.L.R., and J.A. Hoxie, personal communication). C34-CXCR4 (D97N) was cloned upstream of T2A and NGFR^86^ sequences within the pTRPE backbone.

### Lentivirus Production and Transfection

Lentivirus particles were generated using expression vectors encoding vesicular stomatitis virus (VSV) glycoprotein (pTRPE pVSVg-g), HIV Rev (pTRPE.Rev), and HIV Gag and Pol (pTRPE g/p). The plasmids were synthesized by DNA 2.0 or ATUM (Newark, CA, USA) and transfected into HEK293T cells with pTRPE transfer vectors described above using Lipofectamine 2000 (Life Technologies) as previously described.^31^ Transfected HEK293T cell supernatant was collected 24 and 48 h after transfection, filtered through a sterile 0.45-μm nylon syringe-driven filter, and concentrated by ultracentrifugation at 25,000 rpm for 2.5 h at 4°C. Supernatant was aspirated and the virus pellet was resuspended in 800-μL total volume and stored at −80°C.

### Cell Culture and Selection

To manufacture CAR T cells, CD4^+^ and CD8^+^ T cells from healthy adult human donors were purified by negative selection using RosetteSep human CD4^+^ or CD8^+^ enrichment cocktails (STEMCELL Technologies) according to the manufacturer’s instructions. All cells were cultured at 10^6^ cells/mL in complete RPMI (expansion medium): RPMI 1640, 2 mM GlutaMax, 25 mM HEPES, and 1% penicillin-streptomycin from Life Technologies, and 10% fetal calf serum (Seradigm). T cell expansion medium was supplemented with 10 ng/mL human IL-7 (R&D Systems) and 5 ng/mL human IL-15 (BioLegend) or 100 U/mL human IL-2 (Clinigen). T cells were stimulated with anti-CD3/CD28 Dynabeads (Life Technologies) at a 3:1 bead-to-cell ratio at 37°C, 5% CO_2_ and 95% humidity incubation conditions. Roughly 18 h after stimulation, half of the medium was removed and replaced with 200–300 μL of concentrated lentivirus supernatant for CAR transduction. On day 5, the Dynabeads were removed from cell culture by magnetic separation. Expansion medium was changed every other day throughout cell culture spanning 8–10 days, or as necessary to adjust cell concentration to 0.5 × 10^6^ cells/mL. For *in vivo* studies, on day 7 after activation, C34-CXCR4-transduced T cells were positively selected using CD271/NGFR microbeads (Miltenyi Biotec) according the manufacturer’s protocol. Following selection, C34-CXCR4^+^ T cells were placed in culture with expansion medium for one more day at the adjusted cell concentration prior to infusion into humanized mice.

### *In Vitro* HIV Suppression Assay

Two days after removing anti-CD3/CD28 Dynabeads, activated CD4^+^ T cells were infected with CCR5-tropic HIV_BAL_ at a multiplicity of infection (MOI) of 1. 24 h later, HIV-challenged CD4^+^ T cells were washed with complete RPMI and mixed with the indicated type of HIV-specific CAR T cell, or untransduced (UTD) T cell at E:T ratios of 1:25, 1:50, 1:100, and 1:200. Cell mixtures were plated in duplicate and the spread of virus replication was assessed by flow cytometry by sampling 100 μL per well for intracellular staining of HIV-1 core antigen (HIV_GAG_) at 2, 4, 6, and 8 days after co-culture. Fresh complete RPMI was added to all wells after staining.

### CD107a Degranulation and Intracellular Staining Assay

The functionality of CAR T cells was measured *in vitro* after stimulating 2 × 10^5^ CAR T cells or UTD T cells with 2 × 10^5^ wild-type K562s (K.WT) or K562 cells expressing HIV_YU2_ GP160 (K.Env). Anti-CD107a antibody was added at the start of stimulation followed by the addition of 1× brefeldin A and monensin solution (BioLegend) 1 h later. Cells were incubated for a total of 6 h under incubation conditions. Detection of cytokines and cytolytic proteins was assessed by intracellular staining with antibodies specific for human TNF, MIP-1β, IFN-γ, IL-2, GM-CSF, perforin, GzmB, GzmA, GzmM, IL-17A, IL-4, IL-13, IL-21, and IL-22. The percentage of cytokine-positive CAR T cells was calculated by subtracting background production after stimulation with K.WT cells.

### HIV-Infected Cell Elimination Assay

This HIV-infected cell elimination assay is based on a previously described protocol.^54^ Briefly, HIV-infected CD4^+^ T cells were prepared as detailed above. When roughly 30% of total cells stained positive for intracellular HIV_GAG_, the cells were labeled with CellTrace Violet (CTV; Thermo Fisher Scientific) to distinguish target cells from effector cells. CAR or UTD T cells were cultured with CTV^+^ target cells at various E:T ratios. After 24 h, target cells were analyzed for the reduction of HIV_GAG_^+^ cells and the induction of active caspase-3 by intracellular staining and flow cytometry. Elimination of HIV-infected cells was calculated by quantitation of live (fixable viability dye eFluor780-negative) CTV^+^CAR^−^HIV_GAG_^+^ cells. The percentage of residual HIV-infected target cells were calculated by dividing the percentage of live CTV^+^CAR^−^HIV_GAG_^+^ target cells at the indicated E:T ratio by the percentage of CTV^+^CAR^−^HIV_GAG_^+^ target cells at an E:T ratio of 0.

### Humanized Mice Experiments

NOD (non-obese diabetic)/SCID (severe combined immunodeficiency)/IL-2Rγ^−/−^ (NSG) mice (The Jackson Laboratory) were housed in a pathogen-free facility at the University of Pennsylvania. Mice were maintained in microisolator cages and fed autoclaved food and water. Humanized mice were generated essentially as previously described.^36^ NSG mice were infused with 5 × 10^6^ healthy donor PBMCs depleted of CD8^+^ T cells using CD8 microbeads (Miltenyi Biotec) following the manufacturer’s protocol. Two weeks later mice initiated daily ART consisting of 1 mg/kg EFdA (4′-ethynyl-2-fluoro-2′-deoxyadenosine, LeadGen Labs). After 1 day, mice were infused with 10^6^ autologous HIV_GAG_^+^CD4^+^ T cells and mixed with 9 × 10^6^ uninfected CD4^+^ T cells. Prior to infusion, CD4^+^ T cells were *in vitro* infected with HIV_BAL_ (MOI of 1) as described above and expanded in culture for 4 more days, where during the last 2 days 1 μM EFdA supplemented the expansion medium. Mice were bled 1 or 2 days after the infusion of HIV-infected cells and then allocated into groups (n = 6–8) based on the concentration of peripheral blood CD4^+^ T cells. Three days after the infusion of HIV-infected cells, mice were infused with HIV-resistant (>90% C34-CXCR4^+^) CAR T cells as described above and ART was interrupted. For the study described in Figure 4, each group of humanized mice received an infusion of 2.5 × 10^6^ CAR_4_ T cells expressing one of the six ICDs, or inactive control CAR_4_ T cells expressing a truncated CD3ζ ICD. For the study described in Figure 6, mouse groups received an infusion product consisting of either (CD28ζ) CAR_8_ T cells (2.5 × 10^6^ CAR^+^ cells), a 1:1 ratio of CAR_4_ (4-1BBζ) and CAR_8_ (CD28ζ) T cells (1.25 × 10^6^ CAR^+^ cells/cell type), or 1:1 ratio of inactive control CAR_4_ and CAR_8_ T cells (1.25 × 10^6^ CAR^+^ cells/cell type) expressing the CD3Δζ ICD. In another study shown in Figure S12, we recapitulated this experimental design, but the CAR_8_ T cells expressed the 4-1BBζ ICD instead of the CD28ζ ICD. For all studies the mice were bled weekly via retro-orbital puncture following the CD4^+^ T cell engraftment bleed until the study endpoint.

### Statistical Analysis

All statistical analysis was performed using GraphPad Prism version 7 (GraphPad, San Diego, CA, USA). A comparison of means from unmatched samples was performed using the non-parametric Wilcoxon rank sum test or Kruskal-Wallis test followed by Dunn’s test for multiple comparisons. Comparison of means from matched samples was performed using paired Student’s t test or Wilcoxon a matched-pairs signed-rank test. Bivariate correlations were performed using Spearman’s rank correlation. Area under the curve calculations were performed using either cell concentration normalized to 1 μL of blood or frequency of HIV-infected cells.

## Author Contributions

C.R.M., K.G., R.S.L., D.L.D., J.P.M., X.S., and J.A.G. contributed to the acquisition and analysis of data; C.R.M. and J.L.R. conceived and designed the project, contributed to the interpretation of the data, and drafted the manuscript.

## Conflicts of Interest

R.S.L. and J.L.R. have filed a patent describing the construction of these HIV-specific CARs. J.L.R. co-founded Tmunity Therapeutics that has the rights to license the technology described herein. J.L.R. holds an equity interest in Tmunity. The remaining authors declare no competing interests.

## Supplemental Information

Document S1. Figures S1–S12 and Table S1

Document S2. Article plus Supplemental Information
