# Supplementary material for: HIV-Resistant and HIV-Specific CAR-Modified CD4+ T Cells Mitigate HIV Disease Progression and Confer CD4+ T Cell Help In Vivo
Source: Mol Ther. 2020 May 15;28(7):1585–99. doi: 10.1016/j.ymthe.2020.05.012 (PMC7335752; doi:10.1016/j.ymthe.2020.05.012)
Supplement: Document S1. Figures S1–S12 and Table S1 [file mmc1.pdf]

YMTHE, Volume *28*

Supplemental Information

HIV-Resistant and HIV-Specific CAR-Modified CD4$^+$ T Cells Mitigate HIV Disease Progression and Confer CD4$^+$ T Cell Help *In Vivo*

Colby R. Maldini, Kevin Gayout, Rachel S. Leibman, Derrick L. Dopkin, Joshua P. Mills, Xiaochuan Shan, Joshua A. Glover, and James L. Riley

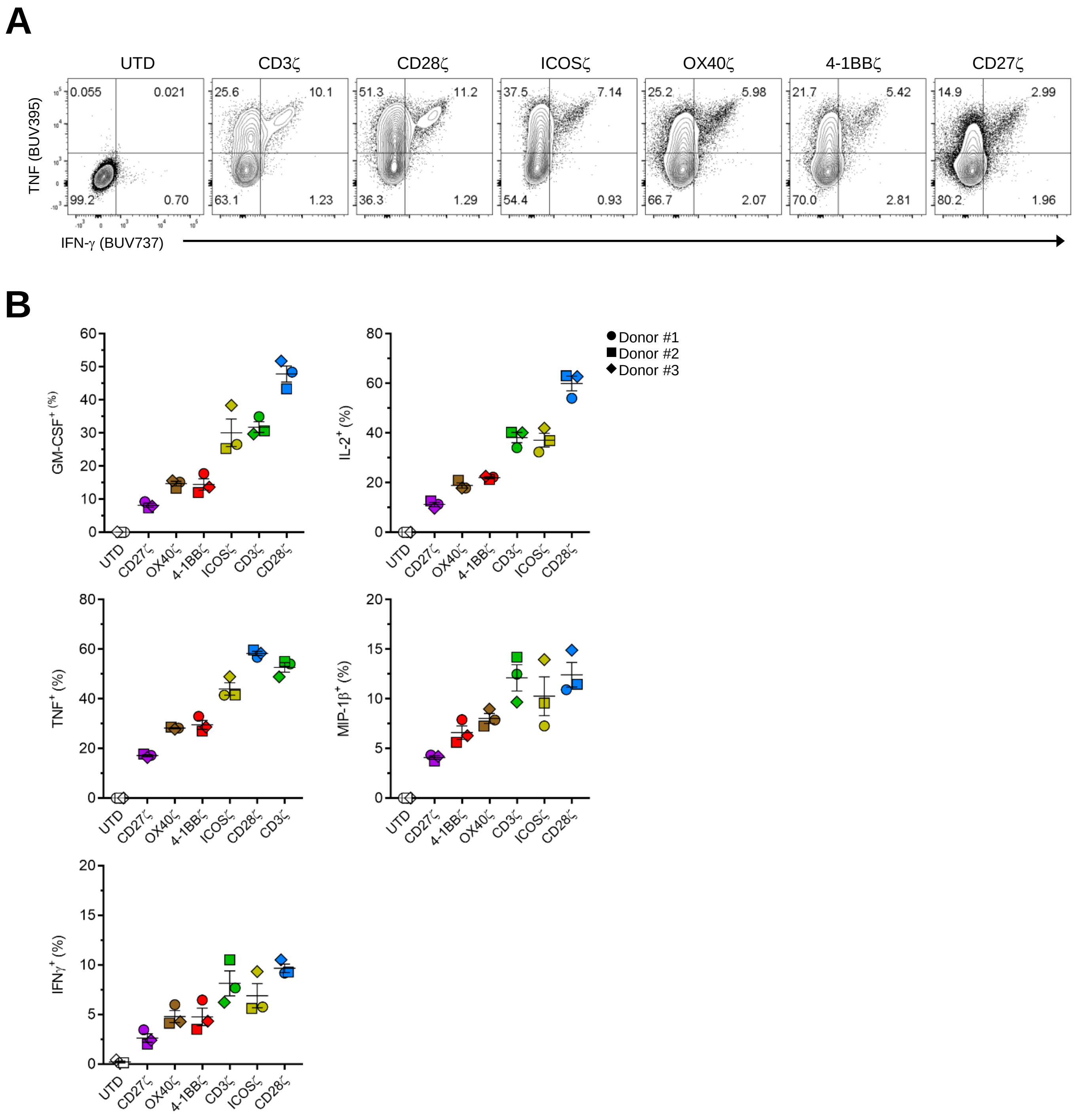

Figure S1. Distinct HIV-specific CAR_4 T cell types differentially express cytokines after *in vitro* antigen-specific stimulation. **(A)** FACS plots indicating the frequency of cytokine expressing HIV-specific CAR_4 T cells after *in vitro* stimulation with K.Env cells 10 days after activation with αCD3/CD28 Dynabeads. Data are representative of 3 donors. **(B)** Summary data indicating the frequency of cytokine expressing CAR_4 T cells. Each symbol represents a distinct donor. The percentage of cytokine expressing CAR_4 T cells was calculated by subtracting background production after stimulation with wild-type K562 cells. Lines indicate mean and error bars show ± SEM.

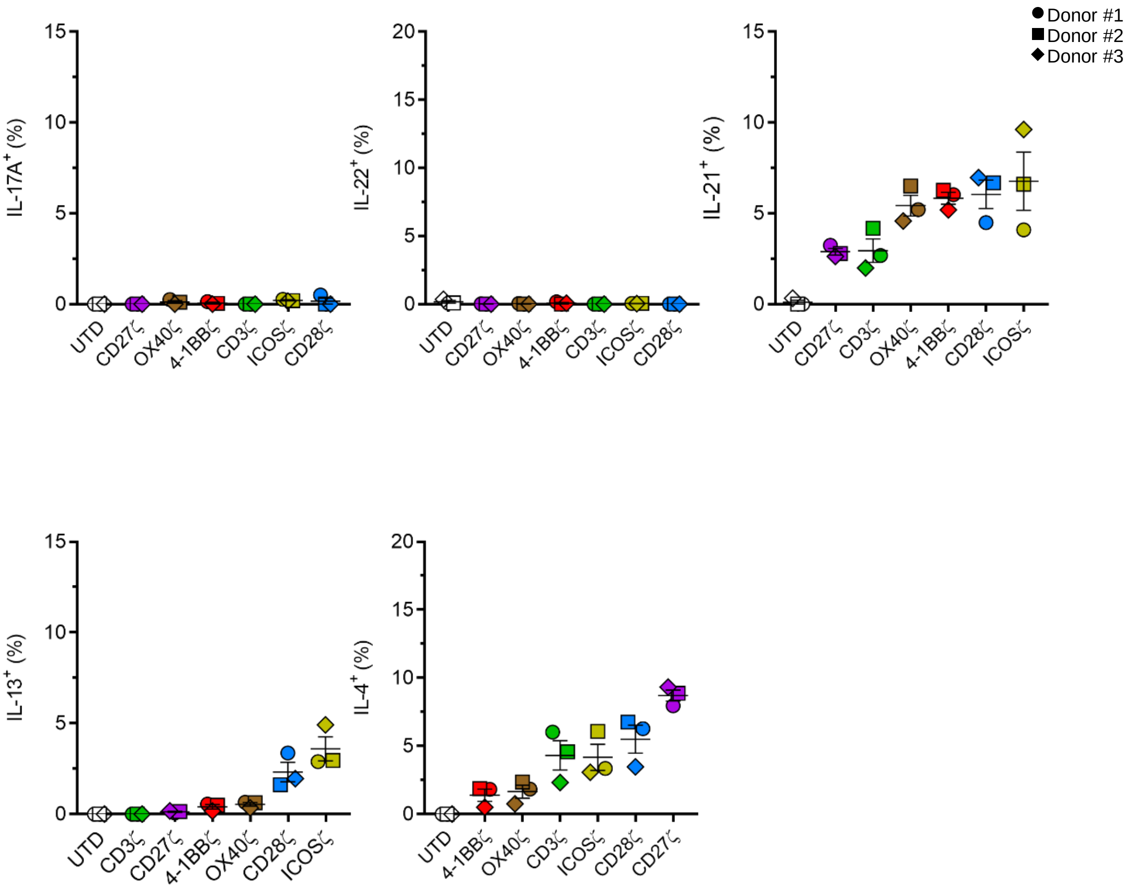

Figure S2. Distinct HIV-specific CAR_4 T cell types express low levels of Th17- and Th2-associated cytokines. Summary data indicating the frequency of cytokine expressing CAR_4 T cells after *in vitro* stimulation with K.Env 10 days after activation with αCD3/CD28 Dynabeads. Each symbol represents a distinct donor. The percentage of cytokine expressing CAR_4 T cells was calculated by subtracting background production after stimulation with wild-type K562 cells. Lines indicate mean and error bars show ± SEM.

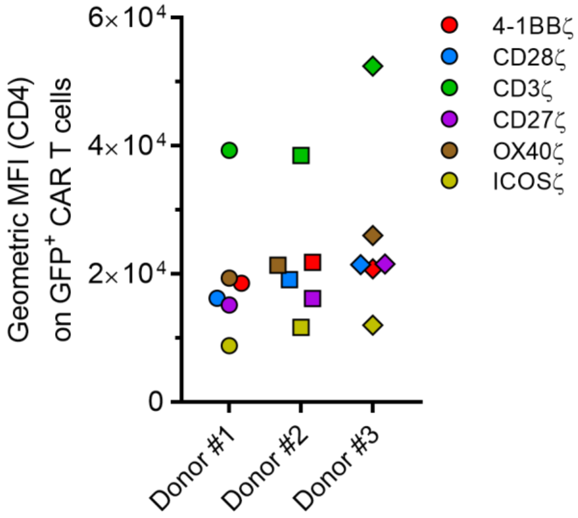

Figure S3. CD4-based CAR containing the CD3ζ intracellular domain exhibits the greatest surface expression on CD4⁺ T cells. Summary data indicates the geometric mean fluorescence intensity (MFI) determined by flow cytometry of CD4 on GFP⁺ CAR_4 T cells 10 days after activation with αCD3/CD28 Dynabeads.

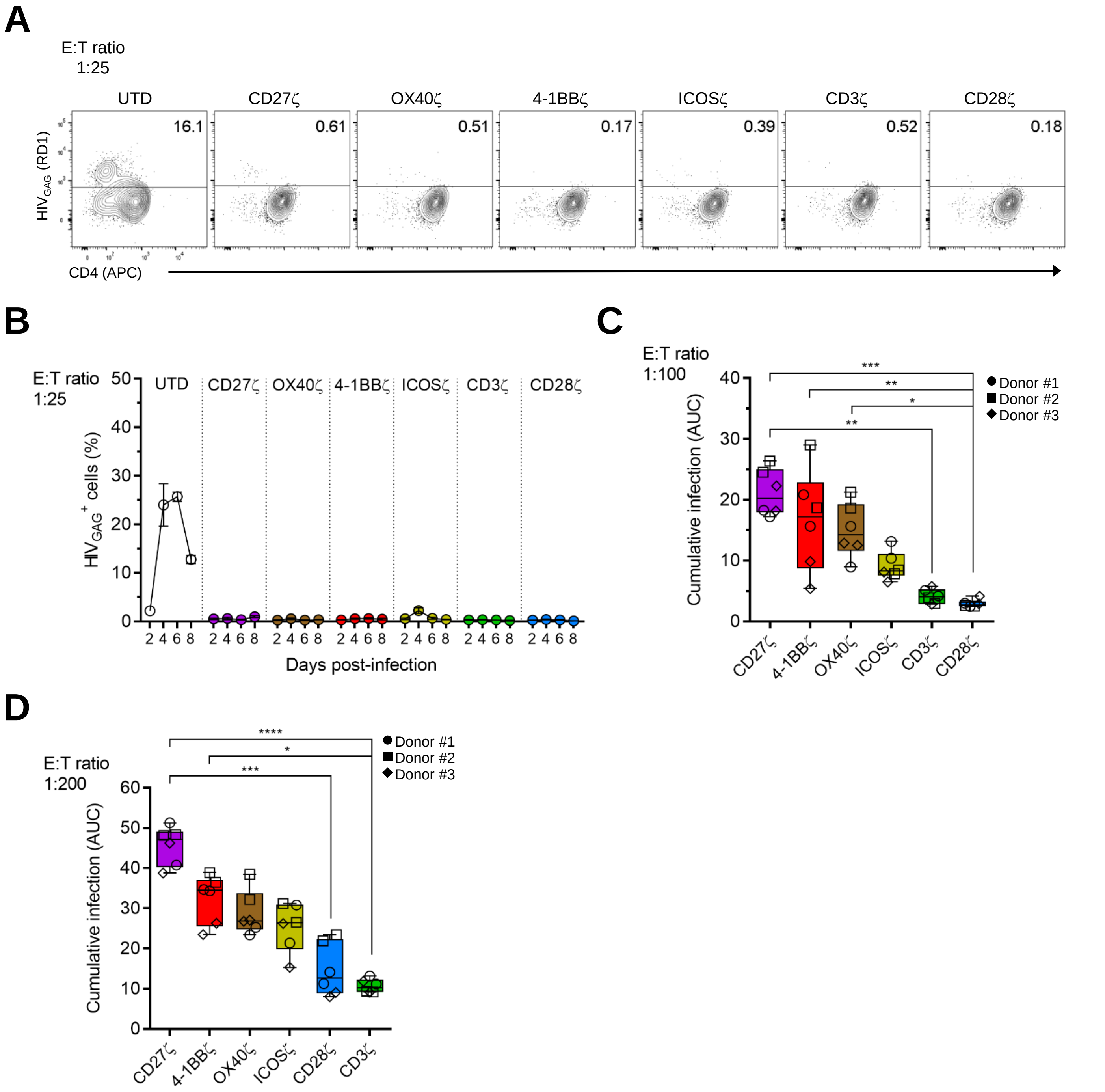

Figure S4. Intracellular domains modulate CAR_4 T cell-mediated control of *in vitro* HIV replication. Each HIV-specific CAR_4 T cell type and UTD_4 T cells were mixed separately with HIV_{BAL}-challenged $CD4^+$ T cells at the indicated effector-to-target (E:T) ratios, and the level of virus spread was monitored by intracellular staining and flow cytometry for HIV_{GAG} antigen on 2, 4, 6 and 8 days after co-culture. **(A)** FACS plots indicating the frequency of HIV_{GAG}^+ cells (CAR^-) 8 days after co-culture with CAR_4 or UTD_4 T cells at the 1:25 E:T ratio. **(B)** Summary of the frequency of HIV_{GAG}^+ cells at 2, 4, 6 and 8 days after co-culture at 1:25 E:T ratio. Symbols represents the average of 3 distinct donors in duplicate and error bars show ± SEM. **(C)** Cumulative infection calculated by area under the curve from the frequency of HIV_{GAG}^+ cells at 2, 4, 6 and 8 days after co-culture at 1:100 and **(D)** 1:200 E:T ratios. Data are represented as box and whisker plots and bars show min and max values. Symbols indicate unique donors performed in duplicate. Kruskal-Wallis test and Dunn's multiple comparison test was used to determine significance (**P<0.05, **P<0.01, ***P<0.001, ****P<0.0001*).

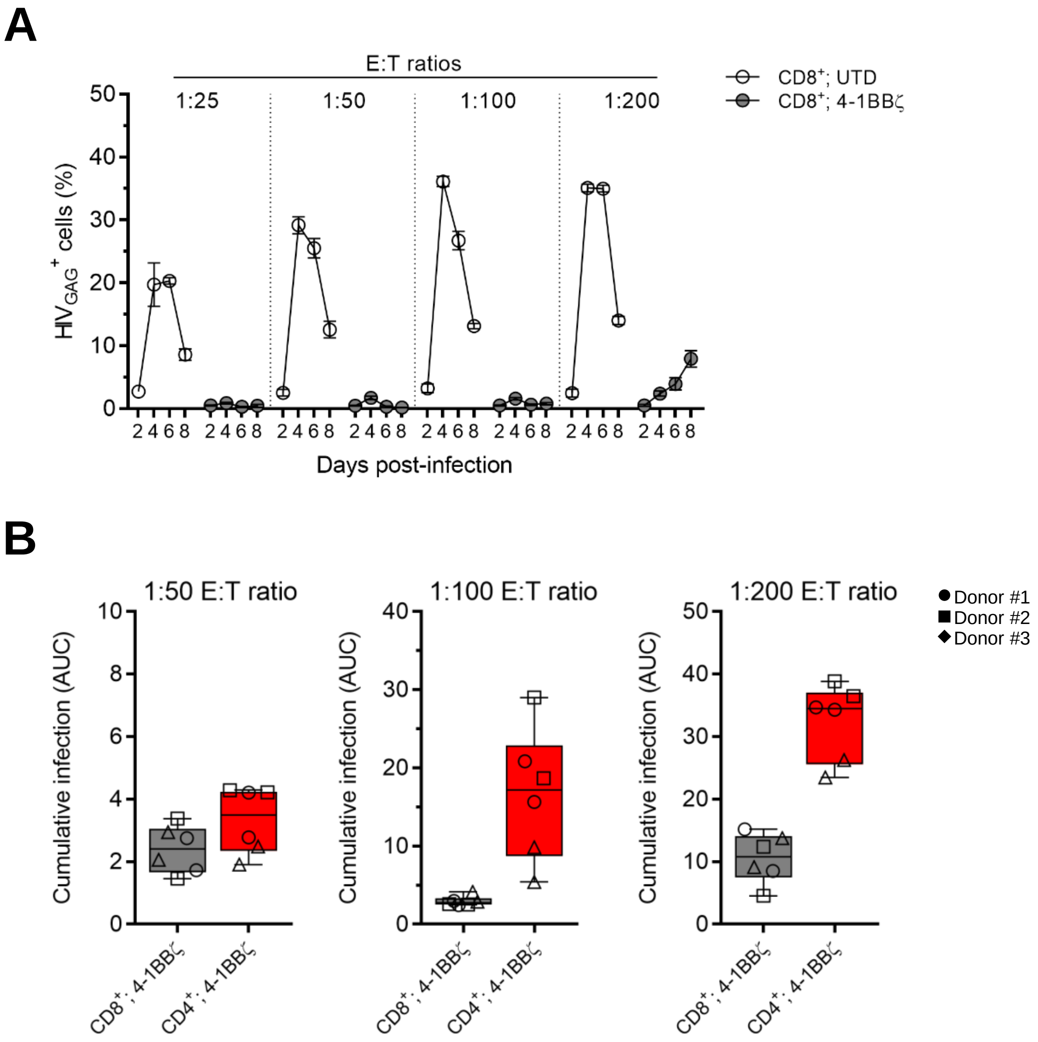

Figure S5. HIV-specific CAR_8 T cells expressing the 4-1BBζ ICD durably suppress *in vitro* virus replication. UTD and HIV-specific CAR_4 and CAR_8 T cells expressing the 4-1BBζ intracellular domain were mixed separately with HIV_{BAL}-challenged $CD4^+$ T cells at the indicated effector-to-target (E:T) ratios, and the level of virus spread was monitored by intracellular staining and flow cytometry for HIV_{GAG} antigen on 2, 4, 6 and 8 days after co-culture. **(A)** Summary of the frequency of HIV_{GAG}^+ (CAR^-) cells at 2, 4, 6 and 8 days after co-culture. Symbols represents the average of 3 donors in duplicate and error bars show ± SEM. **(B)** Cumulative infection calculated by area under the curve from the frequency of HIV_{GAG}^+ cells at 2, 4, 6 and 8 days after co-culture. Data are represented as box and whisker plots and bars show min and max values. Symbols indicate unique donors performed in duplicate.

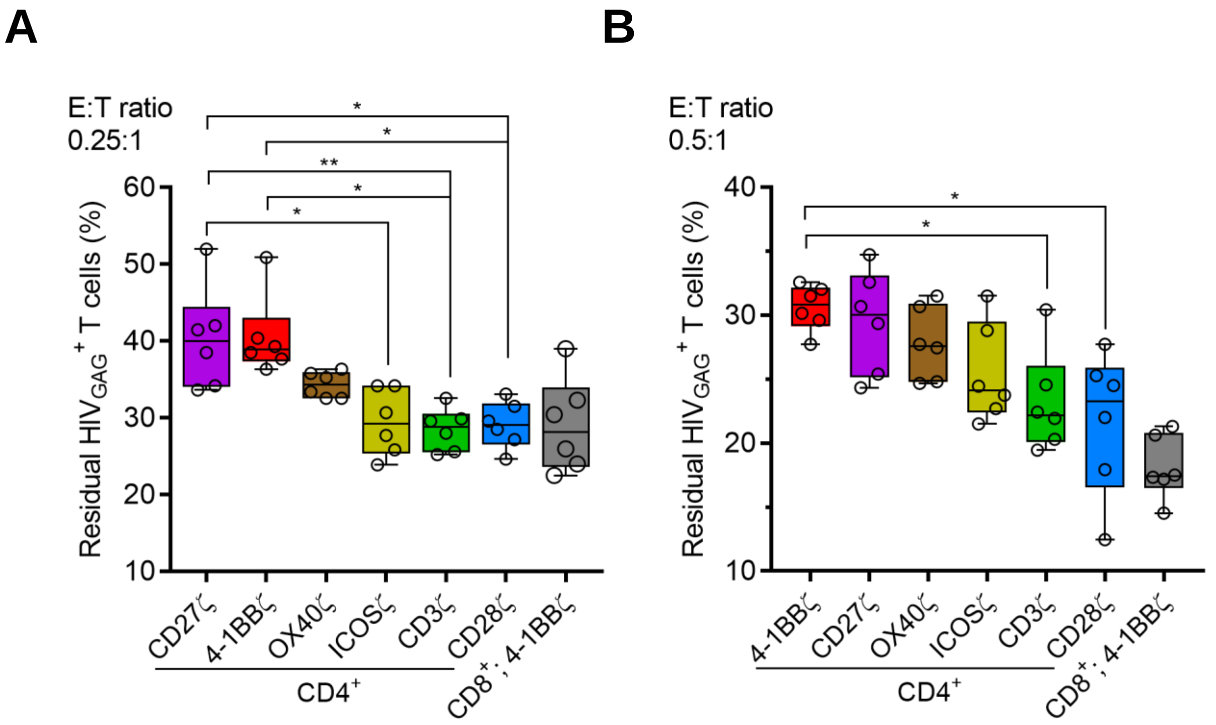

Figure S6. HIV-specific CAR_4 T cells exhibit *in vitro* cytotoxic activity. CellTrace Violet labelled, HIV_{BAL}-infected $CD4^+$ T cells (30% HIV_{GAG}^+) were cultured with UTD or HIV-specific CAR T cells at the indicated E:T ratios. Frequency of HIV_{GAG}^+ cells (live CAR^- $CD8^-$ T cells) was assessed by intracellular staining and flow cytometry for HIV_{GAG} antigen 24 hours later. **(A)** Summary data indicates the frequency of residual HIV_{GAG}^+ cells that exist after co-culture with CAR T cells at the 0.25:1 and **(B)** 0.5:1 E:T ratio. Data are represented as box and whisker plots and bars show min and max values. Symbols indicate 3 donors performed in duplicate. Kruskal-Wallis test and Dunn's multiple comparison test was used to determine significance (*$P<0.05$, **$P<0.01$).

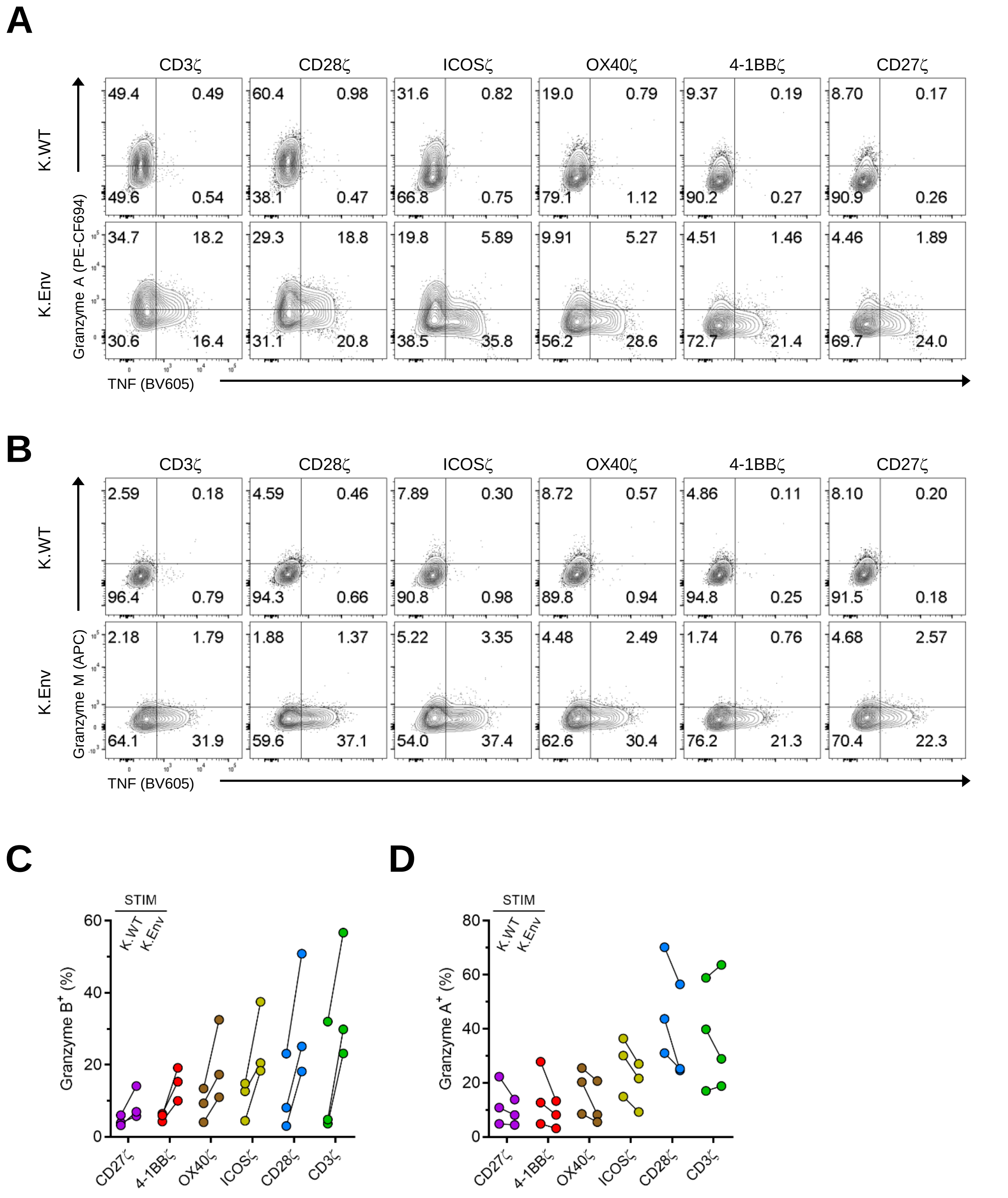

Figure S7. HIV-specific CAR_4 T cells differentially express Granzymes after *in vitro* stimulation. After 10 days of culture, each CAR_4 T cell type was *in vitro* stimulated with K.Env or wild-type K562 cells (K.WT) and the intracellular expression levels of Granzyme B, A and M were assessed. **(A)** FACS plots show the frequency of Granzyme A and **(B)** Granzyme M expression in CAR_4 T cells. Data are representative of 3 distinct donors. **(C)** Summary data showing the change in Granzyme B and **(D)** Granzyme A levels after *in vitro* stimulation. Each symbol represents one donor.

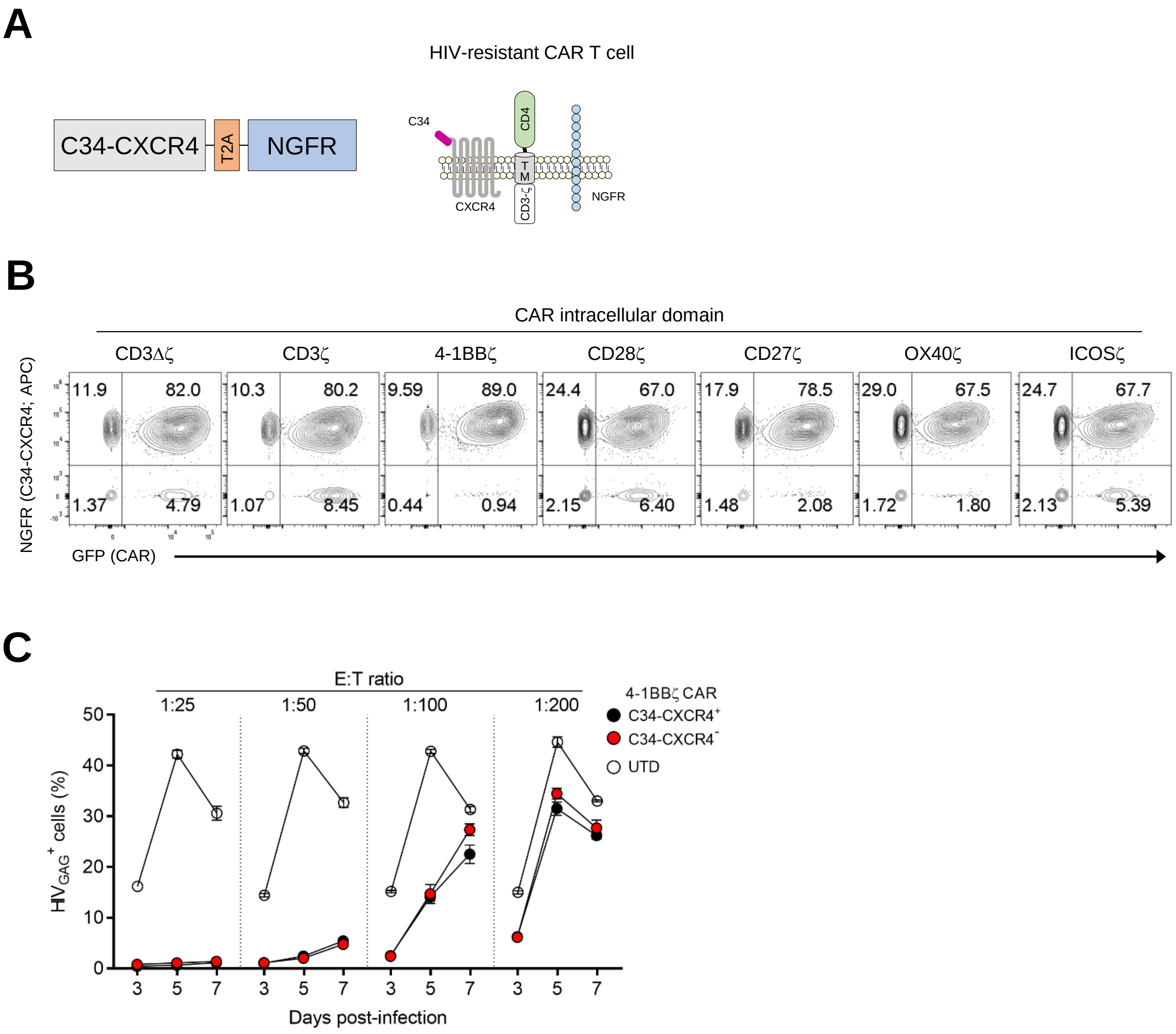

Figure S8. Manufacturing and *in vitro* functional evaluation of HIV-resistant CAR T cells. HIV-specific CAR_4 T cells were co-transduced with lentivirus encoding the HIV fusion inhibitor C34-CXCR4 linked to NGFR by an intervening T2A sequence. Seven days after activation with αCD3/CD28 Dynabeads, T cells were positively selected for NGFR expression using αNGFR antibody coated magnetic beads as described in Materials and Methods. **(A)** Schematic of the C34-CXCR4 construct used to confer HIV-resistance to CAR_4 T cells. **(B)** FACS plots indicate the purity of $NGFR^+$ CAR_4 T cells following magnetic bead selection prior to infusion into HIV-infected humanized mice. **(C)** HIV suppression assay as described in Materials and Methods. Viral outgrowth kinetics when HIV-infected $CD4^+$ T cells are co-cultured with either C34-$CXCR4^+$ or C34-$CXCR4^-$ CAR T cells expressing the 4-1BBζ intracellular domain or UTD. Symbols indicate mean and error bars show ± SEM.

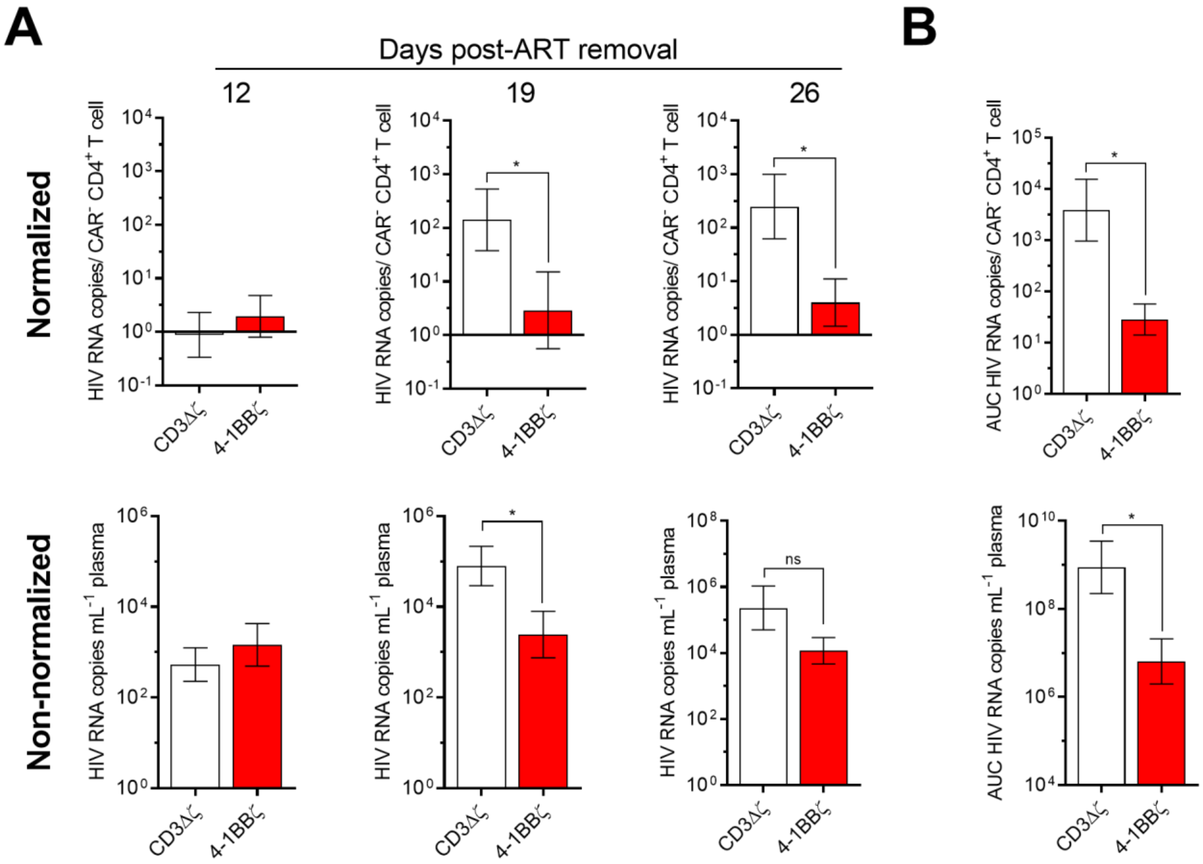

Figure S9. Plasma viral load comparison of normalized and non-normalized values. (A) Viral loads of 4-1BBζ CAR_4 T cell-treated mice compared to control CD3Δζ CAR_4 T cell-treated mice (see also Figure 4). HIV RNA copies mL^{-1} plasma are either normalized to contemporaneous peripheral $CD4^+$ T cell (CAR^-) concentration or non-normalized (i.e raw concentration). **(B)** Cumulative viral load calculated by area under the curve from 5, 12, 19 and 26 days post-ART removal. For all data, bars indicate mean and error bars show ± SEM. Significance was calculated using Wilcoxon rank sum text (**P*<0.05)

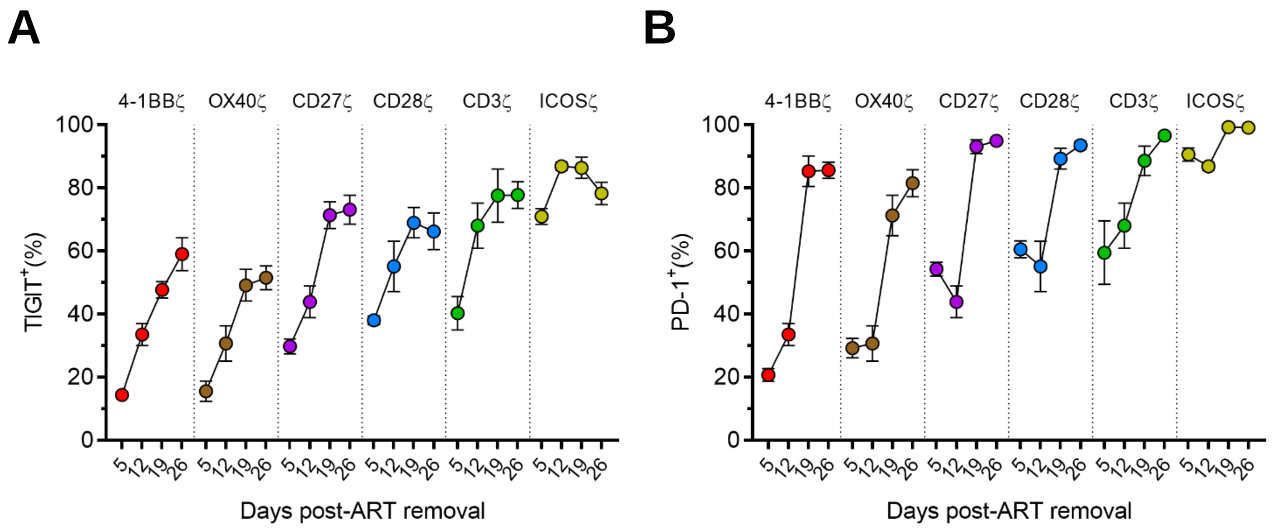

Figure S10. HIV-specific CAR$_4$ T cells expressing TNFR family intracellular domains exhibit lower inhibitory receptor expression post-ART. (A) Longitudinal expression of TIGIT and **(B)** PD-1 on peripheral blood HIV-specific CAR$_4$ T cells post-ART removal in HIV-infected humanized mice. Symbols indicate mean and error bars show ± SEM.

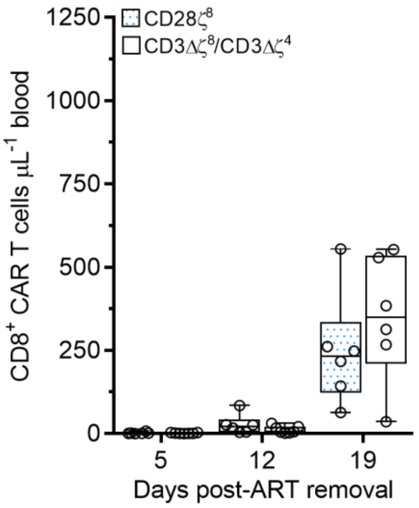

Figure S11. HIV-specific CD28-costimulated CAR_8 T cells fail to expand post-ART removal *in vivo*. Longitudinal concentration of HIV-specific CAR_8 T cells expressing either the CD28ζ or CD3Δζ ICD in peripheral blood following ART withdrawal in HIV-infected humanized mice. Data are represented as box and whisker plots and bars show min and max values. Each symbol denotes one mouse.

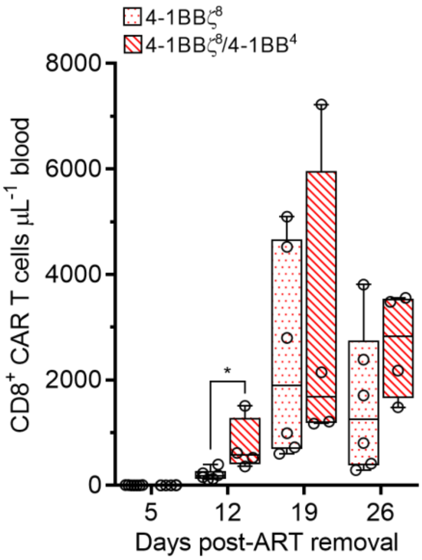

Figure S12. Co-injection of HIV-specific CAR_4 T cells improve early expansion kinetics of 4-1BB-costimulated CAR_8 T cells post-ART removal. NSG mice were infused with CD8-depleted PBMCs from a healthy human donor. Two weeks later, mice initiated daily ART for one week and were infused with autologous, *in vitro* HIV_{BAL}-infected $CD4^+$ T cells. Mice were allocated into 3 groups (n=4-6) based on $CD4^+$ T cell engraftment, and then each mouse received either HIV-resistant (C34-$CXCR4^+$) CAR_8 (4-1BBζ) T cells ($2.5x10^6$ CAR^+ cells), 1:1 ratio of CAR_4 (4-1BBζ) and CAR_8 (4-1BBζ) T cells ($1.25x10^6$ CAR^+/cell type), or 1:1 ratio of inactive control CAR_4 and CAR_8 T cells ($1.25x10^6$ CAR^+/cell type) expressing the CD3Δζ ICD followed by ART interruption. Longitudinal concentration of peripheral CAR_8 T cells after ART interruption. Data are represented as box and whisker plots and bars show min and max values. Each symbol denotes one mouse and Wilcoxon rank sum test was used to calculate significance (*$P<0.05$).

Table S1. Statistical comparison of normalized plasma viral loads (*P* value)

		CAR$_4$ T cell populations					
**Days post-ART removal**	**CAR$_4$**	**CD3Δζ**	**CD3ζ**	**CD28ζ**	**ICOSζ**	**CD27ζ**	**OX40ζ**
19	4-1BBζ	*0.014	*0.014	*0.014	0.234	0.101	0.035
26	4-1BBζ	*0.035	*0.035	0.467	0.628	0.628	0.180

Significance was calculated using Wilcoxon rank sum test (*P<0.5)
Statistical comparison of 4-1BBζ CAR$_4$ T cell-treated group and other indicated CAR$_4$ types (see also Figure 4)
