# Supplementary material for: HIV-Resistant and HIV-Specific CAR-Modified CD4+ T Cells Mitigate HIV Disease Progression and Confer CD4+ T Cell Help In Vivo
Source: Mol Ther. 2020 May 15;28(7):1585–99. doi: 10.1016/j.ymthe.2020.05.012 (PMC7335752; doi:10.1016/j.ymthe.2020.05.012)
Supplement: Document S2. Article plus Supplemental Information [file mmc2.pdf]

Molecular Therapy
Original Article

HIV-Resistant and HIV-Specific CAR-Modified CD4+ T Cells Mitigate HIV Disease Progression and Confer CD4+ T Cell Help *In Vivo*

Colby R. Maldini,[1] Kevin Gayout,[1] Rachel S. Leibman,[1] Derrick L. Dopkin,[2] Joshua P. Mills,[1] Xiaochuan Shan,[2] Joshua A. Glover,[2] and James L. Riley[1]

[1]Department of Microbiology, Center for Cellular Immunotherapies, Perelman School of Medicine, University of Pennsylvania, Philadelphia, PA 19104, USA; [2]Deparment of Pathology & Laboratory Medicine, Perelman School of Medicine, University of Pennsylvania, Philadelphia, PA 19104, USA

HIV infection preferentially depletes HIV-specific CD4+ T cells, thereby impairing antiviral immunity. In this study, we explored the therapeutic utility of adoptively transferred CD4+ T cells expressing an HIV-specific chimeric antigen receptor (CAR_4) to restore CD4+ T cell function to the global HIV-specific immune response. We demonstrated that CAR_4 T cells directly suppressed *in vitro* HIV replication and eliminated virus-infected cells. Notably, CAR_4 T cells containing intracellular domains (ICDs) derived from the CD28 receptor family (ICOS and CD28) exhibited superior effector functions compared to the tumor necrosis factor receptor (TNFR) family ICDs (CD27, OX40, and 4-1BB). However, despite demonstrating limited *in vitro* efficacy, only HIV-resistant CAR_4 T cells expressing the 4-1BBζ ICD exhibited profound expansion, concomitant with reduced rebound viremia after antiretroviral therapy (ART) cessation and protection of CD4+ T cells (CAR^-) from HIV-induced depletion in humanized mice. Moreover, CAR_4 T cells enhanced the *in vivo* persistence and efficacy of HIV-specific CAR-modified CD8+ T cells expressing the CD28ζ ICD, which alone exhibited poor survival. Collectively, these studies demonstrate that HIV-resistant CAR_4 T cells can directly control HIV replication and augment the virus-specific CD8+ T cell response, highlighting the therapeutic potential of engineered CD4+ T cells to engender a functional HIV cure.

INTRODUCTION

HIV infection induces profound CD4+ T cell loss, resulting in impaired antiviral immunity and the onset of overt immunodeficiency.[1] In particular, HIV-specific CD4+ T cells are preferentially infected and exhibit defective immune responses characterized by poor proliferative capacity and interleukin (IL)-2 secretion.[2–5] The collapse of CD4+ T cell help during chronic infections compromises the generation of cytotoxic and memory CD8+ T cells,[6–8] leads to pronounced CD8+ T cell exhaustion,[6,9] and diminishes effective antibody production.[10,11] Moreover, CD4+ T cells exhibit direct cell-to-cell-mediated effector functions that contribute to disease resolution.[12–15] Indeed, subjects who spontaneously control HIV replication demonstrate a significant expansion of cytolytic CD4+ T cells that are associated with slower disease progression and improved clinical outcomes.[15,16] Given the functional heterogeneity of HIV-specific CD4+ T cells and their ability to coordinate global antiviral immunity, therapeutic interventions that restore or augment CD4+ T cell function will likely be critical for the development of effective HIV cure strategies.

Emerging data from cancer models demonstrate that CD4+ T cells redirected with a chimeric antigen receptor (CAR_4) can eradicate tumors in the absence of other immune cells,[17–19] indicating that CAR_4 T cells can act as primary effectors in addition to providing help to other leukocytes. CARs confer novel T cell specificity through expression of an extracellular antigen-binding domain fused to the intracellular CD3ζ chain and one or more costimulatory domains.[20,21] The choice of costimulatory domain alters the metabolic, phenotypic, and functional CAR T cell profile.[22,23] For instance, the CD28 costimulatory domain promotes glycolytic metabolism and the acquisition of an effector memory T cell phenotype capable of exhibiting rapid antitumor activity, whereas the 4-1BB domain supports the long-term *in vivo* persistence and development of central memory T cells reliant on oxidative phosphorylation for energy.[24–26] As such, the ability of CARs to engender unique T cell traits suggests that adoptively transferred HIV-specific CAR_4 T cells can restore many of the functions lost by HIV-induced destruction of CD4+ T cells.

However, since CD4+ T cells are the primary targets of HIV, efforts to make CAR_4 T cells resistant to infection must be employed to ensure durable responses.[27] Several approaches, including HIV coreceptor disruption,[28–30] as well as the overexpression of restriction factors[31,32] and fusion inhibitors,[33,34] have been developed to confer

Received 6 March 2020; accepted 12 May 2020;
https://doi.org/10.1016/j.ymthe.2020.05.012.

Correspondence: James L. Riley, Department of Microbiology, Center for Cellular Immunotherapies, Perelman School of Medicine, University of Pennsylvania, 3400 Civic Center Boulevard, Philadelphia, PA 19104, USA.
E-mail: rileyj@upenn.edu

This is an open access article under the CC BY license (http://creativecommons.org/licenses/by/4.0/).

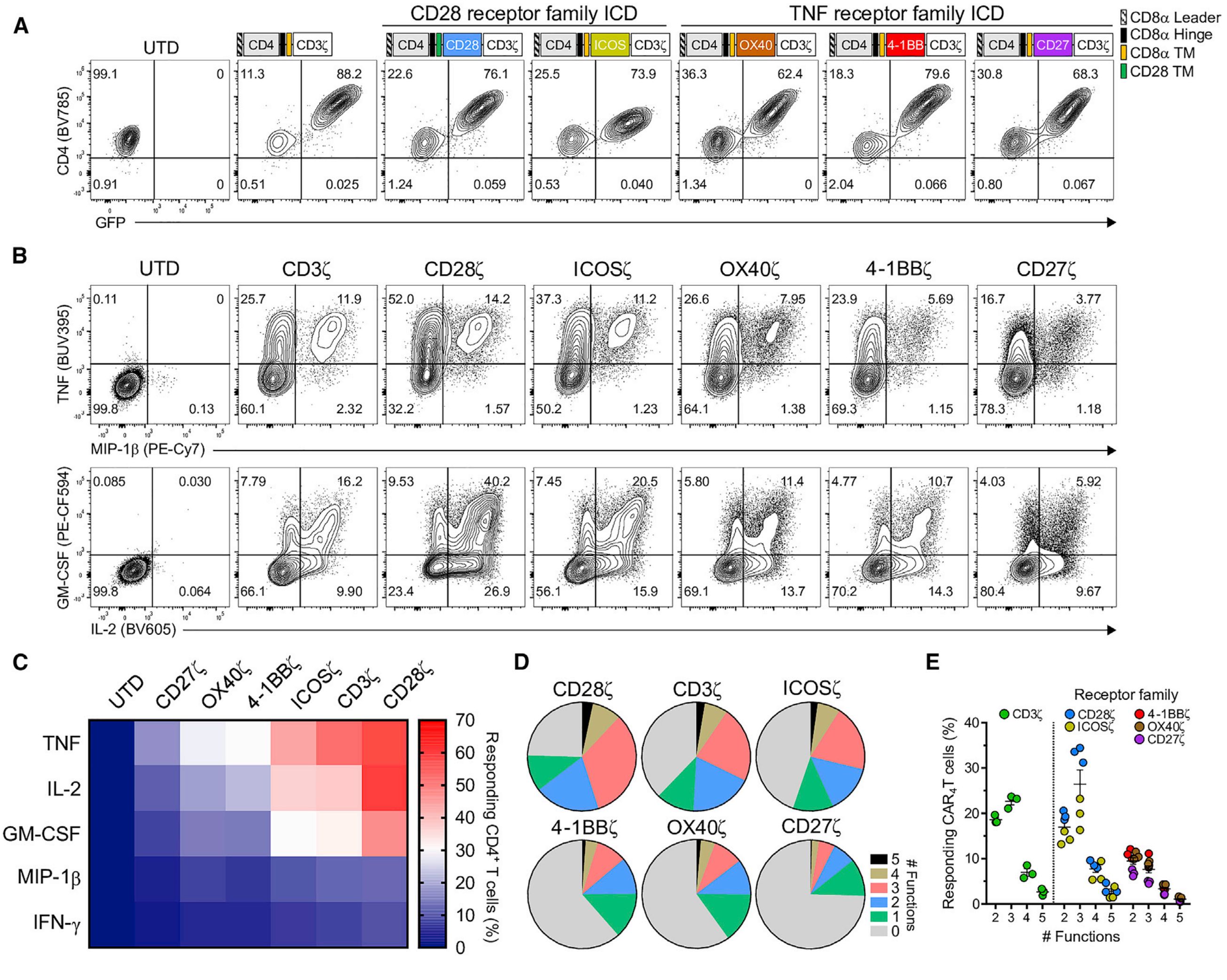

Figure 1. Intracellular Costimulatory Domains Differentially Modulate HIV-Specific CAR_4 T Cell Cytokine Expression

(A) Schematic representation of each CAR construct and fluorescence-activated cell sorting (FACS) plots identifying HIV-specific CAR-modified $CD4^+$ T cells (CAR_4) as GFP^+ and $CD4^+$ relative to untransduced $CD4^+$ T cells (UTD_4). Purified human $CD4^+$ T cells from a healthy human donor were activated with anti-CD3/CD28 Dynabeads and transduced with a lentiviral vector encoding one of six HIV-specific (CD4-based) CARs that express unique intracellular domains (ICDs), either CD3ζ (ζ), 4-1BBζ, CD28ζ, CD27ζ, OX40ζ, or ICOSζ. Each CAR was linked to GFP by an intervening T2A sequence to facilitate *in vitro* detection. (B) After 10 days of expansion, CAR_4 T cells were *in vitro* stimulated with HIV_{YU2} $GFP160^+$ K562 cells (K.Env), and intracellular cytokine analysis was performed. Data are representative of three donors. (C) Heatmap showing the percentage of responding CAR_4 T cells for each of the indicated cytokines. (D) Polyfunctionality profiles of combinatorial subsets for CAR_4 T cells producing 0–5 human cytokines: TNF, IL-2, IFN-γ, GM-CSF, and MIP-1β. (C and D) Data are the average of three donors. (E) Summary data of three donors per CAR_4 T cell population producing two or more cytokine functions after antigen stimulation. Lines indicate mean, and error bars show ±SEM.

HIV resistance. Of these, the fusion inhibitor C34 linked to CXCR4 is potent and interferes with the entry of diverse HIV strains regardless of their tropism.[35] Because of the need to protect and redirect $CD4^+$ T cells, progress to engineer the best $CD4^+$ T cells to control HIV replication has lagged behind studies examining $CD8^+$ T cells. Nonetheless, HIV-specific CAR_8 T cells have shown promise to inhibit virus replication, thereby providing the tools necessary to study CAR_4 T cells. For instance, we recently reported on an HIV-specific CAR that expresses CD4 as the HIV_{ENV} binding motif and contains the 4-1BB costimulatory domain.[36] This CAR was re-engineered from the original construct used during the first in-human clinical trials,[37–39] and it was selected for its improved antiviral potency in $CD8^+$ T cells[36] and potential to minimize virus escape.[40] Other approaches have incorporated a similar CD4-based CAR into $CD34^+$ hematopoietic stem cells to provide long-term *in vivo* production of HIV-specific immune cells,[41,42] or they have targeted infected cells using alterative antigen-binding moieties.[43–46] Collectively, these strategies highlight the promise of CAR T cell-based therapies against HIV; however, critical knowledge gaps remain, especially in our understanding of whether CAR_4 T cells need to

be engineered separately from CAR_8 T cells to achieve the optimal HIV-specific response.

In this study, we explored the therapeutic potential of CAR_4 T cell therapy to mitigate HIV pathogenesis. Data from cancer studies indicate that CAR_4 T cells require different costimulatory signals than CAR_8 T cells to engender superior immune responses.[47,48] However, using a panel of HIV-specific (CD4-based) CARs expressing distinct intracellular domains (ICDs), we show that only HIV-resistant, 4-1BB-costimulated CAR_4 T cells limit *in vivo* HIV infection, congruent with our previous work identifying 4-1BB as the optimal ICD for HIV-specific CAR_8 T cells.[36] Notably, ICDs derived from the CD28 receptor family (ICOS and CD28), which conferred the greatest CAR_4 T cell effector function *in vitro*, did not induce protective responses in humanized mice, suggesting that favorable disease outcomes are associated with factors beyond *in vitro* efficacy. These results, together with the observation that HIV-specific CAR_4 T cells augment the $CD8^+$ T cell response to infection, highlight the importance of exploiting engineered $CD4^+$ T cells in immunotherapies intended to treat chronic infections.

RESULTS

Costimulatory Domains Differentially Modulate HIV-Specific CAR_4 T Cell Cytokine Production

To engineer optimal HIV-specific $CD4^+$ T cells for use in HIV cure strategies, we first generated an array of CAR_4 T cells that expressed CD4, the natural ligand of HIV_{ENV}, as the extracellular antigen-binding moiety fused to an ICD comprising the TCR CD3ζ (ζ) chain and one costimulatory domain derived from CD27, OX40, 4-1BB, ICOS, or CD28. The CAR containing the CD28ζ ICD was linked to the CD28 transmembrane (TM) domain, while the remaining CARs contained the CD8α TM domain. All of the HIV-specific CARs were efficiently expressed on the cell surface and could be identified by both the overexpression of CD4 relative to untransduced $CD4^+$ T cells (UTD_4) and co-expression of GFP, which was linked to each CAR by an intervening T2A sequence (Figure 1A).

We then assessed the effects of individual ICDs on the *in vitro* effector function of CAR_4 T cells after antigen-specific stimulation with target cells expressing HIV_{ENV}. All of the CAR_4 T cell types upregulated tumor necrosis factor (TNF), macrophage-inflammatory protein (MIP)-1β, granulocyte-macrophage colony-stimulating factor (GM-CSF), IL-2 and interferon (IFN)-γ following antigen exposure; however, CAR_4 T cells containing the CD28 receptor family ICDs exhibited the greatest production of these cytokines (Figures 1B and 1C; Figure S1). In particular, more than 75% of CD28-costimulated CAR_4 T cells elicited a cytokine response to stimulation, exemplified by IL-2 and TNF expression. Interestingly, we observed low expression levels of other cytokines associated with T helper phenotypes, including IL-17A, IL-21, IL-22, IL-4, and IL-13 (Figure S2). Moreover, combinatorial cytokine analysis indicated that ICDs from the CD28 receptor family increased the frequency of polyfunctional responses (Figure 1D), where exhibiting three or more cytokine functions contributed more than 20% of the total response and was mainly driven by the $GM\text{-}CSF^+IL\text{-}2^+TNF^+$ subset, compared to less than 10% by the TNF receptor (TNFR) family ICDs (CD27, OX40, and 4-1BB) (Figures 1D and 1E). Somewhat paradoxically, in the absence of costimulation, the CD3ζ ICD induced robust cytokine production (Figures 1C–1E), which may result from the high expression of this CAR on the $CD4^+$ T cell surface (Figure S3). Taken together, these data demonstrate that HIV-specific CARs redirect $CD4^+$ T cell specificity, and distinct costimulatory signals within the CAR differentially modulate the magnitude and breadth of cytokine expression.

HIV-Specific CAR_4 T Cells Durably Suppress *In Vitro* HIV Replication

To determine whether HIV-specific CAR_4 T cells could limit the spread of HIV infection, we performed an *in vitro* suppression assay comparing the ability CAR_4 T cells containing different ICDs to inhibit virus outgrowth. In this study, activated $CD4^+$ T cells were infected with HIV_{BAL} for 24 h before co-culture with CAR_4 or UTD_4 T cells at different effector-to-target (E:T) ratios, and virus replication was measured during 8 days by determining the frequency of intracellular HIV_{GAG}^+ (CAR^-) cells. We observed widespread virus replication when infected cells were cultured with UTD_4 T cells, whereas each CAR_4 T cell population durably suppressed HIV at a 1:25 and 1:50 E:T ratio (Figures S4A and S4B; Figures 2A and 2B). However, further dilution of CAR_4 T cells expressing the TNFR family ICDs resulted in rapid loss of virus control (Figures 2A and 2C; Figure S4C). In contrast, CAR_4 T cells containing the CD28 receptor family and CD3ζ ICDs potently suppressed virus spread at lower E:T ratios (Figures 2C and 2D; Figure S4D).

At the same time, we compared the protective role of CAR_4 T cells to that of HIV-specific CAR_8 T cells expressing the 4-1BBζ ICD. We previously demonstrated that 4-1BB costimulation conferred optimal CAR_8 T cell antiviral activity *in vivo*,[36] and now this construct is currently used in a phase I clinical trial (ClinicalTrials.gov: NCT03617198). CAR_8 T cells exhibited potent HIV suppression below a 1:100 E:T ratio (Figure 2E; Figure S5A), and, notably, CAR_4 T cells expressing the CD28ζ and CD3ζ ICDs controlled virus spread to the same extent (Figure 2F). For a direct comparison, we noted that CAR_4 T cells expressing the 4-1BBζ ICD were approximately 3-fold less suppressive than CAR_8 T cells harboring the same ICD (Figure S5B). These results indicated that HIV-specific CAR_4 T cells can solely control *in vitro* HIV replication, and that the ICD integrated into the CAR modulates the ability of CAR_4 T cells to directly inhibit virus outgrowth.

HIV-Specific CAR_4 T Cells Directly Eliminate HIV-Infected Cells *In Vitro*

The effector mechanisms employed by $CD4^+$ T cells largely fall into two categories: first, the production of broad-acting soluble factors, such as IFN-γ and TNF-α, that promote an antiviral state in surrounding tissue,[49] and second, direct cytotoxic activity.[50–52] In the context of CD19-targeted CAR T cell therapy, CAR_4 T cells directly engage and kill tumor cells *in vitro* to the same extent as CAR_8 T cells, but they exhibit slower kinetics of cytotoxicity.[53] Similarly,

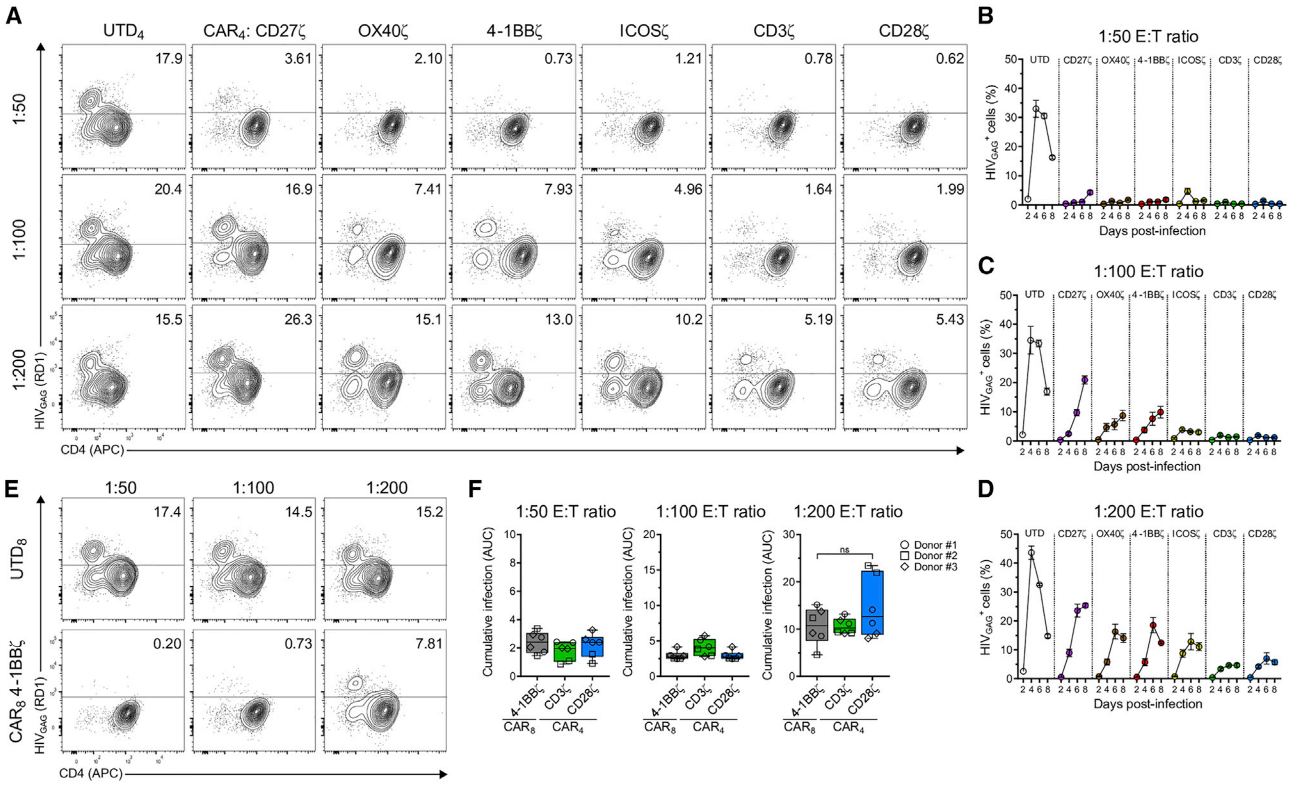

Figure 2. HIV-Specific CAR_4 T Cells Durably Suppress *In Vitro* HIV Replication

HIV-specific CAR T cells and UTD T cells were mixed separately with HIV_{BAL}-challenged $CD4^+$ T cells at the indicated effector-to-target (E:T) ratios, and the level of virus spread was monitored by intracellular staining and flow cytometry for HIV_{GAG} antigen on days 2, 4, 6, and 8 after co-culture. (A) FACS plots indicate the frequency of HIV_{GAG}^+ cells (CAR^-) 8 days after co-culture with each CAR_4 T cell type or UTD_4 T cells. (B–D) Summary of the frequency of HIV_{GAG}^+ cells at days 2, 4, 6, and 8 after co-culture at (B) 1:50, (C) 1:100, and (D) 1:200 E:T ratios. For (B)–(D), symbols represent the average of three distinct donors in duplicate, and error bars show ±SEM. (E) FACS plots show the frequency of HIV_{GAG}^+ cells (CAR^-CD8^-) 8 days after co-culture with UTD_8 or HIV-specific CAR_8 T cells expressing the 4-1BBζ intracellular domain. (F) Cumulative infection calculated by area under the curve from the frequency of HIV_{GAG}^+ cells at days 2, 4, 6, and 8 after co-culture. Data are represented as box-and-whisker plots and bars show minimum and maximum values. Symbols indicate unique donors performed in duplicate. A Kruskal-Wallis test and Dunn's multiple comparison test were used to determine significance. ns, not significant ($p > 0.05$).

we examined whether HIV-specific CAR_4 T cells elicit cytotoxic function by performing a short-term killing assay using primary HIV-infected cells as targets.[54] CAR_4 T cells expressing the CD3ζ ICD eliminated target cells in a dose-dependent manner, achieving an 80% reduction in the frequency of HIV_{GAG}^+ cells (from 30% to 6% HIV_{GAG}^+ cells) after 24 h (4:1 E:T ratio) (Figures 3A and 3B). The killing exhibited by CAR_4 T cells was HIV-specific, as indicated by the lack of infected cell lysis when cultured with UTD_4 T cells (Figure 3B). Furthermore, when we expanded the killing assay to include CAR_4 T cells encoding distinct costimulatory domains, we observed marked reductions in the number of HIV_{GAG}^+ cells mediated by CAR_4 T cells expressing the CD28 receptor family ICDs or CD3ζ. Notably, the level of infected cell killing was equivalent between these CAR_4 T cell types and CAR_8 T cells (Figure 3C; Figure S6). In addition, inclusion of the TNFR family ICDs conferred cytolytic activity to CAR_4 T cells, but not to the same magnitude (Figure 3C; Figure S6).

We next examined whether CAR_4 T cells could trigger activation of caspase-3 in HIV-infected cells, which acts as the primary executioner in the cell-death apoptosis pathway[55] and is a direct substrate of granzyme (Gzm) B.[56] Indeed, CAR_4 T cells induced cleavage of caspase-3 in HIV_{GAG}^+ cells (Figures 3D and 3E), suggesting that virus-infected cell death is mediated in part by caspase-dependent mechanisms. Furthermore, we assessed the expression of cytolytic effector molecules, including GzmB, GzmA, and GzmM, in all CAR_4 T cell populations. CAR_4 T cells containing the CD28 receptor family ICDs or CD3ζ harbored the highest level of GzmB and GzmA (Figure 3F; Figure S7A), while GzmM expression was negligible (Figure S7B), suggesting that signaling from ICDs influences the cytotoxic potential of CAR_4 T cells independent of antigen exposure. However, these molecules were differentially regulated following *in vitro* antigen-specific stimulation, where after we observed a substantial increase in GzmB expression by all CAR_4 T cell types, exemplified by the CD28 receptor family ICDs or CD3ζ (Figure 3F; Figure S7C), while

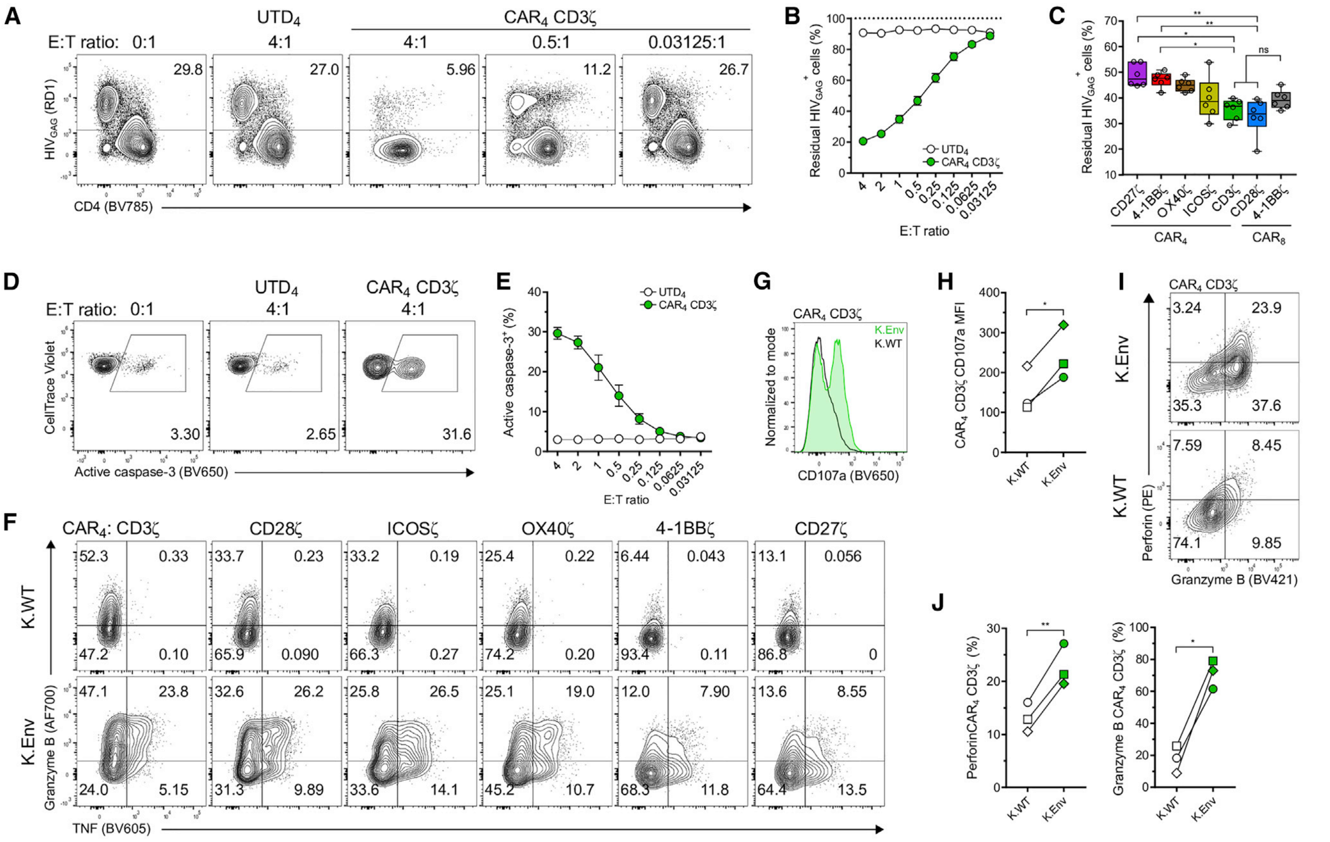

Figure 3. HIV-Specific CAR_4 T Cells Exhibit Cytolytic Function against Virus-Infected $CD4^+$ T Cells

(A–E) CellTrace Violet-labeled, HIV_{BAL}-infected $CD4^+$ T cells (30% HIV_{GAG}^+) were cultured with HIV-specific CAR or UTD T cells at the indicated E:T ratios. 24 h later the frequency of HIV_{GAG}^+ cells (live CTV^+CAR^-) was assessed by intracellular staining and flow cytometry for HIV_{GAG} antigen. (A and B) FACS plots show the frequency of HIV_{GAG}^+ cells (A), and summary data indicate the frequency of residual HIV_{GAG}^+ cells after co-culture with UTD_4 or CAR_4 T cells expressing the CD3ζ ICD (B). (C) Summary data of elimination assay at the 0.125:1 E:T ratio. (D and E) FACS plots (D) and summary data (E) show frequency of active caspase-3 within HIV_{GAG}^+ cells after co-culture with UTD_4 or CAR_4 T cells expressing the CD3ζ ICD. (F) After 10 days of expansion, CAR_4 T cells were stimulated with K.Env or wild-type K562 cells (K.WT), and FACS plots show upregulation of granzyme B and TNF. Data are representative of three donors. (G and H) Histogram (G) and mean fluorescence intensity (MFI) (H) indicate CD107a mobilization in CAR_4 T cells after *in vitro* stimulation. (I and J) FACS plots (I) and summary data (J) show the coordinated upregulation of perforin and granzyme B in CAR_4 T cells expressing the CD3ζ ICD after *in vitro* stimulation. (B and E) Symbols indicate average of three donors performed in triplicate. Error bars show ±SEM. (C) Data are represented as box-and-whisker plots, and bars show minimum and maximum values. Symbols indicate three donors performed in duplicate. A Kruskal-Wallis test and Dunn's multiple comparison test were used to determine significance. (H and J) Data show three donors, and significance was calculated using a paired Student's t test. $^*p < 0.05$, $^{**}p < 0.01$ (for all data).

GzmA expression waned (Figures S7A and S7D). Moreover, stimulation of CAR_4 T cells resulted in the detection of CD107a, which occurs as cytotoxic granules mobilize from the cytosol to the cell surface (Figures 3G and 3H),[57] and was coupled with the coordinated upregulation of perforin and GzmB (Figures 3I and 3J). Taken together, these data demonstrate that CAR_4 T cells exhibit *in vitro* cytotoxic function that can at least be partially attributed to granule-mediated cytolysis.

4-1BB-Costimulated CAR_4 T Cells Mitigate HIV Pathogenesis *In Vivo*

We next sought to determine the *in vivo* therapeutic potential of distinct HIV-specific CAR_4 T cell populations using a humanized mouse model of HIV infection.[36] To do so, we humanized NSG mice by adoptively transferring normal donor, CD8-depleted peripheral blood mononuclear cells (PBMCs). Two weeks later, the mice were infused with autologous, HIV_{BAL}-infected $CD4^+$ T cells that were treated with antiretroviral therapy (ART) *in vitro* prior to infusion and the mice received daily ART (Figure 4A). After 3 days, mice were allocated into groups based on the level of $CD4^+$ T cells in peripheral blood (Figure 4B) and infused with either one of the six CAR_4 T cell types or control CAR_4 T cells expressing a truncated CD3ζ ICD (CD3Δζ) (Figure 4A). We rendered each CAR_4 T cell product HIV-resistant by co-transduction with the HIV fusion inhibitor C34-CXCR4,[35] which was linked to NGFR (nerve growth factor receptor) by an intervening T2A sequence (Figure S8A). Prior to

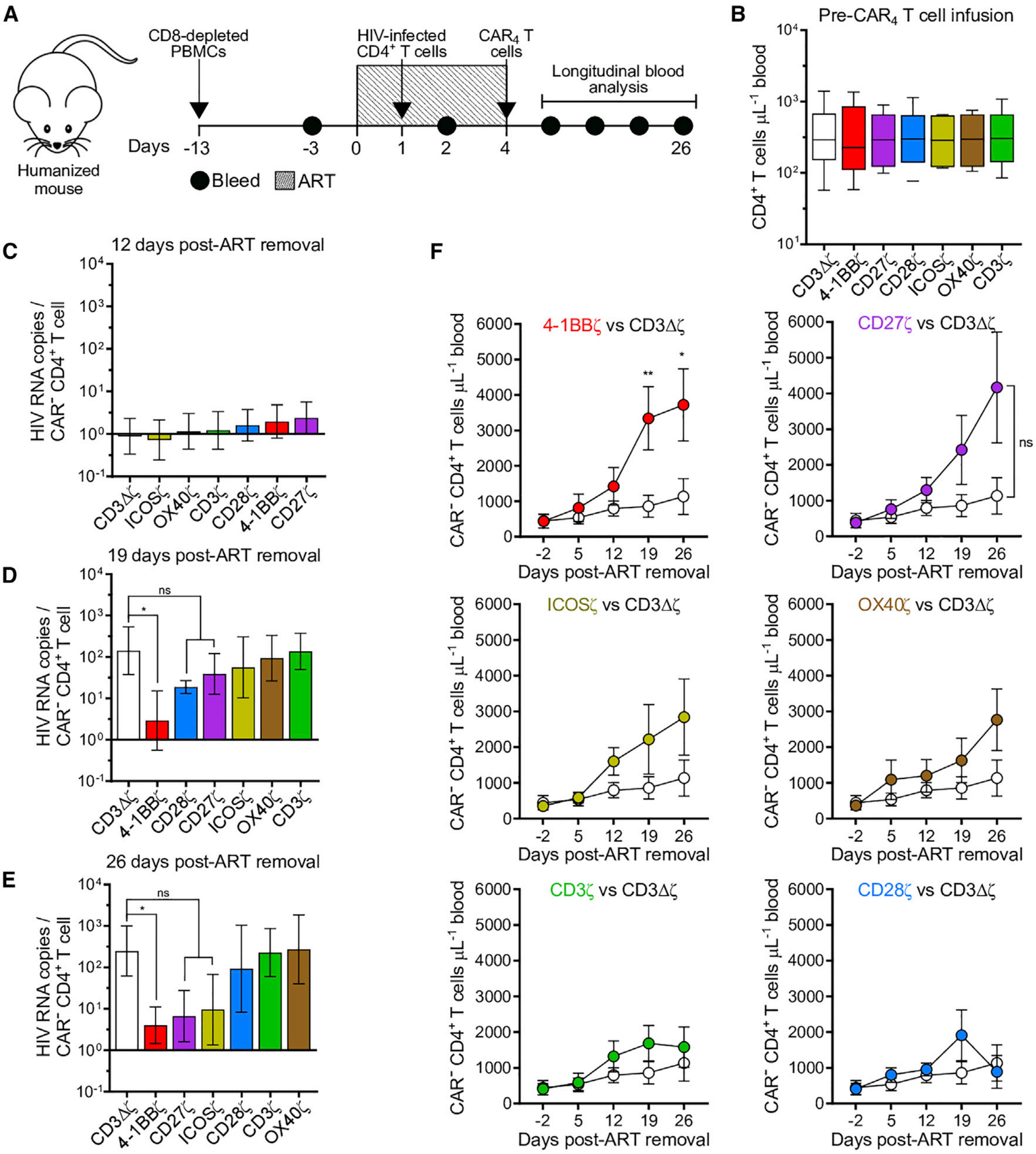

Figure 4. HIV-Specific 4-1BB-Costimulated CAR_4 T Cells Mitigate HIV Disease Progression after ART Removal

(A) Experimental design. NSG mice were infused with CD8-depleted PBMCs from a healthy human donor. Two weeks later, mice initiated daily ART for 1 week and were infused with autologous, *in vitro* HIV_{BAL}-infected $CD4^+$ T cells. Mice were allocated into seven groups (n = 6–7) based on $CD4^+$ T cell engraftment, and then each mouse received 2.5×10^6 HIV-resistant (C34-$CXCR4^+$) CAR_4 T cells expressing one of the six intracellular domains (ICDs), or inactive control CAR_4 T cells expressing a truncated CD3ζ ICD followed by ART cessation. (B) Concentration of peripheral blood $CD4^+$ T cells prior to CAR T cell infusion. Data are represented as box-and-whisker plots, and bars show minimum and maximum values. (C–E) HIV RNA copies/mL plasma normalized to contemporaneous peripheral blood $CD4^+$ T cell concentration (CAR^-) (C) 12 days, (D) 19 days, and (E) 26 days after ART removal. (F) Longitudinal concentration of $CD4^+$ T cells (CAR^-) in peripheral blood. (C–F) Bars and symbols indicate mean, and error bars show ±SEM. Significance was calculated using a Wilcoxon rank sum test. *$p < 0.05$, **$p < 0.01$. ns, not significant ($p > 0.05$).

CAR_4 T cell infusion, the cells were positively selected for NGFR expression, achieving ≥90% $NGFR^+$ (C34-$CXCR4^+$) T cell products (Figure S8B). Of note, expression of C34-CXCR4 did not augment the functional potency of CAR T cells, as unprotected CAR T cells suppressed *in vitro* HIV replication to the same extent as CAR_4 T cells expressing C34-CXCR4 (Figure S8C).

We interrupted ART immediately after CAR_4 T cell infusion and measured the kinetics of HIV rebound. In this model, the magnitude of virus replication correlates with the number of $CD4^+$ T cells,[58,59] and thus to ensure fair comparison among treatment groups, we normalized plasma HIV RNA at each time point to the contemporaneous level of peripheral blood $CD4^+$ T cells (CAR^-). We observed that only CAR_4 T cells expressing the 4-1BBζ ICD reduced the magnitude of rebound viremia for 26 days after ART removal compared to control CAR_4 T cell-treated mice (Figures 4C–4E; Table S1). This difference remained significant without normalizing viral load, particularly when assessing cumulative viral burden in plasma (Figure S9). Furthermore, mice treated with 4-1BB-costimulated CAR_4 T cells exhibited durable protection against HIV-induced $CD4^+$ T cell (CAR^-) depletion (Figure 4F). These observations were striking given that the 4-1BBζ ICD underperformed in every *in vitro* measure of CAR_4 T cell function, especially compared to the CD28 receptor family ICDs.

4-1BB-Costimulated CAR_4 T Cells Exhibit Profound *In Vivo* Expansion

Next, we characterized the immunologic response inherent to each CAR_4 T cell type to viral recrudescence after ART cessation. CAR_4 T cells were readily detected in peripheral blood by the co-expression of GFP and NGFR (Figure 5A). 4-1BB-costimulated CAR_4 T cells exhibited rapid and profound expansion, reaching a median peak concentration of 511 cells/μL blood (34–2,732 cells/μL) (Figure 5B), and they maintained greater long-term survival compared to the other CAR_4 T cell populations (Figure 5C). Surprisingly, CAR_4 T cells expressing CD28 receptor family ICDs, which demonstrated superior *in vitro* potency, exhibited poor *in vivo* expansion and persistence (Figures 5B and 5C). Moreover, CAR_4 T cells containing the TNFR family ICDs expressed lower levels of inhibitory receptors, including TIGIT and PD-1, early after ART cessation, which may have, in addition to the ICD, modulated early *in vivo* proliferation kinetics (Figures 5D and 5E; Figure S10). Notably, the magnitude of CAR_4 T cell expansion across all types, but exemplified by the 4-1BBζ ICD, correlated with reductions in plasma viremia (Figure 5F) and $CD4^+$ T cell (CAR^-) preservation (Figure 5G). Taken together, these data demonstrate that CAR_4 T cells expressing the 4-1BB costimulatory domain possess superior antiviral activity, and they highlight that factors such as expansion potential are critical to mitigate HIV pathogenesis.

HIV-Specific CAR_4 T Cells Improve the Proliferation and Survival of Co-injected CAR_8 T Cells *In Vivo*

We hypothesized that HIV-specific CAR_4 T cells will exhibit T cell help to other lymphocytes *in vivo*. To test this, we examined whether CAR_4 T cells expressing the 4-1BBζ ICD could augment the antiviral function of HIV-specific CAR_8 T cells after ART removal in HIV-infected humanized mice. We recapitulated the study as described in Figure 4A, but here, mice were allocated into groups that received an HIV-resistant (C34-$CXCR4^+$) CAR T cell product consisting of either CAR_8 T cells alone (2.5×10^6 CAR^+ cells), a 1:1 mixture of CAR_4 and CAR_8 T cells (1.25×10^6 CAR^+ cells/cell type), or a 1:1 mixture of inactive control CAR_4 and CAR_8 T cells (1.25×10^6 CAR^+ cells/cell type) expressing the CD3Δζ ICD. Of note, CAR_8 T cells expressed the CD28ζ ICD, which previously demonstrated marginal *in vivo* expansion and protection against HIV infection.[36] We observed that co-injection of CAR_4 T cells enhanced the expansion (Figures 6A and 6B) and persistence (Figure 6C) of CD28-costimulated CAR_8 T cells despite infusing half the dose, whereas alone these cells exhibited limited proliferation relative to control CAR_8 T cells (Figure S11). In a separate study, CAR_4 T cells also accelerated the early expansion kinetics of CAR_8 T cells expressing the 4-1BBζ ICD (Figure S12); this effect was surprising given the remarkable proliferation these cells exhibit on their own after ART cessation.[36]

We next determined whether the co-infusion of CAR_4 T cells improved virologic outcomes following ART withdrawal. Notably, in this study we observed faster HIV rebound kinetics as viremia was detectable in mice 5 days after ART interruption (Figure 6D) compared to 12 days in our prior study (Figure 4C). Nevertheless, combination therapy with CAR_4 T cells and CD28-costimulated CAR_8 T cells reduced rebound viremia (Figure 6E) and effectively limited HIV-induced depletion of $CD4^+$ T cells (CAR^-) (Figure 6F). In contrast, treatment with CAR_8 T cells alone exhibited a transient reduction in viral load (Figure 6E) but was unable to mitigate $CD4^+$ T cell loss relative to control CAR T cell-treated mice (Figure 6F). Taken together, these findings highlight that CAR_4 T cells provide T cell help by improving the *in vivo* expansion and survival of CAR_8 T cells, and that combination therapy improves control over HIV pathogenesis.

DISCUSSION

HIV preferentially infects HIV-specific $CD4^+$ T cells, leading to the collapse of $CD4^+$ T cell help and impaired antiviral immunity.[1,2] Thus, therapeutic approaches that restore $CD4^+$ T cell function, such as adoptive T cell therapy, will likely be a critical component of any HIV functional cure or eradication strategy.[60] In this study, we interrogated the therapeutic potential of HIV-specific CAR_4 T cells to limit HIV infection *in vitro* and after ART withdrawal in humanized mice. Given how costimulation tunes the functional heterogeneity of $CD4^+$ T cells,[61,62] we initially characterized how distinct ICDs modulate *in vitro* CAR_4 T cell functions. We demonstrated that the CD28 receptor family ICDs (ICOS and CD28) induced potent functional profiles compared to the TNFR family ICDs (CD27, OX40, and 4-1BB), exemplified by polyfunctional cytokine responses and direct suppression of *in vitro* virus replication, which rivaled CAR_8 T cell-mediated control of HIV. However, we did not observe an association between ICDs that conferred optimal *in vitro* function and the ability to mitigate HIV pathogenesis in humanized mice, suggesting that factors driving *in vivo* CAR_4 T cell-mediated efficacy are not solely predicted *in vitro*.

We identified CAR_4 T cell expansion as a correlate of *in vivo* antiviral efficacy, supporting observations that CAR T cell persistence is necessary to engender long-term remission of certain B cell

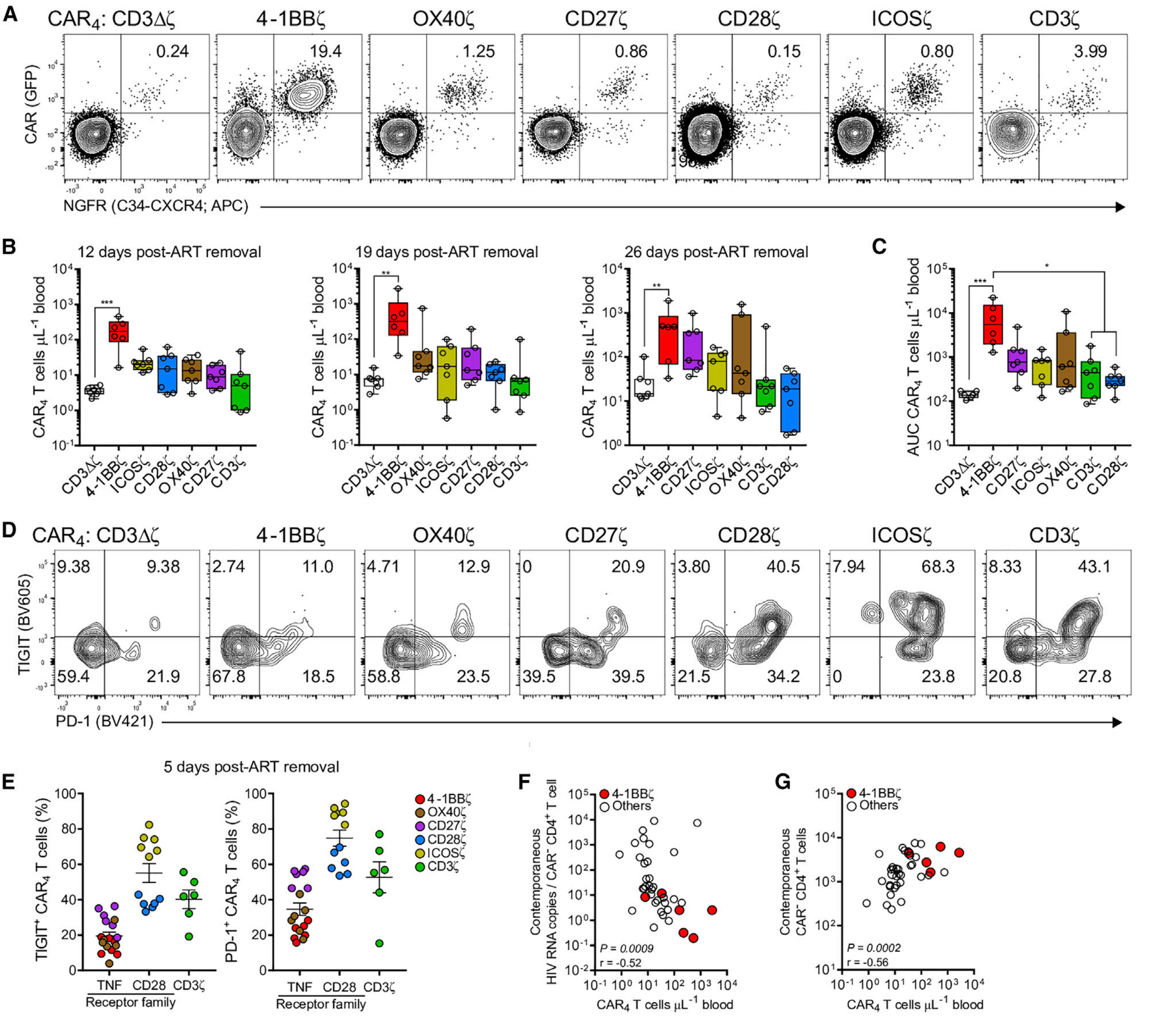

Figure 5. 4-1BB Costimulation Potentiates *In Vivo* HIV-Specific CAR_4 T Cell Expansion and Persistence

(A) FACS plots show the detection of each CAR_4 T cell type in peripheral blood 26 days after ART removal in HIV-infected humanized mice. CAR_4 T cells are identified by the co-expression of GFP and NGFR, which are linked by intervening T2A sequences to the indicated CD4-based CAR and C34-CXCR4, respectively. (B) Peripheral blood concentration of each CAR_4 T cell type at days 12, 19, and 26 after ART removal. (C) Cumulative peripheral CAR_4 T cell persistence measured by area under the curve from days 5, 12, 19, and 26 after ART removal. (D and E) FACS plots (D) and summary data (E) show the frequency of TIGIT and PD-1 expression on each CAR_4 T cell type 5 days after ART removal. (F and G) Correlation between CAR_4 T cell concentration 19 days after ART removal and contemporaneous viral load (F), and $CD4^+$ T cell (CAR^-) concentration (G). Red symbols indicate 4-1BB-costimulated CAR_4 T cells, and white symbols indicate the remaining CAR_4 T cell types. (B and C) Data are represented as box-and-whisker plots, and bars show minimum and maximum values. Each symbol denotes one mouse. A Wilcoxon rank sum test and (F and G) Spearman correlation were used to test for significance. $^*p < 0.05$, $^{**}p < 0.01$, $^{***}p < 0.001$ (for all data).

malignancies.[63–65] Despite demonstrating limited *in vitro* efficacy, only HIV-resistant (C34-$CXCR4^+$) CAR_4 T cells expressing the 4-1BBζ ICD exhibited profound expansion and survival, and inhibited disease progression after ART removal. The remaining ICDs induced marginal proliferation notwithstanding abundant HIV antigen, in contrast to other studies demonstrating that ICOS,[17,47] CD27,[66] and CD28[67,68] costimulatory signals mediate *in vivo* CAR_4 T cell expansion and tumor eradication. Taken together, these data show that the 4-1BBζ ICD is necessary to potentiate rapid *in vivo* proliferation and long-term survival of CAR_4 T cells during HIV infection. This finding is critical given that the stability of the latent reservoir in humans[69,70] likely necessitates the persistence of CAR T cells for months, years, or decades after infusion to respond to HIV reactivation.[71]

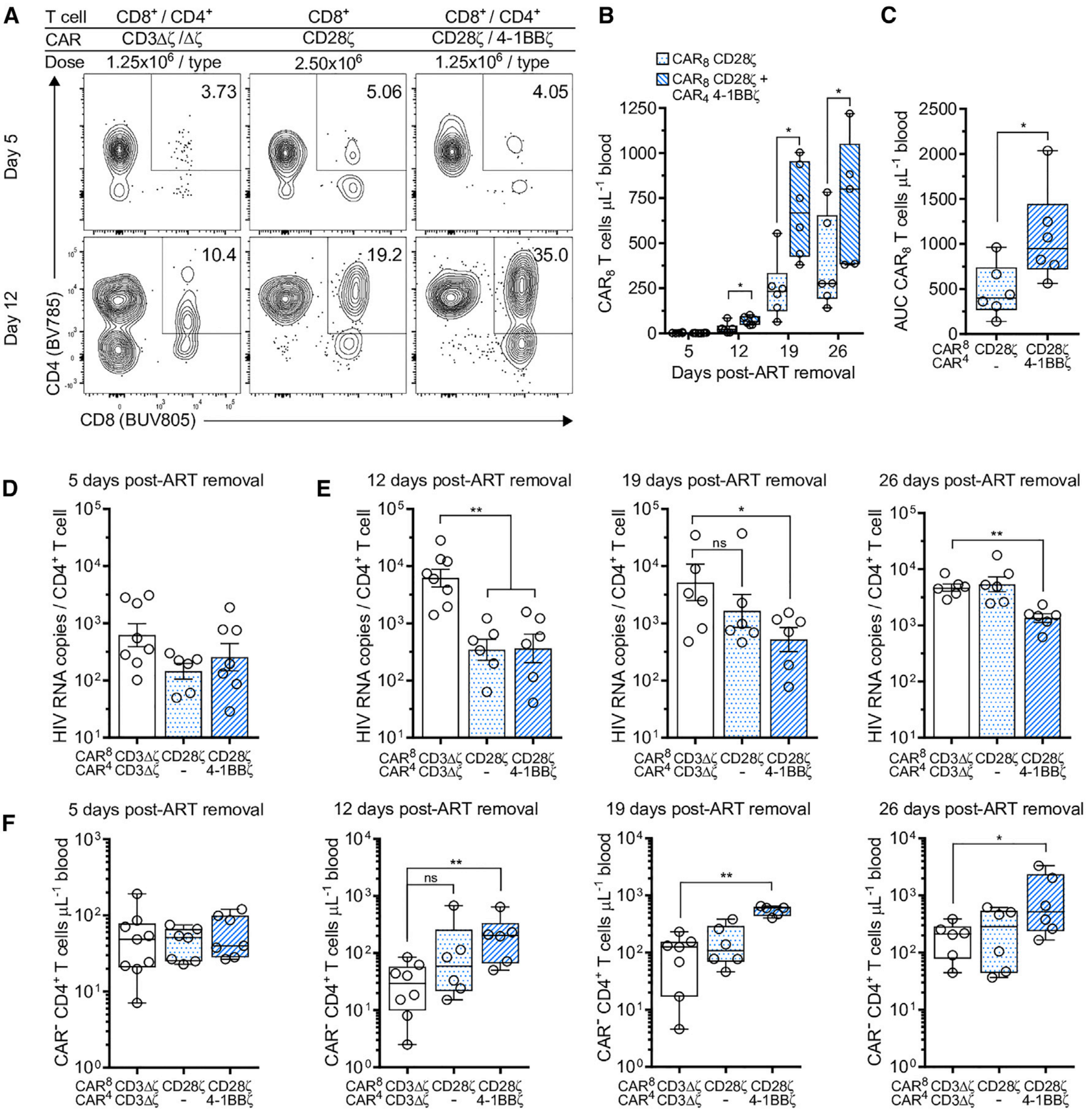

Figure 6. Co-injection of HIV-Specific CAR_4 T Cells Improves CAR_8 T Cell Expansion and Post-ART Control of HIV Infection

NSG mice were infused with CD8-depleted PBMCs from a healthy human donor. Two weeks later, mice were given ART daily for 1 week and were infused with autologous, *in vitro* HIV_{BAL}-infected $CD4^+$ T cells. Mice were allocated into three groups (n = 7–8) based on $CD4^+$ T cell engraftment, and then each group received either HIV-resistant (C34-$CXCR4^+$) CAR_8 (CD28ζ) T cells (2.5 × 10^6 CAR^+ cells/mouse), a 1:1 ratio of CAR_4 (4-1BBζ) and CAR_8 (CD28ζ) T cells (1.25 × 10^6 CAR^+ cells/cell type/mouse), or a 1:1 ratio of inactive control CAR_4 and CAR_8 T cells (1.25 × 10^6 CAR^+ cells/cell type/mouse) expressing the CD3Δζ ICD followed by ART interruption. (A) FACS plots show the frequency of peripheral CAR_8 T cells identified by the overexpression of CD4 on the $CD8^+$ T cell surface. (B) Longitudinal concentration of peripheral CAR_8 T cells after ART interruption. (C) Cumulative peripheral CAR_8 T cells persistence measured by area under the curve from days 5, 12, 19, and 26 after ART removal. (D and E) HIV RNA copies/mL plasma normalized to contemporaneous peripheral $CD4^+$ T cell (CAR^-) concentration at (D) 5 days and (E) 12, 19, and 26 days after ART removal. (F) Longitudinal concentration of $CD4^+$ T cells (CAR^-) in peripheral blood. (B, C, and F) Data are represented as box-and-whisker plots, and bars show minimum and maximum values. (D and E) Bars indicate mean, and errors show ±SEM. For all data, each symbol denotes one mouse, and a Wilcoxon rank sum test was used to calculate significance. *$p < 0.05$, **$p < 0.01$. ns, not significant ($p > 0.05$).

Although the humanized mouse model described herein is well suited to evaluate therapeutic interventions that mitigate active HIV replication and $CD4^+$ T cell depletion, these animals eventually develop graft- versus-host disease (GVHD).[72] This xenoreactivity induces substantial cellular activation and proliferation, resulting in the clinical manifestation of disease within 3–4 weeks after infusion,[73] which diminishes the time to study the durability of CAR T cell therapy, and limits our ability to recapitulate all aspects of treatment performed in

HIV-infected individuals. For instance, the constant immune activation drives supraphysiologic levels of virus replication and precludes the establishment of a latent HIV reservoir, which in humans is comprised of a heterogeneous population of HIV-infected, transcriptionally quiescent memory $CD4^+$ T cells.[69,74] In contrast, treatments specifically targeting the latent reservoir may instead be accurately reflected in humanized BLT mice, a more complex small-animal model of HIV infection, which supports the generation of a latent reservoir under ART.[75,76] Nevertheless, our *in vivo* model enabled us to directly compare the ability of multiple CAR T cell products side by side to mitigate hallmarks of HIV disease progression following ART cessation, which is congruent with the study objectives being investigated by in-human clinical trials (ClinicalTrials.gov: NCT03617198).

Moreover, these findings along with our previous studies indicate that 4-1BB costimulation is essential for both CAR_4 and CAR_8 T cells to mitigate *in vivo* HIV pathogenesis after ART cessation,[36] which contrasts with recent data demonstrating that mesothelin-specific CAR_4 and CAR_8 T cells rely on distinct costimulatory signals for optimal function.[47] However, within this study, we did observe the CD28ζ ICD differentially impacted the efficacy of CAR_4 and CAR_8 T cell therapies *in vivo*. For instance, CD28-costimulated CAR_4 T cells failed to proliferate and mount effective immunity against viral recrudescence compared to mice treated with 4-1BB-costimulated CAR_4 T cells (Figures 4D–4F). In contrast, the data described herein support our previous work,[36] showing that CAR_8 T cells expressing the CD28ζ ICD were capable of reducing viremia and delaying $CD4^+$ T cell (CAR^-) depletion to the same extent as 4-1BB-costimulated CAR_8 T cells early after ART cessation. These findings reinforce the notion that costimulatory signals that engender favorable CAR T cell responses are likely both disease- and cell type-specific.[22]

In addition to CAR_4 T cells exhibiting direct control of *in vitro* HIV replication, we reasoned that they could also restore $CD4^+$ T cell help that is lost as a consequence of viral infection. To this end, we demonstrated that co-injection of CAR_4 T cells enhanced the *in vivo* proliferation kinetics and survival of CD28-costimulated CAR_8 T cells, concomitant with reduced HIV replication. Moreover, we have shown that *in vitro* activation of 4-1BB-costimulated CAR_4 T cells upregulates IL-2 and IL-21, which are cytokines that augment the function of $CD8^+$ T cells during viral infection,[77–79] and thus may have contributed to the sustained CAR_8 T cell response that we observed *in vivo*. Furthermore, it is reasonable to think that CAR_4 T cell help will benefit endogenous, HIV-specific immunity. For example, previous studies have demonstrated that HIV-specific cytolytic $CD4^+$ and $CD8^+$ T cells exhibit strong cooperativity to suppress infection *in vitro*,[80] and the addition of vaccine-primed HIV-specific $CD4^+$ T cells reinvigorate the proliferative capacity of $CD8^+$ T cells isolated from chronic infection.[81] Finally, since our CAR_4 T cells still express a functional TCR, it is likely that a portion of the infused CAR_4 T cells maintain specificity to other pathogens and are able to confer help to immune responses toward other infections, including Epstein-Barr virus (EBV), flu, and cytomegalovirus (CMV). Interestingly, it was reported that repeated TCR stimulation of CAR T cells results in exhaustion of CAR_8, but not CAR_4, T cells,[82] suggesting that CAR_4 T cell-mediated help could be durable.

The ability of HIV-specific CAR_4 T cells to control HIV replication, as well as enable the function of other immune cells, highlights the therapeutic potential of this T cell population. However, we expect that next-generation CAR_4 T cells will need to be infused into HIV-infected individuals as part of a defined formulation with CAR_8 T cells to achieve a synergistic antiviral effect, similar to the cooperativity between these T cells during cancer treatment.[65,83] Moreover, the potential of CAR T cell therapy alone to engender positive clinical outcomes may only occur after ART cessation when sufficient viral antigen is present to induce CAR T cell activation, as opposed to eliminating the latent reservoir during ART when antigen is restricted. In this way, CAR T cell therapy may resolve post-peak rebound viremia and contribute to an ART-free remission of HIV. Collectively, the findings described herein provide insight regarding the potential of engineered $CD4^+$ T cells to both augment and restore the function of HIV-specific $CD4^+$ T cells that is typically lost by virus-associated depletion, which could serve to reinvigorate broad and enduring immune responses that enable immune control over HIV.

MATERIALS AND METHODS

Ethics Statement

Humanized mouse experiments performed at the University of Pennsylvania were approved by the University of Pennsylvania Institutional Animal Care and Use Committee (IACUC) under approval protocol 805606. All animal studies were carried out in accordance with recommendations in the *Guide for the Care and Use of Laboratory Animals* of the National Institutes of Health. Peripheral blood mononuclear cells and purified adult human $CD8^+$ and $CD4^+$ T cells were obtained by the University of Pennsylvania Human Immunology Core/Center for AIDS Research (CFAR) Immunology Core from de-identified healthy donors.

Flow Cytometry

Surface staining was performed in 1× PBS containing 2% fetal calf serum and 2 mM EDTA using anti-human antibodies from BioLegend: CD45 (2D1), CD3 (OKT3), CD4 (OKT4), CD8 (RPA-T8), TIGIT (VSTM3), PD-1 (EH12.2H7), CD107a (H4A3), and CD271/NGFR (ME20.4). Live cells were discriminated by staining with fixable viability dye eFluor 780 (eBioscience). Intracellular proteins were stained for with a cell fixation and cell permeabilization kit (Invitrogen) according to the manufacturer's instructions using antibodies from the following sources: BD Biosciences: TNF (Mab11), IFN-γ (4S.B3), GM-CSF (BVD2-21C11), MIP-1β (D21-1351), IL-21 (3A3-N2.1), GzmB (GB11), and active caspase-3 (C92605); BioLegend: IL-2 (MQH-17H12), IL-17A (BL168), IL-4 (8D4-8), IL-13 (JES10), IL-22 (2412A41), perforin (B-D48), and GzmA (CB9); eBioscience: GzmM (4B2G4); and Beckman Coulter: HIV-1 core antigen (KC57). Flow cytometry data were acquired on a BD LSRFortessa and analyzed using FlowJo software version 10.5.3 (Tree Star).

HIV Viral Load Quantitation

Viral RNA was isolated from plasma (40 μL) using the QiaAmp viral RNA mini kit (QIAGEN). Viral loads were determined using quantitative RT-PCR using the QuantiFast SYBR Green RT-PCR kit (QIAGEN) as previously described.[84] The limit of quantification for this assay is 1.81 log RNA copies/mL plasma.

Plasmid Construction

The amino acid sequences for the HIV-specific CD4-based CAR constructs contained the following intracellular domains: CD3-ζ, 4-1BB/CD3-ζ, CD28/CD3-ζ, CD27/CD3-ζ, OX40/CD3-ζ, and ICOS/CD3-ζ, as described elsewhere.[36] In this study, each CAR was amplified from its original plasmid with the primer 5′-CACGTCCTAGGATGGCCTTACCAGTG-3′ and 5′-GTGGTCG ACTTATGCGCTCCTGCTGAAC-3′ and cloned into pTRPE plasmid using the AvrII and SalI restriction enzyme sites. In this orientation, the CAR sequence is downstream of GFP, and a T2A sequence intervenes to permit expression of both proteins. The amino acid sequence for the C34-CXCR4 construct is described elsewhere.[35] We introduced a single Asp mutation (D97N), which has been shown to impair SDF-1 binding[85] and limit CXCR4 internalization (G.J. Leslie, M. Richardson, J.L.R., and J.A. Hoxie, personal communication). C34-CXCR4 (D97N) was cloned upstream of T2A and NGFR[86] sequences within the pTRPE backbone.

Lentivirus Production and Transfection

Lentivirus particles were generated using expression vectors encoding vesicular stomatitis virus (VSV) glycoprotein (pTRPE pVSVg-g), HIV Rev (pTRPE.Rev), and HIV Gag and Pol (pTRPE g/p). The plasmids were synthesized by DNA 2.0 or ATUM (Newark, CA, USA) and transfected into HEK293T cells with pTRPE transfer vectors described above using Lipofectamine 2000 (Life Technologies) as previously described.[31] Transfected HEK293T cell supernatant was collected 24 and 48 h after transfection, filtered through a sterile 0.45-μm nylon syringe-driven filter, and concentrated by ultracentrifugation at 25,000 rpm for 2.5 h at 4°C. Supernatant was aspirated and the virus pellet was resuspended in 800-μL total volume and stored at −80°C.

Cell Culture and Selection

To manufacture CAR T cells, $CD4^+$ and $CD8^+$ T cells from healthy adult human donors were purified by negative selection using RosetteSep human $CD4^+$ or $CD8^+$ enrichment cocktails (STEMCELL Technologies) according to the manufacturer's instructions. All cells were cultured at 10^6 cells/mL in complete RPMI (expansion medium): RPMI 1640, 2 mM GlutaMax, 25 mM HEPES, and 1% penicillin-streptomycin from Life Technologies, and 10% fetal calf serum (Seradigm). T cell expansion medium was supplemented with 10 ng/mL human IL-7 (R&D Systems) and 5 ng/mL human IL-15 (BioLegend) or 100 U/mL human IL-2 (Clinigen). T cells were stimulated with anti-CD3/CD28 Dynabeads (Life Technologies) at a 3:1 bead-to-cell ratio at 37°C, 5% CO_2 and 95% humidity incubation conditions. Roughly 18 h after stimulation, half of the medium was removed and replaced with 200–300 μL of concentrated lentivirus supernatant for CAR transduction. On day 5, the Dynabeads were removed from cell culture by magnetic separation. Expansion medium was changed every other day throughout cell culture spanning 8–10 days, or as necessary to adjust cell concentration to 0.5×10^6 cells/mL. For *in vivo* studies, on day 7 after activation, C34-CXCR4-transduced T cells were positively selected using CD271/NGFR microbeads (Miltenyi Biotec) according the manufacturer's protocol. Following selection, C34-CXCR4$^+$ T cells were placed in culture with expansion medium for one more day at the adjusted cell concentration prior to infusion into humanized mice.

In Vitro HIV Suppression Assay

Two days after removing anti-CD3/CD28 Dynabeads, activated $CD4^+$ T cells were infected with CCR5-tropic HIV_{BAL} at a multiplicity of infection (MOI) of 1. 24 h later, HIV-challenged $CD4^+$ T cells were washed with complete RPMI and mixed with the indicated type of HIV-specific CAR T cell, or untransduced (UTD) T cell at E:T ratios of 1:25, 1:50, 1:100, and 1:200. Cell mixtures were plated in duplicate and the spread of virus replication was assessed by flow cytometry by sampling 100 μL per well for intracellular staining of HIV-1 core antigen (HIV_{GAG}) at 2, 4, 6, and 8 days after co-culture. Fresh complete RPMI was added to all wells after staining.

CD107a Degranulation and Intracellular Staining Assay

The functionality of CAR T cells was measured *in vitro* after stimulating 2×10^5 CAR T cells or UTD T cells with 2×10^5 wild-type K562s (K.WT) or K562 cells expressing HIV_{YU2} GP160 (K.Env). Anti-CD107a antibody was added at the start of stimulation followed by the addition of 1× brefeldin A and monensin solution (BioLegend) 1 h later. Cells were incubated for a total of 6 h under incubation conditions. Detection of cytokines and cytolytic proteins was assessed by intracellular staining with antibodies specific for human TNF, MIP-1β, IFN-γ, IL-2, GM-CSF, perforin, GzmB, GzmA, GzmM, IL-17A, IL-4, IL-13, IL-21, and IL-22. The percentage of cytokine-positive CAR T cells was calculated by subtracting background production after stimulation with K.WT cells.

HIV-Infected Cell Elimination Assay

This HIV-infected cell elimination assay is based on a previously described protocol.[54] Briefly, HIV-infected $CD4^+$ T cells were prepared as detailed above. When roughly 30% of total cells stained positive for intracellular HIV_{GAG}, the cells were labeled with CellTrace Violet (CTV; Thermo Fisher Scientific) to distinguish target cells from effector cells. CAR or UTD T cells were cultured with CTV^+ target cells at various E:T ratios. After 24 h, target cells were analyzed for the reduction of HIV_{GAG}^+ cells and the induction of active caspase-3 by intracellular staining and flow cytometry. Elimination of HIV-infected cells was calculated by quantitation of live (fixable viability dye eFluor780-negative) $CTV^+CAR^-HIV_{GAG}^+$ cells. The percentage of residual HIV-infected target cells were calculated by dividing the percentage of live $CTV^+CAR^-HIV_{GAG}^+$ target cells at the indicated E:T ratio by the percentage of $CTV^+CAR^-HIV_{GAG}^+$ target cells at an E:T ratio of 0.

Humanized Mice Experiments

NOD (non-obese diabetic)/SCID (severe combined immunodeficiency)/IL-2R$\gamma^{-/-}$ (NSG) mice (The Jackson Laboratory) were housed in a pathogen-free facility at the University of Pennsylvania. Mice were maintained in microisolator cages and fed autoclaved food and water. Humanized mice were generated essentially as previously described.[36] NSG mice were infused with 5×10^6 healthy donor PBMCs depleted of $CD8^+$ T cells using CD8 microbeads (Miltenyi Biotec) following the manufacturer's protocol. Two weeks later mice initiated daily ART consisting of 1 mg/kg EFdA (4′-ethynyl-2-fluoro-2′-deoxyadenosine, LeadGen Labs). After 1 day, mice were infused with 10^6 autologous $HIV_{GAG}{}^+CD4^+$ T cells and mixed with 9×10^6 uninfected $CD4^+$ T cells. Prior to infusion, $CD4^+$ T cells were *in vitro* infected with HIV_{BAL} (MOI of 1) as described above and expanded in culture for 4 more days, where during the last 2 days 1 μM EFdA supplemented the expansion medium. Mice were bled 1 or 2 days after the infusion of HIV-infected cells and then allocated into groups (n = 6–8) based on the concentration of peripheral blood $CD4^+$ T cells. Three days after the infusion of HIV-infected cells, mice were infused with HIV-resistant (>90% C34-$CXCR4^+$) CAR T cells as described above and ART was interrupted. For the study described in Figure 4, each group of humanized mice received an infusion of 2.5×10^6 CAR_4 T cells expressing one of the six ICDs, or inactive control CAR_4 T cells expressing a truncated CD3ζ ICD. For the study described in Figure 6, mouse groups received an infusion product consisting of either (CD28ζ) CAR_8 T cells (2.5×10^6 CAR^+ cells), a 1:1 ratio of CAR_4 (4-1BBζ) and CAR_8 (CD28ζ) T cells (1.25×10^6 CAR^+ cells/cell type), or 1:1 ratio of inactive control CAR_4 and CAR_8 T cells (1.25×10^6 CAR^+ cells/cell type) expressing the CD3Δζ ICD. In another study shown in Figure S12, we recapitulated this experimental design, but the CAR_8 T cells expressed the 4-1BBζ ICD instead of the CD28ζ ICD. For all studies the mice were bled weekly via retro-orbital puncture following the $CD4^+$ T cell engraftment bleed until the study endpoint.

Statistical Analysis

All statistical analysis was performed using GraphPad Prism version 7 (GraphPad, San Diego, CA, USA). A comparison of means from unmatched samples was performed using the non-parametric Wilcoxon rank sum test or Kruskal-Wallis test followed by Dunn's test for multiple comparisons. Comparison of means from matched samples was performed using paired Student's t test or Wilcoxon a matched-pairs signed-rank test. Bivariate correlations were performed using Spearman's rank correlation. Area under the curve calculations were performed using either cell concentration normalized to 1 μL of blood or frequency of HIV-infected cells.

SUPPLEMENTAL INFORMATION

Supplemental Information can be found online at https://doi.org/10.1016/j.ymthe.2020.05.012.

AUTHOR CONTRIBUTIONS

C.R.M., K.G., R.S.L., D.L.D., J.P.M., X.S., and J.A.G. contributed to the acquisition and analysis of data; C.R.M. and J.L.R. conceived and designed the project, contributed to the interpretation of the data, and drafted the manuscript.

CONFLICTS OF INTEREST

R.S.L. and J.L.R. have filed a patent describing the construction of these HIV-specific CARs. J.L.R. co-founded Tmunity Therapeutics that has the rights to license the technology described herein. J.L.R. holds an equity interest in Tmunity. The remaining authors declare no competing interests.

ACKNOWLEDGMENTS

We kindly thank Christoph Ellebrecht and Aimee Payne (University of Pennsylvania) for providing the K562 cell line transduced with HIV_{YU2} GFP160, and George Leslie, Max Richardson, and James Hoxie (University of Pennsylvania) for providing the C34-CXCR4 (D97N) construct used in this study. This work was supported by U19AI117950 (to J.L.R.) and UM1AI126620 (to J.L.R.) which were co-funded by the NIH, NIAID, NIMH, NINDS, and NIDA. C.R.M. is supported by a T32 grant (AI00763). We also thank the Penn Center for AIDS Research (P30-AI045008) for providing human T cells, and helpful comments and suggestions from the Riley laboratory.

REFERENCES

1. Okoye, A.A., and Picker, L.J. (2013). $CD4^+$ T-cell depletion in HIV infection: mechanisms of immunological failure. Immunol. Rev. *254*, 54–64.

2. Douek, D.C., Brenchley, J.M., Betts, M.R., Ambrozak, D.R., Hill, B.J., Okamoto, Y., Casazza, J.P., Kuruppu, J., Kunstman, K., Wolinsky, S., et al. (2002). HIV preferentially infects HIV-specific $CD4^+$ T cells. Nature *417*, 95–98.

3. Younes, S.A., Yassine-Diab, B., Dumont, A.R., Boulassel, M.R., Grossman, Z., Routy, J.P., and Sekaly, R.P. (2003). HIV-1 viremia prevents the establishment of interleukin 2-producing HIV-specific memory $CD4^+$ T cells endowed with proliferative capacity. J. Exp. Med. *198*, 1909–1922.

4. Palmer, B.E., Boritz, E., Blyveis, N., and Wilson, C.C. (2002). Discordance between frequency of human immunodeficiency virus type 1 (HIV-1)-specific gamma interferon-producing $CD4^+$ T cells and HIV-1-specific lymphoproliferation in HIV-1-infected subjects with active viral replication. J. Virol. *76*, 5925–5936.

5. Betts, M.R., Ambrozak, D.R., Douek, D.C., Bonhoeffer, S., Brenchley, J.M., Casazza, J.P., Koup, R.A., and Picker, L.J. (2001). Analysis of total human immunodeficiency virus (HIV)-specific $CD4^+$ and $CD8^+$ T-cell responses: relationship to viral load in untreated HIV infection. J. Virol. *75*, 11983–11991.

6. Matloubian, M., Concepcion, R.J., and Ahmed, R. (1994). $CD4^+$ T cells are required to sustain $CD8^+$ cytotoxic T-cell responses during chronic viral infection. J. Virol. *68*, 8056–8063.

7. Janssen, E.M., Lemmens, E.E., Wolfe, T., Christen, U., von Herrath, M.G., and Schoenberger, S.P. (2003). $CD4^+$ T cells are required for secondary expansion and memory in $CD8^+$ T lymphocytes. Nature *421*, 852–856.

8. Shedlock, D.J., and Shen, H. (2003). Requirement for CD4 T cell help in generating functional CD8 T cell memory. Science *300*, 337–339.

9. Zajac, A.J., Blattman, J.N., Murali-Krishna, K., Sourdive, D.J., Suresh, M., Altman, J.D., and Ahmed, R. (1998). Viral immune evasion due to persistence of activated T cells without effector function. J. Exp. Med. *188*, 2205–2213.

10. Cubas, R.A., Mudd, J.C., Savoye, A.L., Perreau, M., van Grevenynghe, J., Metcalf, T., Connick, E., Meditz, A., Freeman, G.J., Abesada-Terk, G., Jr., et al. (2013). Inadequate T follicular cell help impairs B cell immunity during HIV infection. Nat. Med. *19*, 494–499.

11. Pallikkuth, S., de Armas, L., Rinaldi, S., and Pahwa, S. (2017). T follicular helper cells and B Cell dysfunction in aging and HIV-1 infection. Front. Immunol. *8*, 1380.

12. Casazza, J.P., Betts, M.R., Price, D.A., Precopio, M.L., Ruff, L.E., Brenchley, J.M., Hill, B.J., Roederer, M., Douek, D.C., and Koup, R.A. (2006). Acquisition of direct antiviral effector functions by CMV-specific $CD4^+$ T lymphocytes with cellular maturation. J. Exp. Med. *203*, 2865–2877.

13. Brown, D.M., Dilzer, A.M., Meents, D.L., and Swain, S.L. (2006). CD4 T cell-mediated protection from lethal influenza: perforin and antibody-mediated mechanisms give a one-two punch. J. Immunol. *177*, 2888–2898.

14. Pike, R., Filby, A., Ploquin, M.J., Eksmond, U., Marques, R., Antunes, I., Hasenkrug, K., and Kassiotis, G. (2009). Race between retroviral spread and $CD4^+$ T-cell response determines the outcome of acute Friend virus infection. J. Virol. *83*, 11211–11222.

15. Norris, P.J., Moffett, H.F., Yang, O.O., Kaufmann, D.E., Clark, M.J., Addo, M.M., and Rosenberg, E.S. (2004). Beyond help: direct effector functions of human immunodeficiency virus type 1-specific $CD4^+$ T cells. J. Virol. *78*, 8844–8851.

16. Soghoian, D.Z., Jessen, H., Flanders, M., Sierra-Davidson, K., Cutler, S., Pertel, T., Ranasinghe, S., Lindqvist, M., Davis, I., Lane, K., et al. (2012). HIV-specific cytolytic CD4 T cell responses during acute HIV infection predict disease outcome. Sci. Transl. Med. *4*, 123ra25.

17. Guedan, S., Chen, X., Madar, A., Carpenito, C., McGettigan, S.E., Frigault, M.J., Lee, J., Posey, A.D., Jr., Scholler, J., Scholler, N., et al. (2014). ICOS-based chimeric antigen receptors program bipolar T_H17/T_H1 cells. Blood *124*, 1070–1080.

18. Wang, D., Aguilar, B., Starr, R., Alizadeh, D., Brito, A., Sarkissian, A., Ostberg, J.R., Forman, S.J., and Brown, C.E. (2018). Glioblastoma-targeted $CD4^+$ CAR T cells mediate superior antitumor activity. JCI Insight *3*, 99048.

19. Gacerez, A.T., and Sentman, C.L. (2018). T-bet promotes potent antitumor activity of $CD4^+$ CAR T cells. Cancer Gene Ther. *25*, 117–128.

20. Gross, G., Waks, T., and Eshhar, Z. (1989). Expression of immunoglobulin-T-cell receptor chimeric molecules as functional receptors with antibody-type specificity. Proc. Natl. Acad. Sci. USA *86*, 10024–10028.

21. van der Stegen, S.J., Hamieh, M., and Sadelain, M. (2015). The pharmacology of second-generation chimeric antigen receptors. Nat. Rev. Drug Discov. *14*, 499–509.

22. Weinkove, R., George, P., Dasyam, N., and McLellan, A.D. (2019). Selecting costimulatory domains for chimeric antigen receptors: functional and clinical considerations. Clin. Transl. Immunology *8*, e1049.

23. Salter, A.I., Ivey, R.G., Kennedy, J.J., Voillet, V., Rajan, A., Alderman, E.J., Voytovich, U.J., Lin, C., Sommermeyer, D., Liu, L., et al. (2018). Phosphoproteomic analysis of chimeric antigen receptor signaling reveals kinetic and quantitative differences that affect cell function. Sci. Signal. *11*, eaat6753.

24. Zhao, Z., Condomines, M., van der Stegen, S.J.C., Perna, F., Kloss, C.C., Gunset, G., Plotkin, J., and Sadelain, M. (2015). Structural design of engineered costimulation determines tumor rejection kinetics and persistence of CAR T cells. Cancer Cell *28*, 415–428.

25. Kawalekar, O.U., O'Connor, R.S., Fraietta, J.A., Guo, L., McGettigan, S.E., Posey, A.D., Jr., Patel, P.R., Guedan, S., Scholler, J., Keith, B., et al. (2016). Distinct signaling of coreceptors regulates specific metabolism pathways and impacts memory development in CAR T cells. Immunity *44*, 380–390.

26. Milone, M.C., Fish, J.D., Carpenito, C., Carroll, R.G., Binder, G.K., Teachey, D., Samanta, M., Lakhal, M., Gloss, B., Danet-Desnoyers, G., et al. (2009). Chimeric receptors containing CD137 signal transduction domains mediate enhanced survival of T cells and increased antileukemic efficacy in vivo. Mol. Ther. *17*, 1453–1464.

27. Leibman, R.S., and Riley, J.L. (2015). Engineering T cells to functionally cure HIV-1 infection. Mol. Ther. *23*, 1149–1159.

28. Perez, E.E., Wang, J., Miller, J.C., Jouvenot, Y., Kim, K.A., Liu, O., Wang, N., Lee, G., Bartsevich, V.V., Lee, Y.L., et al. (2008). Establishment of HIV-1 resistance in $CD4^+$ T cells by genome editing using zinc-finger nucleases. Nat. Biotechnol. *26*, 808–816.

29. Wilen, C.B., Wang, J., Tilton, J.C., Miller, J.C., Kim, K.A., Rebar, E.J., Sherrill-Mix, S.A., Patro, S.C., Secreto, A.J., Jordan, A.P., et al. (2011). Engineering HIV-resistant human $CD4^+$ T cells with CXCR4-specific zinc-finger nucleases. PLoS Pathog. *7*, e1002020.

30. Vakulskas, C.A., Dever, D.P., Rettig, G.R., Turk, R., Jacobi, A.M., Collingwood, M.A., Bode, N.M., McNeill, M.S., Yan, S., Camarena, J., et al. (2018). A high-fidelity Cas9 mutant delivered as a ribonucleoprotein complex enables efficient gene editing in human hematopoietic stem and progenitor cells. Nat. Med. *24*, 1216–1224.

31. Richardson, M.W., Carroll, R.G., Stremlau, M., Korokhov, N., Humeau, L.M., Silvestri, G., Sodroski, J., and Riley, J.L. (2008). Mode of transmission affects the sensitivity of human immunodeficiency virus type 1 to restriction by rhesus TRIM5α. J. Virol. *82*, 11117–11128.

32. Richardson, M.W., Guo, L., Xin, F., Yang, X., and Riley, J.L. (2014). Stabilized human TRIM5α protects human T cells from HIV-1 infection. Mol. Ther. *22*, 1084–1095.

33. Perez, E.E., Riley, J.L., Carroll, R.G., von Laer, D., and June, C.H. (2005). Suppression of HIV-1 infection in primary CD4 T cells transduced with a self-inactivating lentiviral vector encoding a membrane expressed gp41-derived fusion inhibitor. Clin. Immunol. *115*, 26–32.

34. Hildinger, M., Dittmar, M.T., Schult-Dietrich, P., Fehse, B., Schnierle, B.S., Thaler, S., Stiegler, G., Welker, R., and von Laer, D. (2001). Membrane-anchored peptide inhibits human immunodeficiency virus entry. J. Virol. *75*, 3038–3042.

35. Leslie, G.J., Wang, J., Richardson, M.W., Haggarty, B.S., Hua, K.L., Duong, J., Secreto, A.J., Jordon, A.P., Romano, J., Kumar, K.E., et al. (2016). Potent and broad inhibition of HIV-1 by a peptide from the gp41 heptad repeat-2 domain conjugated to the CXCR4 amino terminus. PLoS Pathog. *12*, e1005983.

36. Leibman, R.S., Richardson, M.W., Ellebrecht, C.T., Maldini, C.R., Glover, J.A., Secreto, A.J., Kulikovskaya, I., Lacey, S.F., Akkina, S.R., Yi, Y., et al. (2017). Supraphysiologic control over HIV-1 replication mediated by CD8 T cells expressing a re-engineered CD4-based chimeric antigen receptor. PLoS Pathog. *13*, e1006613.

37. Walker, R.E., Bechtel, C.M., Natarajan, V., Baseler, M., Hege, K.M., Metcalf, J.A., Stevens, R., Hazen, A., Blaese, R.M., Chen, C.C., et al. (2000). Long-term in vivo survival of receptor-modified syngeneic T cells in patients with human immunodeficiency virus infection. Blood *96*, 467–474.

38. Deeks, S.G., Wagner, B., Anton, P.A., Mitsuyasu, R.T., Scadden, D.T., Huang, C., Macken, C., Richman, D.D., Christopherson, C., June, C.H., et al. (2002). A phase II randomized study of HIV-specific T-cell gene therapy in subjects with undetectable plasma viremia on combination antiretroviral therapy. Mol. Ther. *5*, 788–797.

39. Mitsuyasu, R.T., Anton, P.A., Deeks, S.G., Scadden, D.T., Connick, E., Downs, M.T., Bakker, A., Roberts, M.R., June, C.H., Jalali, S., et al. (2000). Prolonged survival and tissue trafficking following adoptive transfer of CD4ζ gene-modified autologous $CD4^+$ and $CD8^+$ T cells in human immunodeficiency virus-infected subjects. Blood *96*, 785–793.

40. Lynch, R.M., Wong, P., Tran, L., O'Dell, S., Nason, M.C., Li, Y., Wu, X., and Mascola, J.R. (2015). HIV-1 fitness cost associated with escape from the VRC01 class of CD4 binding site neutralizing antibodies. J. Virol. *89*, 4201–4213.

41. Zhen, A., Peterson, C.W., Carrillo, M.A., Reddy, S.S., Youn, C.S., Lam, B.B., Chang, N.Y., Martin, H.A., Rick, J.W., Kim, J., et al. (2017). Long-term persistence and function of hematopoietic stem cell-derived chimeric antigen receptor T cells in a nonhuman primate model of HIV/AIDS. PLoS Pathog. *13*, e1006753.

42. Zhen, A., Kamata, M., Rezek, V., Rick, J., Levin, B., Kasparian, S., Chen, I.S., Yang, O.O., Zack, J.A., and Kitchen, S.G. (2015). HIV-specific immunity derived from chimeric antigen receptor-engineered stem cells. Mol. Ther. *23*, 1358–1367.

43. Hale, M., Mesojednik, T., Romano Ibarra, G.S., Sahni, J., Bernard, A., Sommer, K., Scharenberg, A.M., Rawlings, D.J., and Wagner, T.A. (2017). Engineering HIV-resistant, anti-HIV chimeric antigen receptor T cells. Mol. Ther. *25*, 570–579.

44. Ali, A., Kitchen, S.G., Chen, I.S.Y., Ng, H.L., Zack, J.A., and Yang, O.O. (2016). HIV-1-specific chimeric antigen receptors based on broadly neutralizing antibodies. J. Virol. *90*, 6999–7006.

45. Herzig, E., Kim, K.C., Packard, T.A., Vardi, N., Schwarzer, R., Gramatica, A., Deeks, S.G., Williams, S.R., Landgraf, K., Killeen, N., et al. (2019). Attacking latent HIV with convertible CAR-T cells, a highly adaptable killing platform. Cell *179*, 880–894.e10.

46. Ghanem, M.H., Bolivar-Wagers, S., Dey, B., Hajduczki, A., Vargas-Inchaustegui, D.A., Danielson, D.T., Bundoc, V., Liu, L., and Berger, E.A. (2018). Bispecific chimeric antigen receptors targeting the CD4 binding site and high-mannose Glycans of gp120 optimized for anti-human immunodeficiency virus potency and breadth with minimal immunogenicity. Cytotherapy *20*, 407–419.

47. Guedan, S., Posey, A.D., Jr., Shaw, C., Wing, A., Da, T., Patel, P.R., McGettigan, S.E., Casado-Medrano, V., Kawalekar, O.U., Uribe-Herranz, M., et al. (2018). Enhancing CAR T cell persistence through ICOS and 4-1BB costimulation. JCI Insight *3*, 96976.

48. Zhang, H., Snyder, K.M., Suhoski, M.M., Maus, M.V., Kapoor, V., June, C.H., and Mackall, C.L. (2007). 4-1BB is superior to CD28 costimulation for generating $CD8^+$ cytotoxic lymphocytes for adoptive immunotherapy. J. Immunol. *179*, 4910–4918.

49. Guidotti, L.G., and Chisari, F.V. (2001). Noncytolytic control of viral infections by the innate and adaptive immune response. Annu. Rev. Immunol. *19*, 65–91.

50. Jellison, E.R., Kim, S.K., and Welsh, R.M. (2005). Cutting edge: MHC class II-restricted killing in vivo during viral infection. J. Immunol. *174*, 614–618.

51. Brien, J.D., Uhrlaub, J.L., and Nikolich-Zugich, J. (2008). West Nile virus-specific CD4 T cells exhibit direct antiviral cytokine secretion and cytotoxicity and are sufficient for antiviral protection. J. Immunol. *181*, 8568–8575.

52. Iwashiro, M., Peterson, K., Messer, R.J., Stromnes, I.M., and Hasenkrug, K.J. (2001). $CD4(^+)$ T cells and gamma interferon in the long-term control of persistent friend retrovirus infection. J. Virol. *75*, 52–60.

53. Liadi, I., Singh, H., Romain, G., Rey-Villamizar, N., Merouane, A., Adolacion, J.R., Kebriaei, P., Huls, H., Qiu, P., Roysam, B., et al. (2015). Individual Motile $CD4(^+)$ T Cells Can Participate in Efficient Multikilling through Conjugation to Multiple Tumor Cells. Cancer Immunol. Res. *3*, 473–482.

54. Clayton, K.L., Collins, D.R., Lengieza, J., Ghebremichael, M., Dotiwala, F., Lieberman, J., and Walker, B.D. (2018). Resistance of HIV-infected macrophages to $CD8^+$ T lymphocyte-mediated killing drives activation of the immune system. Nat. Immunol. *19*, 475–486.

55. Slee, E.A., Adrain, C., and Martin, S.J. (2001). Executioner caspase-3, -6, and -7 perform distinct, non-redundant roles during the demolition phase of apoptosis. J. Biol. Chem. *276*, 7320–7326.

56. Darmon, A.J., Nicholson, D.W., and Bleackley, R.C. (1995). Activation of the apoptotic protease CPP32 by cytotoxic T-cell-derived granzyme B. Nature *377*, 446–448.

57. Betts, M.R., Brenchley, J.M., Price, D.A., De Rosa, S.C., Douek, D.C., Roederer, M., and Koup, R.A. (2003). Sensitive and viable identification of antigen-specific $CD8^+$ T cells by a flow cytometric assay for degranulation. J. Immunol. Methods *281*, 65–78.

58. Picchio, G.R., Gulizia, R.J., Wehrly, K., Chesebro, B., and Mosier, D.E. (1998). The cell tropism of human immunodeficiency virus type 1 determines the kinetics of plasma viremia in SCID mice reconstituted with human peripheral blood leukocytes. J. Virol. *72*, 2002–2009.

59. Gulizia, R.J., Collman, R.G., Levy, J.A., Trono, D., and Mosier, D.E. (1997). Deletion of nef slows but does not prevent CD4-positive T-cell depletion in human immunodeficiency virus type 1-infected human-PBL-SCID mice. J. Virol. *71*, 4161–4164.

60. Zhou, Y., Maldini, C.R., Jadlowsky, J., and Riley, J.L. (2020). Challenges and opportunities of using adoptive T cell therapy as part of an HIV cure strategy. J. Infect. Dis. https://doi.org/10.1093/infdis/jiaa223.

61. Riley, J.L., and June, C.H. (2005). The CD28 family: a T-cell rheostat for therapeutic control of T-cell activation. Blood *105*, 13–21.

62. DuPage, M., and Bluestone, J.A. (2016). Harnessing the plasticity of $CD4^+$ T cells to treat immune-mediated disease. Nat. Rev. Immunol. *16*, 149–163.

63. Maude, S.L., Laetsch, T.W., Buechner, J., Rives, S., Boyer, M., Bittencourt, H., Bader, P., Verneris, M.R., Stefanski, H.E., Myers, G.D., et al. (2018). Tisagenlecleucel in children and young adults with B-cell lymphoblastic leukemia. N. Engl. J. Med. *378*, 439–448.

64. Maude, S.L., Frey, N., Shaw, P.A., Aplenc, R., Barrett, D.M., Bunin, N.J., Chew, A., Gonzalez, V.E., Zheng, Z., Lacey, S.F., et al. (2014). Chimeric antigen receptor T cells for sustained remissions in leukemia. N. Engl. J. Med. *371*, 1507–1517.

65. Turtle, C.J., Hanafi, L.A., Berger, C., Gooley, T.A., Cherian, S., Hudecek, M., Sommermeyer, D., Melville, K., Pender, B., Budiarto, T.M., et al. (2016). CD19 CAR-T cells of defined $CD4^+$:$CD8^+$ composition in adult B cell ALL patients. J. Clin. Invest. *126*, 2123–2138.

66. Song, D.G., Ye, Q., Poussin, M., Harms, G.M., Figini, M., and Powell, D.J., Jr. (2012). CD27 costimulation augments the survival and antitumor activity of redirected human T cells in vivo. Blood *119*, 696–706.

67. Levine, B.L., Bernstein, W.B., Connors, M., Craighead, N., Lindsten, T., Thompson, C.B., and June, C.H. (1997). Effects of CD28 costimulation on long-term proliferation of $CD4^+$ T cells in the absence of exogenous feeder cells. J. Immunol. *159*, 5921–5930.

68. Savoldo, B., Ramos, C.A., Liu, E., Mims, M.P., Keating, M.J., Carrum, G., Kamble, R.T., Bollard, C.M., Gee, A.P., Mei, Z., et al. (2011). CD28 costimulation improves expansion and persistence of chimeric antigen receptor-modified T cells in lymphoma patients. J. Clin. Invest. *121*, 1822–1826.

69. Finzi, D., Blankson, J., Siliciano, J.D., Margolick, J.B., Chadwick, K., Pierson, T., Smith, K., Lisziewicz, J., Lori, F., Flexner, C., et al. (1999). Latent infection of $CD4^+$ T cells provides a mechanism for lifelong persistence of HIV-1, even in patients on effective combination therapy. Nat. Med. *5*, 512–517.

70. Siliciano, J.D., Kajdas, J., Finzi, D., Quinn, T.C., Chadwick, K., Margolick, J.B., Kovacs, C., Gange, S.J., and Siliciano, R.F. (2003). Long-term follow-up studies confirm the stability of the latent reservoir for HIV-1 in resting $CD4^+$ T cells. Nat. Med. *9*, 727–728.

71. Maldini, C.R., Ellis, G.I., and Riley, J.L. (2018). CAR T cells for infection, autoimmunity and allotransplantation. Nat. Rev. Immunol. *18*, 605–616.

72. Golovina, T.N., Mikheeva, T., Suhoski, M.M., Aqui, N.A., Tai, V.C., Shan, X., Liu, R., Balcarcel, R.R., Fisher, N., Levine, B.L., et al. (2008). CD28 costimulation is essential for human T regulatory expansion and function. J. Immunol. *181*, 2855–2868.

73. King, M.A., Covassin, L., Brehm, M.A., Racki, W., Pearson, T., Leif, J., Laning, J., Fodor, W., Foreman, O., Burzenski, L., et al. (2009). Human peripheral blood leucocyte non-obese diabetic-severe combined immunodeficiency interleukin-2 receptor gamma chain gene mouse model of xenogeneic graft-versus-host-like disease and the role of host major histocompatibility complex. Clin. Exp. Immunol. *157*, 104–118.

74. Finzi, D., Hermankova, M., Pierson, T., Carruth, L.M., Buck, C., Chaisson, R.E., Quinn, T.C., Chadwick, K., Margolick, J., Brookmeyer, R., et al. (1997). Identification of a reservoir for HIV-1 in patients on highly active antiretroviral therapy. Science *278*, 1295–1300.

75. Denton, P.W., Olesen, R., Choudhary, S.K., Archin, N.M., Wahl, A., Swanson, M.D., Chateau, M., Nochi, T., Krisko, J.F., Spagnuolo, R.A., et al. (2012). Generation of HIV latency in humanized BLT mice. J. Virol. *86*, 630–634.

76. Marsden, M.D., Kovochich, M., Suree, N., Shimizu, S., Mehta, R., Cortado, R., Bristol, G., An, D.S., and Zack, J.A. (2012). HIV latency in the humanized BLT mouse. J. Virol. *86*, 339–347.

77. Chevalier, M.F., Jülg, B., Pyo, A., Flanders, M., Ranasinghe, S., Soghoian, D.Z., Kwon, D.S., Rychert, J., Lian, J., Muller, M.I., et al. (2011). HIV-1-specific interleukin-21^+ $CD4^+$ T cell responses contribute to durable viral control through the modulation of HIV-specific $CD8^+$ T cell function. J. Virol. *85*, 733–741.

78. Blattman, J.N., Grayson, J.M., Wherry, E.J., Kaech, S.M., Smith, K.A., and Ahmed, R. (2003). Therapeutic use of IL-2 to enhance antiviral T-cell responses in vivo. Nat. Med. *9*, 540–547.

79. Elsaesser, H., Sauer, K., and Brooks, D.G. (2009). IL-21 is required to control chronic viral infection. Science *324*, 1569–1572.

80. Johnson, S., Eller, M., Teigler, J.E., Maloveste, S.M., Schultz, B.T., Soghoian, D.Z., Lu, R., Oster, A.F., Chenine, A.L., Alter, G., et al. (2015). Cooperativity of HIV-specific cytolytic CD4 T cells and CD8 T cells in control of HIV viremia. J. Virol. *89*, 7494–7505.

81. Lichterfeld, M., Kaufmann, D.E., Yu, X.G., Mui, S.K., Addo, M.M., Johnston, M.N., Cohen, D., Robbins, G.K., Pae, E., Alter, G., et al. (2004). Loss of HIV-1-specific $CD8^+$ T cell proliferation after acute HIV-1 infection and restoration by vaccine-induced HIV-1-specific $CD4^+$ T cells. J. Exp. Med. *200*, 701–712.

82. Yang, Y., Kohler, M.E., Chien, C.D., Sauter, C.T., Jacoby, E., Yan, C., Hu, Y., Wanhainen, K., Qin, H., and Fry, T.J. (2017). TCR engagement negatively affects

CD8 but not CD4 CAR T cell expansion and leukemic clearance. Sci. Transl. Med. *9*, eaag1209.

83. Sommermeyer, D., Hudecek, M., Kosasih, P.L., Gogishvili, T., Maloney, D.G., Turtle, C.J., and Riddell, S.R. (2016). Chimeric antigen receptor-modified T cells derived from defined $CD8^+$ and $CD4^+$ subsets confer superior antitumor reactivity in vivo. Leukemia *30*, 492–500.

84. Boutwell, C.L., Rowley, C.F., and Essex, M. (2009). Reduced viral replication capacity of human immunodeficiency virus type 1 subtype C caused by cytotoxic-T-lymphocyte escape mutations in HLA-B57 epitopes of capsid protein. J. Virol. *83*, 2460–2468.

85. Brelot, A., Heveker, N., Montes, M., and Alizon, M. (2000). Identification of residues of CXCR4 critical for human immunodeficiency virus coreceptor and chemokine receptor activities. J. Biol. Chem. *275*, 23736–23744.

86. Casucci, M., Falcone, L., Camisa, B., Norelli, M., Porcellini, S., Stornaiuolo, A., Ciceri, F., Traversari, C., Bordignon, C., Bonini, C., and Bondanza, A. (2018). Extracellular NGFR spacers allow efficient tracking and enrichment of fully functional CAR-T cells co-expressing a suicide gene. Front. Immunol. *9*, 507.

YMTHE, Volume *28*

Supplemental Information

HIV-Resistant and HIV-Specific CAR-Modified CD4$^+$ T Cells Mitigate HIV Disease Progression and Confer CD4$^+$ T Cell Help *In Vivo*

Colby R. Maldini, Kevin Gayout, Rachel S. Leibman, Derrick L. Dopkin, Joshua P. Mills, Xiaochuan Shan, Joshua A. Glover, and James L. Riley

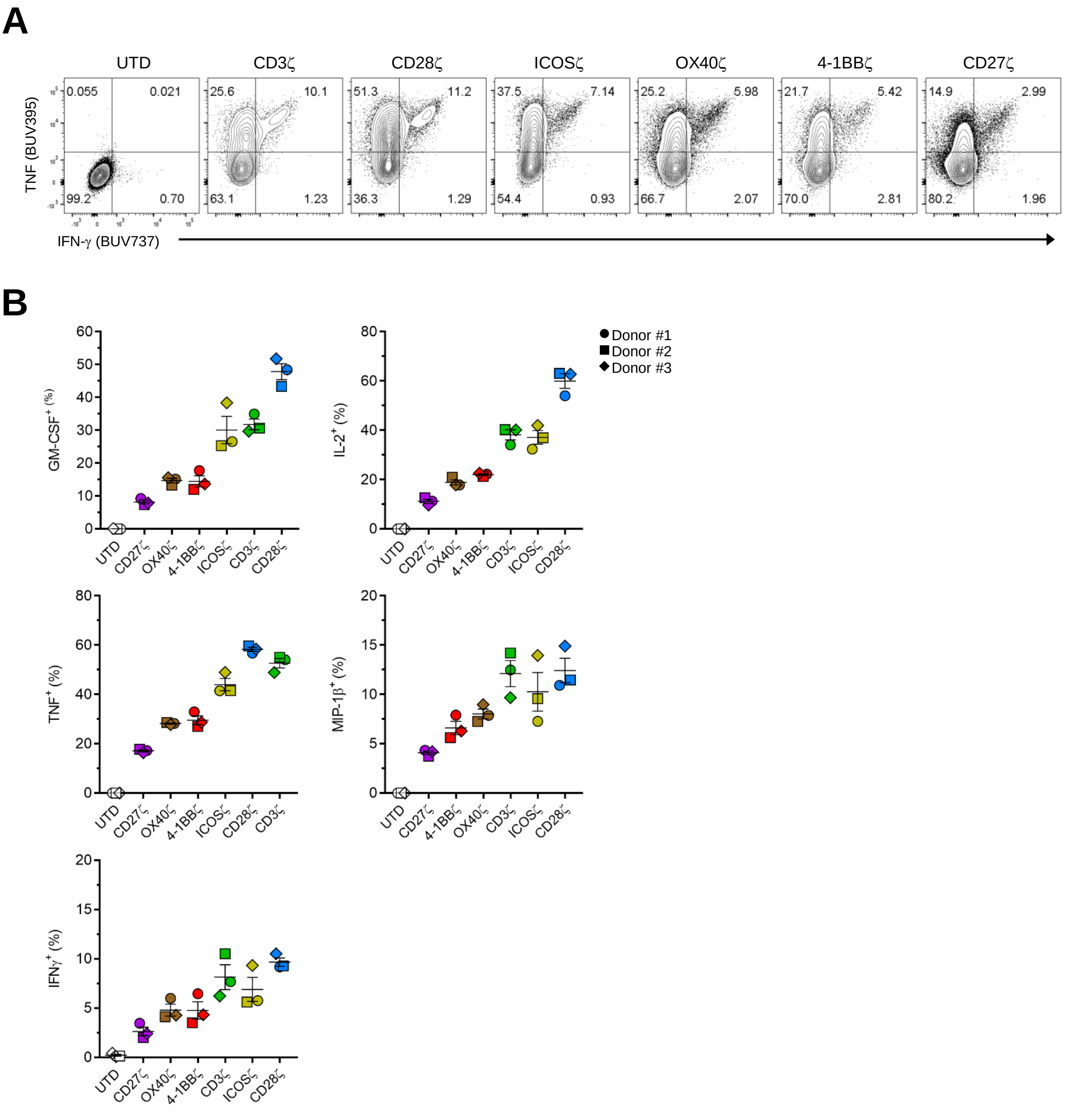

Figure S1. Distinct HIV-specific CAR$_4$ T cell types differentially express cytokines after *in vitro* antigen-specific stimulation. **(A)** FACS plots indicating the frequency of cytokine expressing HIV-specific CAR$_4$ T cells after *in vitro* stimulation with K.Env cells 10 days after activation with αCD3/CD28 Dynabeads. Data are representative of 3 donors. **(B)** Summary data indicating the frequency of cytokine expressing CAR$_4$ T cells. Each symbol represents a distinct donor. The percentage of cytokine expressing CAR$_4$ T cells was calculated by subtracting background production after stimulation with wild-type K562 cells. Lines indicate mean and error bars show ± SEM.

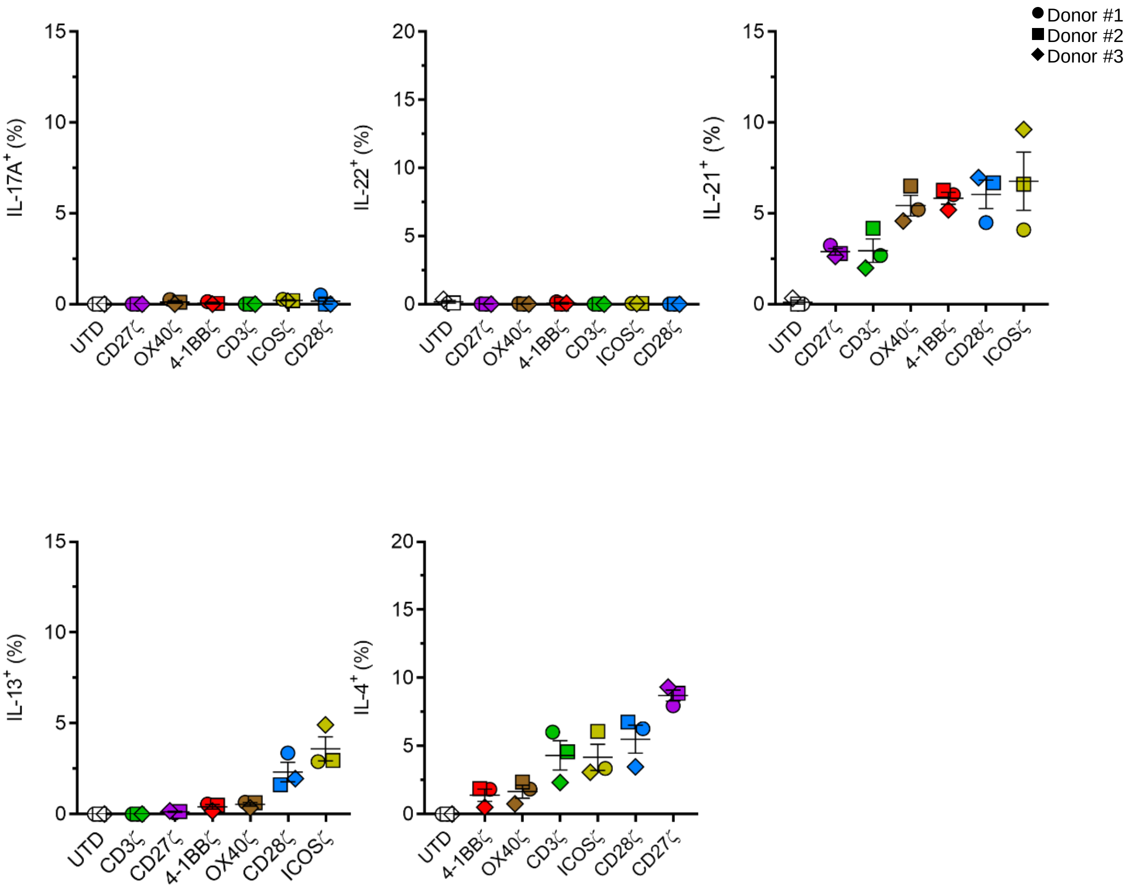

Figure S2. Distinct HIV-specific CAR$_4$ T cell types express low levels of Th17- and Th2-associated cytokines. Summary data indicating the frequency of cytokine expressing CAR$_4$ T cells after *in vitro* stimulation with K.Env 10 days after activation with αCD3/CD28 Dynabeads. Each symbol represents a distinct donor. The percentage of cytokine expressing CAR$_4$ T cells was calculated by subtracting background production after stimulation with wild-type K562 cells. Lines indicate mean and error bars show ± SEM.

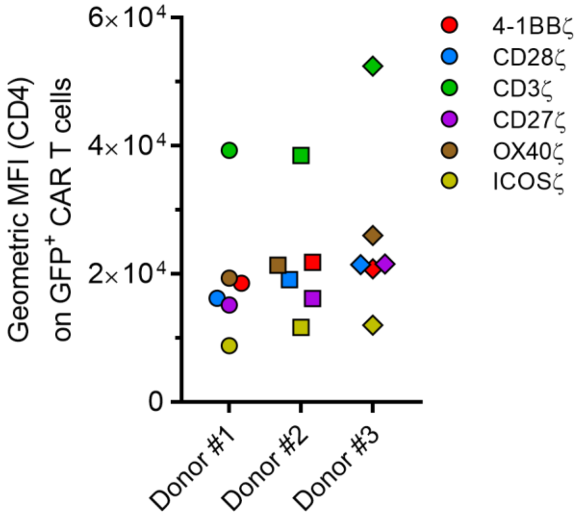

Figure S3. CD4-based CAR containing the CD3ζ intracellular domain exhibits the greatest surface expression on $CD4^+$ T cells. Summary data indicates the geometric mean fluorescence intensity (MFI) determined by flow cytometry of CD4 on GFP^+ CAR_4 T cells 10 days after activation with αCD3/CD28 Dynabeads.

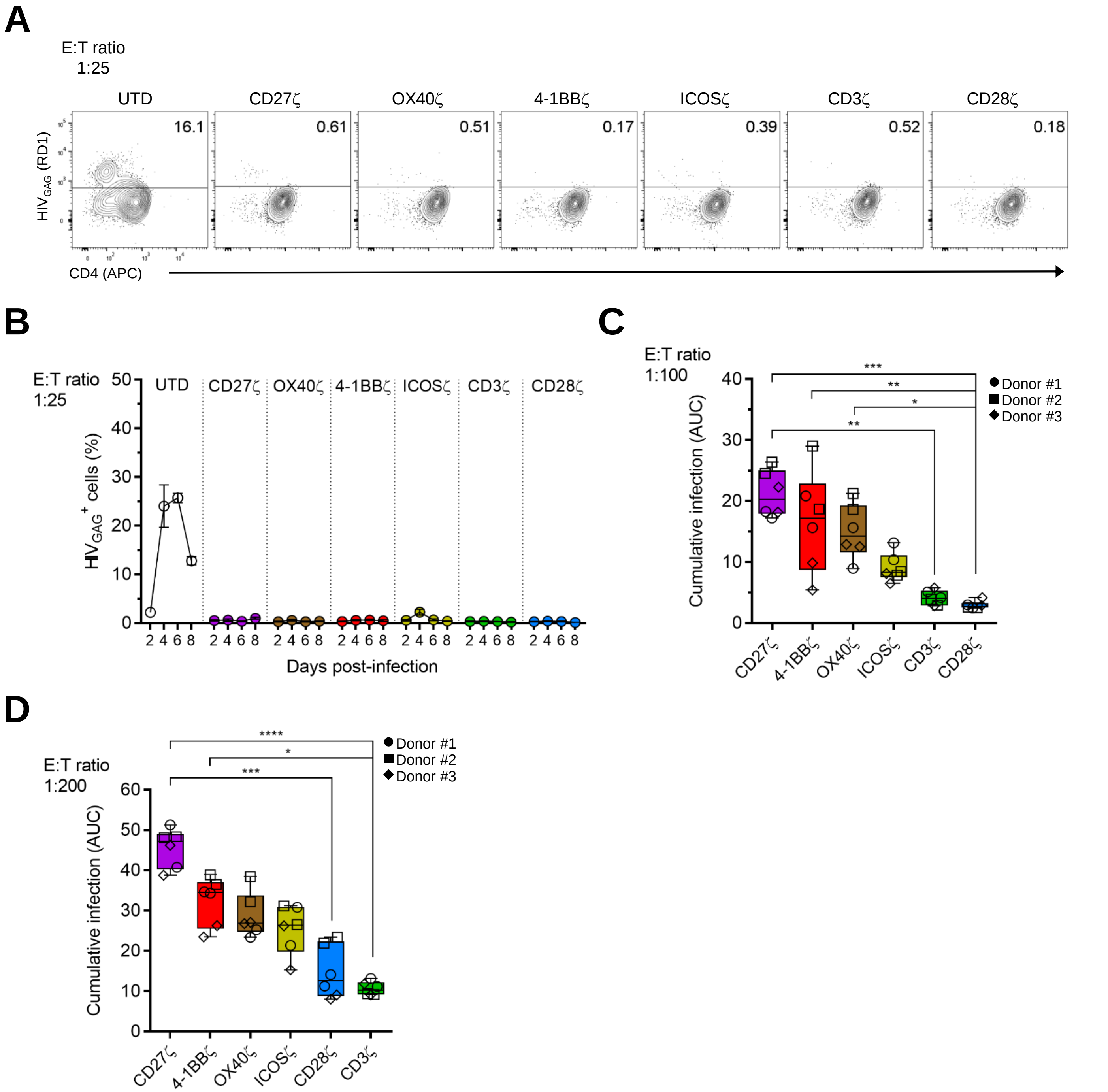

Figure S4. Intracellular domains modulate CAR_4 T cell-mediated control of *in vitro* HIV replication. Each HIV-specific CAR_4 T cell type and UTD_4 T cells were mixed separately with HIV_{BAL}-challenged $CD4^+$ T cells at the indicated effector-to-target (E:T) ratios, and the level of virus spread was monitored by intracellular staining and flow cytometry for HIV_{GAG} antigen on 2, 4, 6 and 8 days after co-culture. **(A)** FACS plots indicating the frequency of $HIV_{GAG}{}^+$ cells (CAR^-) 8 days after co-culture with CAR_4 or UTD_4 T cells at the 1:25 E:T ratio. **(B)** Summary of the frequency of $HIV_{GAG}{}^+$ cells at 2, 4, 6 and 8 days after co-culture at 1:25 E:T ratio. Symbols represents the average of 3 distinct donors in duplicate and error bars show ± SEM. **(C)** Cumulative infection calculated by area under the curve from the frequency of $HIV_{GAG}{}^+$ cells at 2, 4, 6 and 8 days after co-culture at 1:100 and **(D)** 1:200 E:T ratios. Data are represented as box and whisker plots and bars show min and max values. Symbols indicate unique donors performed in duplicate. Kruskal-Wallis test and Dunn's multiple comparison test was used to determine significance (**P<0.05, **P<0.01, ***P<0.001, ****P<0.0001*).

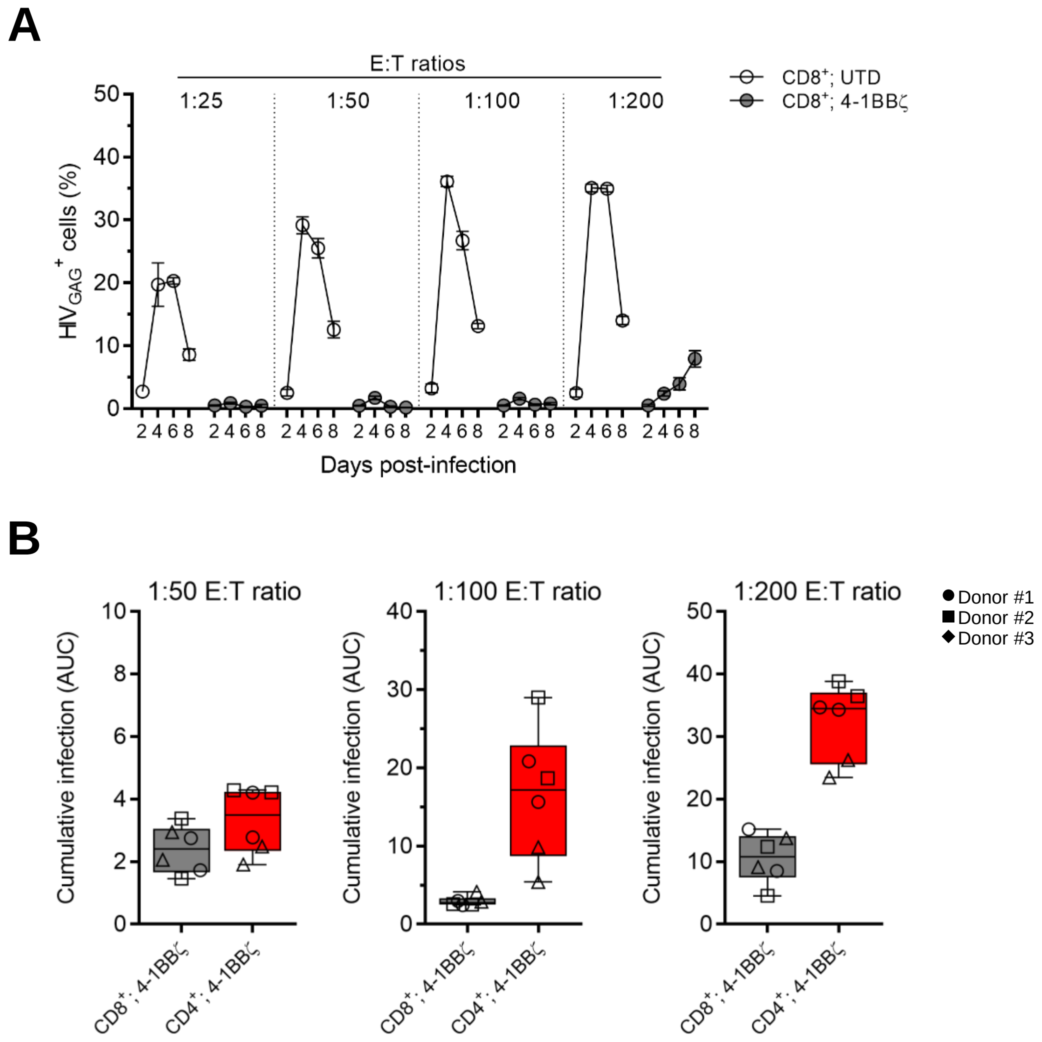

Figure S5. HIV-specific CAR_8 T cells expressing the 4-1BBζ ICD durably suppress *in vitro* virus replication. UTD and HIV-specific CAR_4 and CAR_8 T cells expressing the 4-1BBζ intracellular domain were mixed separately with HIV_{BAL}-challenged $CD4^+$ T cells at the indicated effector-to-target (E:T) ratios, and the level of virus spread was monitored by intracellular staining and flow cytometry for HIV_{GAG} antigen on 2, 4, 6 and 8 days after co-culture. **(A)** Summary of the frequency of HIV_{GAG}^+ (CAR^-) cells at 2, 4, 6 and 8 days after co-culture. Symbols represents the average of 3 donors in duplicate and error bars show ± SEM. **(B)** Cumulative infection calculated by area under the curve from the frequency of HIV_{GAG}^+ cells at 2, 4, 6 and 8 days after co-culture. Data are represented as box and whisker plots and bars show min and max values. Symbols indicate unique donors performed in duplicate.

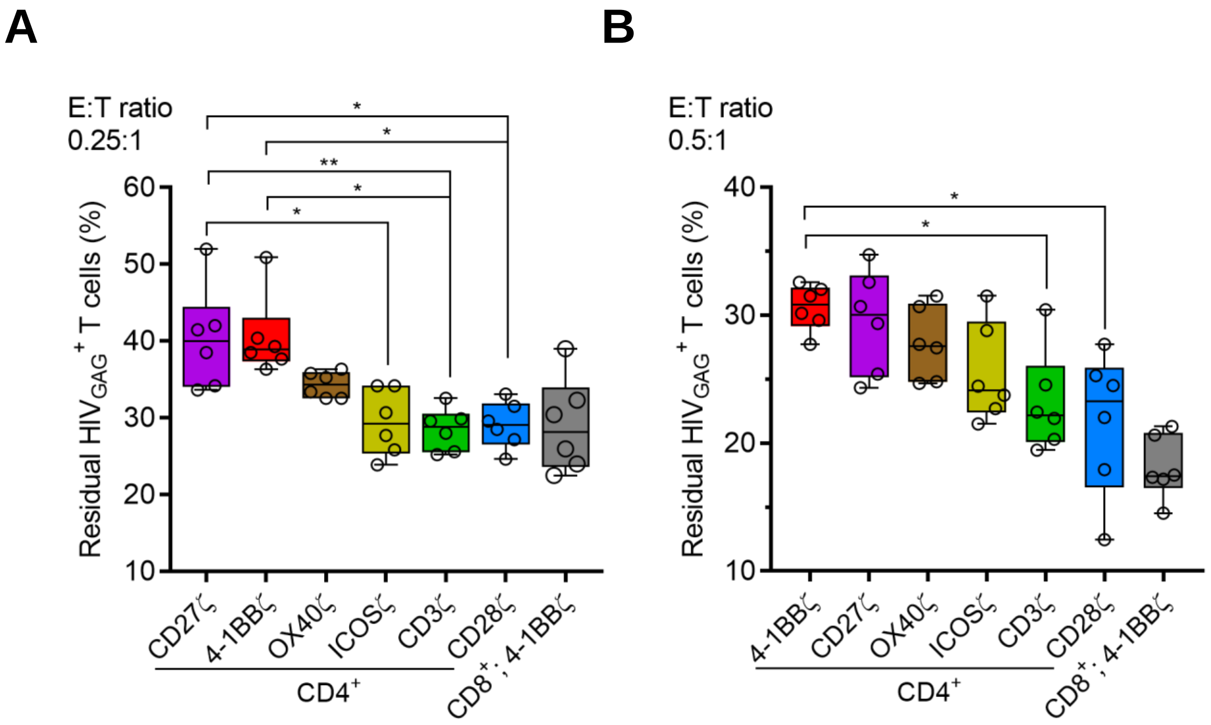

Figure S6. HIV-specific CAR_4 T cells exhibit *in vitro* cytotoxic activity. CellTrace Violet labelled, HIV_{BAL}-infected $CD4^{+}$ T cells (30% HIV_{GAG}^{+}) were cultured with UTD or HIV-specific CAR T cells at the indicated E:T ratios. Frequency of HIV_{GAG}^{+} cells (live CAR^{-} $CD8^{-}$ T cells) was assessed by intracellular staining and flow cytometry for HIV_{GAG} antigen 24 hours later. **(A)** Summary data indicates the frequency of residual HIV_{GAG}^{+} cells that exist after co-culture with CAR T cells at the 0.25:1 and **(B)** 0.5:1 E:T ratio. Data are represented as box and whisker plots and bars show min and max values. Symbols indicate 3 donors performed in duplicate. Kruskal-Wallis test and Dunn's multiple comparison test was used to determine significance (*$P<0.05$, **$P<0.01$).

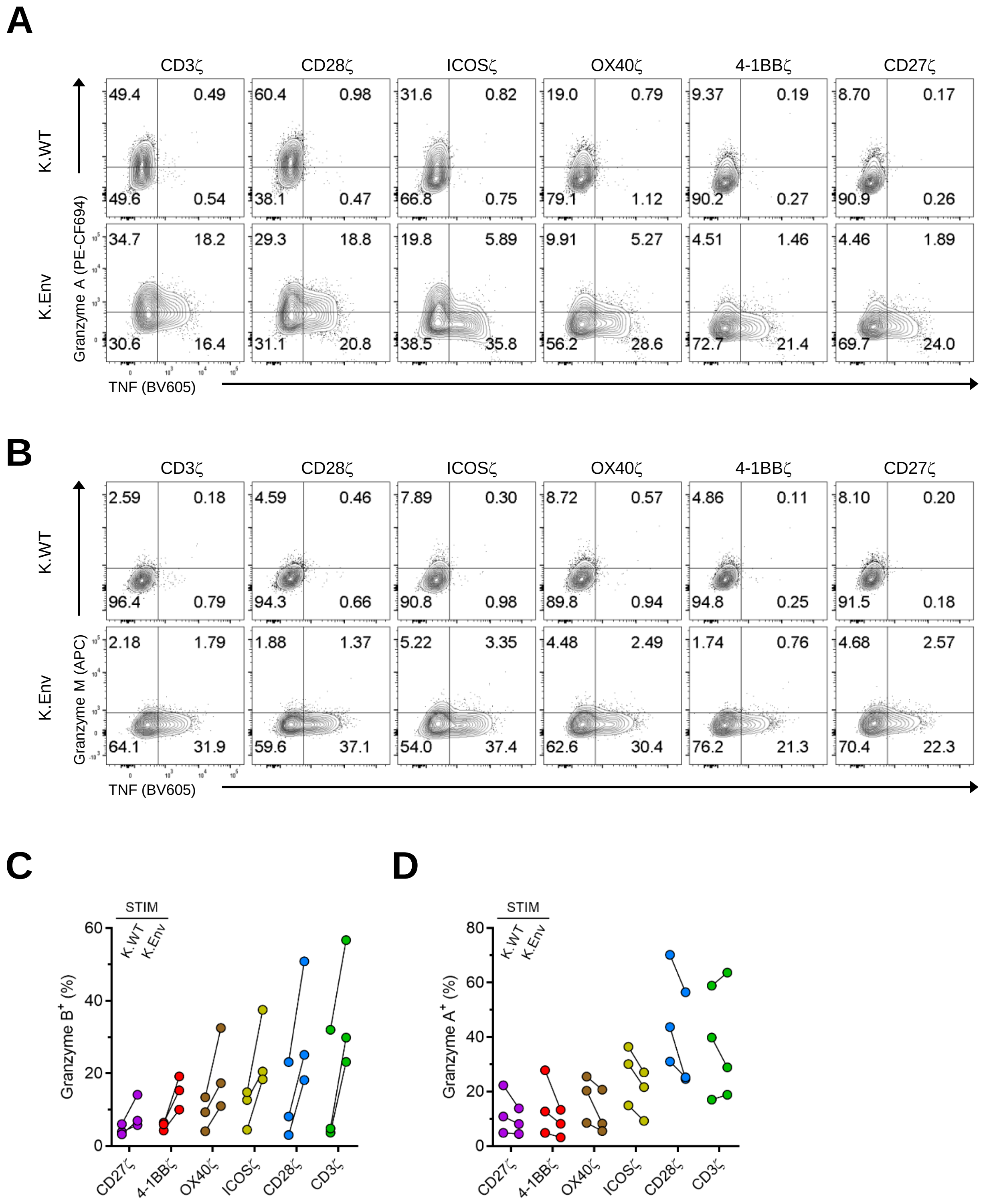

Figure S7. HIV-specific CAR$_4$ T cells differentially express Granzymes after *in vitro* stimulation. After 10 days of culture, each CAR$_4$ T cell type was *in vitro* stimulated with K.Env or wild-type K562 cells (K.WT) and the intracellular expression levels of Granzyme B, A and M were assessed. **(A)** FACS plots show the frequency of Granzyme A and **(B)** Granzyme M expression in CAR$_4$ T cells. Data are representative of 3 distinct donors. **(C)** Summary data showing the change in Granzyme B and **(D)** Granzyme A levels after *in vitro* stimulation. Each symbol represents one donor.

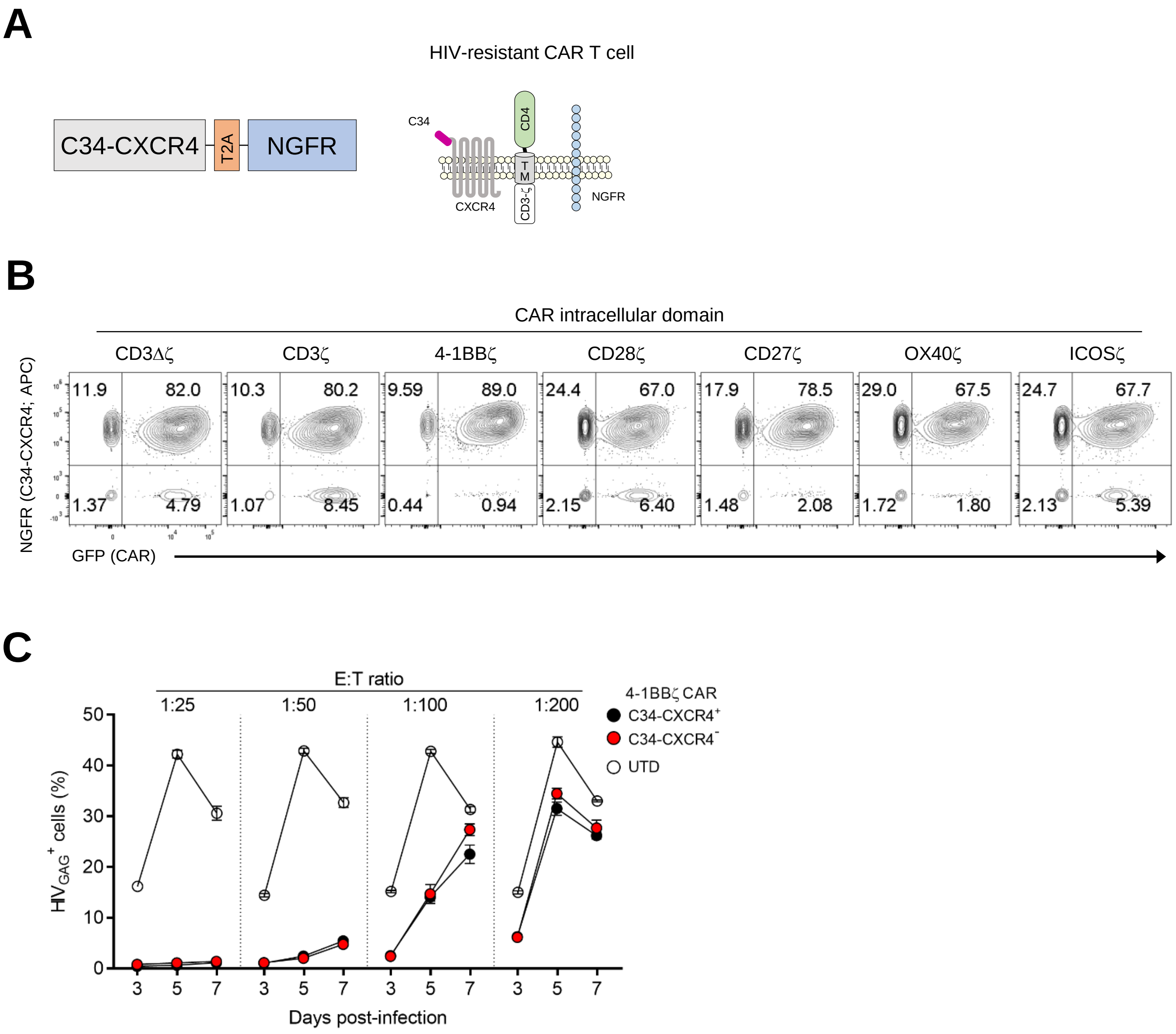

Figure S8. Manufacturing and *in vitro* functional evaluation of HIV-resistant CAR T cells. HIV-specific CAR_4 T cells were co-transduced with lentivirus encoding the HIV fusion inhibitor C34-CXCR4 linked to NGFR by an intervening T2A sequence. Seven days after activation with αCD3/CD28 Dynabeads, T cells were positively selected for NGFR expression using αNGFR antibody coated magnetic beads as described in Materials and Methods. **(A)** Schematic of the C34-CXCR4 construct used to confer HIV-resistance to CAR_4 T cells. **(B)** FACS plots indicate the purity of $NGFR^+$ CAR_4 T cells following magnetic bead selection prior to infusion into HIV-infected humanized mice. **(C)** HIV suppression assay as described in Materials and Methods. Viral outgrowth kinetics when HIV-infected $CD4^+$ T cells are co-cultured with either C34-CXCR4$^+$ or C34-CXCR4$^-$ CAR T cells expressing the 4-1BBζ intracellular domain or UTD. Symbols indicate mean and error bars show ± SEM.

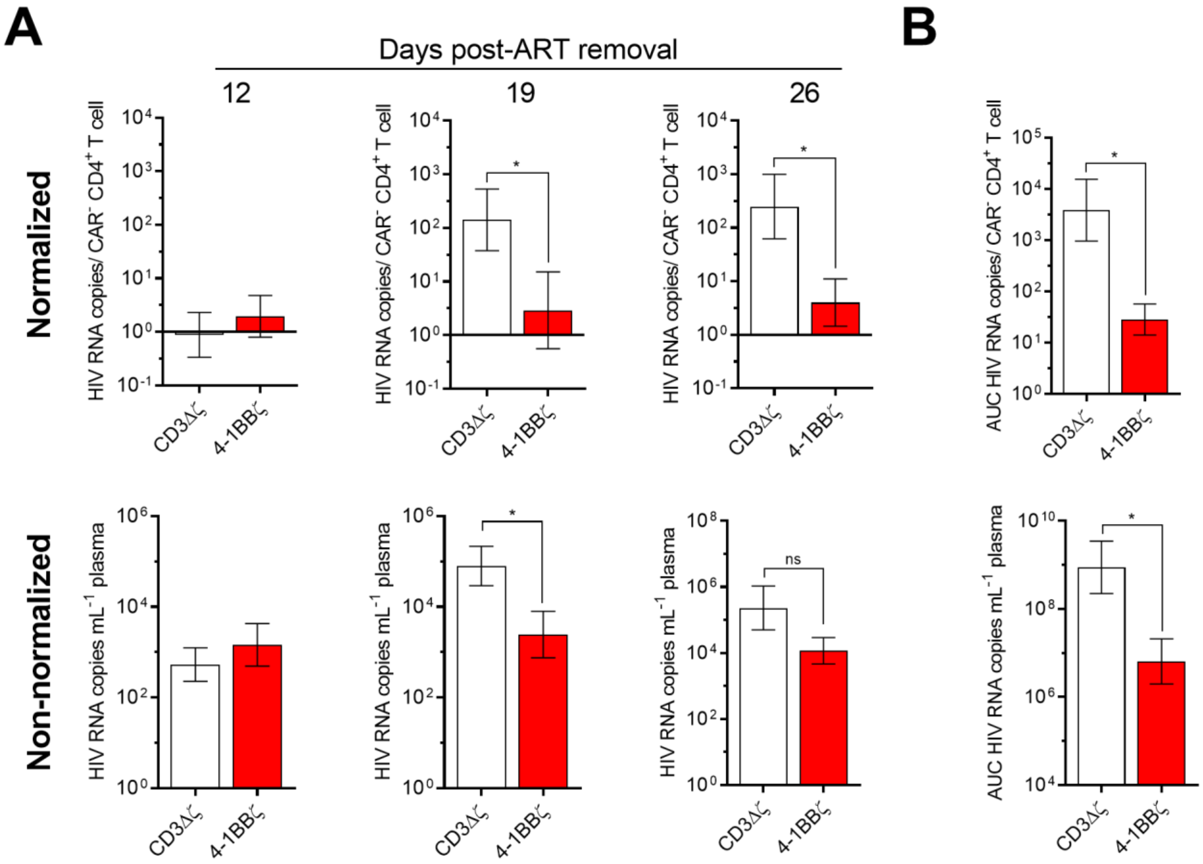

Figure S9. Plasma viral load comparison of normalized and non-normalized values. (A) Viral loads of 4-1BBζ CAR_4 T cell-treated mice compared to control CD3Δζ CAR_4 T cell-treated mice (see also Figure 4). HIV RNA copies mL^{-1} plasma are either normalized to contemporaneous peripheral $CD4^+$ T cell (CAR^-) concentration or non-normalized (i.e raw concentration). **(B)** Cumulative viral load calculated by area under the curve from 5, 12, 19 and 26 days post-ART removal. For all data, bars indicate mean and error bars show ± SEM. Significance was calculated using Wilcoxon rank sum text (*P<0.05)

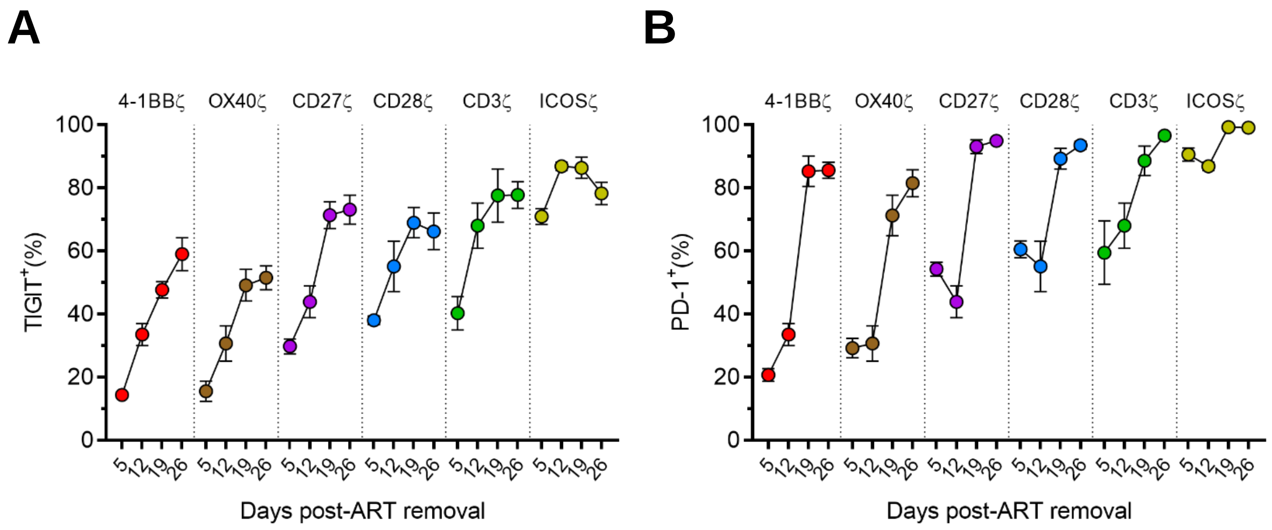

Figure S10. HIV-specific CAR$_4$ T cells expressing TNFR family intracellular domains exhibit lower inhibitory receptor expression post-ART. (A) Longitudinal expression of TIGIT and **(B)** PD-1 on peripheral blood HIV-specific CAR$_4$ T cells post-ART removal in HIV-infected humanized mice. Symbols indicate mean and error bars show ± SEM.

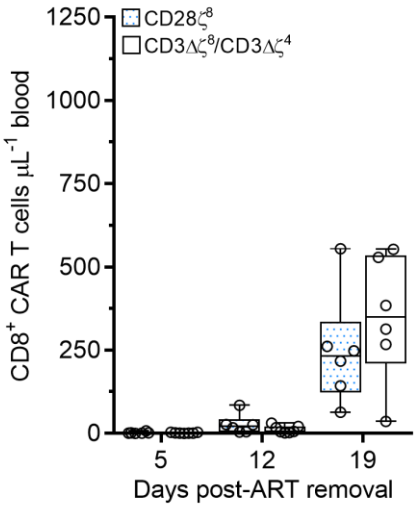

Figure S11. HIV-specific CD28-costimulated CAR_8 T cells fail to expand post-ART removal *in vivo*. Longitudinal concentration of HIV-specific CAR_8 T cells expressing either the CD28ζ or CD3Δζ ICD in peripheral blood following ART withdrawal in HIV-infected humanized mice. Data are represented as box and whisker plots and bars show min and max values. Each symbol denotes one mouse.

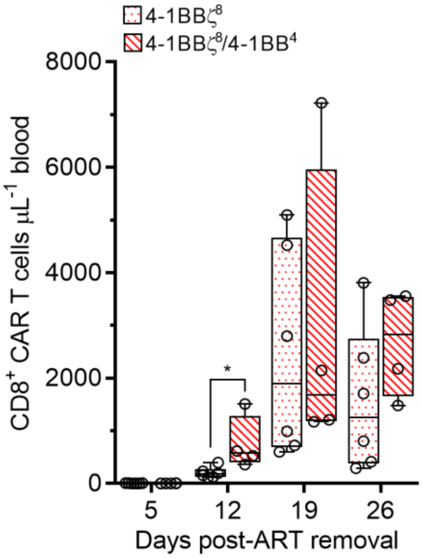

Figure S12. Co-injection of HIV-specific CAR_4 T cells improve early expansion kinetics of 4-1BB-costimulated CAR_8 T cells post-ART removal. NSG mice were infused with CD8-depleted PBMCs from a healthy human donor. Two weeks later, mice initiated daily ART for one week and were infused with autologous, *in vitro* HIV_{BAL}-infected $CD4^+$ T cells. Mice were allocated into 3 groups (n=4-6) based on $CD4^+$ T cell engraftment, and then each mouse received either HIV-resistant (C34-$CXCR4^+$) CAR_8 (4-1BBζ) T cells ($2.5x10^6$ CAR^+ cells), 1:1 ratio of CAR_4 (4-1BBζ) and CAR_8 (4-1BBζ) T cells ($1.25x10^6$ CAR^+/cell type), or 1:1 ratio of inactive control CAR_4 and CAR_8 T cells ($1.25x10^6$ CAR^+/cell type) expressing the CD3Δζ ICD followed by ART interruption. Longitudinal concentration of peripheral CAR_8 T cells after ART interruption. Data are represented as box and whisker plots and bars show min and max values. Each symbol denotes one mouse and Wilcoxon rank sum test was used to calculate significance (*$P<0.05$).

Table S1. Statistical comparison of normalized plasma viral loads (*P* value)

Days post-ART removal	CAR_4	CAR_4 T cell populations					
		CD3Δζ	CD3ζ	CD28ζ	ICOSζ	CD27ζ	OX40ζ
19	4-1BBζ	*0.014	*0.014	*0.014	0.234	0.101	0.035
26	4-1BBζ	*0.035	*0.035	0.467	0.628	0.628	0.180

Significance was calculated using Wilcoxon rank sum test (*P<0.5)

Statistical comparison of 4-1BBζ CAR_4 T cell-treated group and other indicated CAR_4 types (see also Figure 4)
